# Astrocytes express aberrant immunoglobulins as putative gatekeeper of astrocytes to neuronal progenitor conversion

**DOI:** 10.1038/s41419-023-05737-9

**Published:** 2023-04-04

**Authors:** Alice Capuz, Sylvain Osien, Mélodie Anne Karnoub, Soulaimane Aboulouard, Estelle Laurent, Etienne Coyaud, Antonella Raffo-Romero, Marie Duhamel, Amélie Bonnefond, Mehdi Derhourhi, Marco Trerotola, Ikram El Yazidi-Belkoura, David Devos, Monika Zilkova, Firas Kobeissy, Fabien Vanden Abeele, Isabelle Fournier, Dasa Cizkova, Franck Rodet, Michel Salzet

**Affiliations:** 1 Univ. Lille, Inserm, U-1192 - Laboratoire Protéomique, Réponse Inflammatoire et Spectrométrie de Masse-PRISM, 59655 Villeneuve d’Ascq, France; 2 Univ. Lille, Inserm UMR1283, CNRS UMR8199, European Genomic Institute for Diabetes (EGID), Institut Pasteur de Lille, CHU de Lille, 1 place de Verdun, 59000 Lille, France; 3 Laboratory of Cancer Pathology, Center for Advanced Studies and Technology (CAST), University ‘G. D’Annunzio’, Chieti, Italy; 4 Department of Medical, Oral and Biotechnological Sciences, University ‘G. D’Annunzio’, Chieti, Italy; 5 Université de Lille, CNRS, UMR 8576 - UGSF - Unité de Glycobiologie Structurale et Fonctionnelle, 59655 Villeneuve d’Ascq, France; 6 Université de Lille, INSERM, U1172, CHU-Lille, Lille Neuroscience Cognition Research Centre, 1 place de Verdun, 59000 Lille, France; 7 Institute of Neuroimmunology, Slovak Academy of Sciences, Dúbravská cesta 9, 84510 Bratislava, Slovakia; 8 Department of Biochemistry and Molecular Genetics, Faculty of Medicine, American University of Beirut, Beirut, Lebanon; 9 Université de Lille, INSERM U1003, Laboratory of Cell Physiology, 59655 Villeneuve d’Ascq, France; 10 Institut Universitaire de France, 75005 Paris, France; 11 Centre for Experimental and Clinical Regenerative Medicine, University of Veterinary Medicine and Pharmacy in Kosice, Kosice, Slovakia

**Keywords:** Proteomics, Gliogenesis, Regeneration and repair in the nervous system

## Abstract

Using multi-omics analyses including RNAseq, RT-PCR, RACE-PCR, and shotgun proteomic with enrichment strategies, we demonstrated that newborn rat astrocytes produce neural immunoglobulin constant and variable heavy chains as well as light chains. However, their edification is different from the ones found in B cells and they resemble aberrant immunoglobulins observed in several cancers. Moreover, the complete enzymatic V(D)J recombination complex has also been identified in astrocytes. In addition, the constant heavy chain is also present in adult rat astrocytes, whereas in primary astrocytes from human fetus we identified constant and variable kappa chains as well as the substitution lambda chains known to be involved in pre-B cells. To gather insights into the function of these neural IgGs, CRISPR-Cas9 of IgG2B constant heavy chain encoding gene (*Igh6)*, IgG2B overexpression, proximal labeling of rat astrocytes IgG2B and targets identification through 2D gels were performed. In *Igh6* KO astrocytes, overrepresentation of factors involved in hematopoietic cells, neural stem cells, and the regulation of neuritogenesis have been identified. Moreover, overexpression of IgG2B in astrocytes induces the CRTC1-CREB-BDNF signaling pathway known to be involved in gliogenesis, whereas *Igh6* KO triggers the BMP/YAP1/TEAD3 pathway activated in astrocytes dedifferentiation into neural progenitors. Proximal labeling experiments revealed that IgG2B is N-glycosylated by the OST complex, addressed to vesicle membranes containing the ATPase complex, and behaves partially like CD98hc through its association with LAT1. These experiments also suggest that proximal IgG2B-LAT1 interaction occurs concomitantly with MACO-1 and C2CD2L, at the heart of a potentially novel cell signaling platform. Finally, we demonstrated that these chains are synthesized individually and associated to recognize specific targets. Indeed, intermediate filaments Eif4a2 and Pdia6 involved in astrocyte fate constitute targets for these neural IgGs. Taken together, we hypothese that neural aberrant IgG chains may act as gatekeepers of astrocytes' fate.

## Introduction

Immunoglobulin superfamily (IgSF) proteins are implicated in diverse steps of brain development, including neuronal migration, axon pathfinding, target recognition, and synapse formation [1]. High diversity and function of transmembrane and secreted members of IgSF proteins have been reported in the brain [1]. There are about 500 non-antibodies and non-T-cell receptor (TCR) IgSF proteins encoded by the human genome [2]. Among the role of membrane IgSF, neurite extension, neuronal migration, and synaptic plasticity are the most known [3]. Among these IgSF, Thy-1 [4] is of particular interest. Thy-1 presents sequence homology with the variable part of the immunoglobulin heavy chain. Neuronal Thy-1 interaction with the astrocyte αvβ3-integrin–syndecan-4 receptor pair triggers the formation of focal adhesions and stress fibers in astrocytes via RhoA activation [5]. This interaction stabilizes neuronal connections and suppresses axonal regrowth after injury in the astrocyte-rich areas of the adult central nervous system [6]. Recently, the expression of immunoglobulin constant domain genes in neurons of the mouse central nervous system has been demonstrated [7]. The identified IgG3 and IgM membrane forms lack the variable immunoglobulin regions. The corresponding IgG3 and IgM transcripts lack the canonical B-cell transcription initiation site, while alternative transcription start sites have been identified. In our recent work on motor neurons, we also identified IgG2B and IgG2C transcripts, their Fc receptors (CD16 and CD32b) as well as the recombination genes (RAG1 and RAG2) [8]. We established the involvement of CD16 and CD32b in antibody-dependent neurite outgrowth modulation (ADNM) response. Moreover, in astrocytes, we identified alternative proteins issued from long non-coding RNA encoding a variable light chain (so-called Heimdall) and a variable heavy chain [9]. We demonstrated that the Heimdall protein was involved in astrocytes phenotype control [9]. Its inhibition led to neuronal phenotype switch by expression of neuronal progenitor stem cells factors, neurite outgrowth factors, and induced astrocyte prolongments similar to neurites [9].

Taken together, evidence support that true immunoglobulin chains seem to be synthesized in non-B cells. To understand whether a complete mechanism involving antibody production also occurs in astrocytes like in B cells, we investigate by pan-transcriptomic, and proteomic the complete repertoire of IgG chains. The variable heavy chain (IgVH) transcript does not contain the D and J chains but a leader and an RSS sequence, whereas the IgG2B heavy constant chain mRNA (Igh6) starts with a 5’ Kozak sequence and ends with a 3’ sequence coding a transmembrane domain. The constant kappa chain transcript contains a 5’ RSS sequence followed by a joining chain and the *Ighk* sequence whereas the variable kappa light chain starts with a 5’ leader sequence followed by the *Igvk* and a 3’ RSS sequence. We then established the expression of the V(D)J recombination complex in astrocytes. In B cells, this complex is characterized by a gene rearrangement of the V (variable) segment, the J (junction) segment, and the D (diversity) segment for the heavy chain and the V (variable) segment and the J (junction) segment for the light chain. In the literature, this V(D)J process involves a whole enzymatic complex. Finally, we investigated the function of IgG2B in astrocytes using CRISPR-Cas9, overexpression experiments, proximal labeling using BioID, epitopes targeting methods and thus established that these astrocytic IgGs could be involved as gatekeepers of astrocytes fate.

## Materials and methods

### Experimental design

All experiments (transcriptomics, CRISPR-Cas9, western blots, immunofluorescence, shotgun proteomics, BioID) were conducted in biological triplicate to ensure data reproducibility.

### Reagents

Flp-In™ T-REx™ HEK293 cells, DMEM media, Phosphate buffer saline (PBS), heat-inactivated fetal bovine serum (FBS), puromycin, Alexa Fluor^®^ 594-conjugated goat anti-rabbit (A-11012), Streptavidin Alexa Fluor 488 conjugate (S32354), Alexa Fluor^®^ 555-conjugated donkey anti-mouse (A-31570), Alexa Fluor^®^ 647-conjugated goat anti-rat IgG (A-21247), Hoechst 33,342, SuperScript® III kit, pcDNA5 FRT/TO N-ter BirA*Flag vectors, pOG44, biotin, hygromycin B, Streptavidin Ultralink Resin were purchased from Invitrogen Life Technologies (Milan, Italy). Rat astrocytes–adult (RA-a) primary culture, human astrocytes-spinal cord (HA-sp) primary culture, astrocyte medium-animal and astrocyte medium were provided by Sciencell. Water, formic acid (FA), trifluoroacetic acid (TFA), and acetonitrile (ACN) were from Biosolve B.V. (Valkenswaard, the Netherlands). Sodium dodecyl sulfate (SDS), DL-dithiothreitol (DTT), iodoacetamide (IAA), tetracycline, protease inhibitor cocktail, mouse anti-FLAG (F1804), Polybrene infection reagent, rat tail collagen type I, DMSO, FastDAB, astrocyte cell line DI TNC1 were provided by Sigma-Aldrich (Saint-Quentin Fallavier, France). Trypsin/Lys-C Mix, Trypsin Mass Spec Grade, MS-grade TPCK trypsin, DNase RQ1, Go Taq polymerase, pGEM-T easy were purchased from Promega (Charbonnieres, France). Papain Dissociation System was obtained from Worthington Biochem. Corporation (NJ, USA). Anti-GFAP (ab7260) used to assess the purity of astrocyte primary cultures was provided by Abcam. Alexa Fluor 700-conjugated mouse anti-GFAP (NBP2-34413AF700) was from Novus Biologicals. FACS Lysing Solution, BV421-conjugated mouse anti-rat CD45RA (740043) and PE-conjugated mouse anti-rat CD45R (554881) were provided by BD Biosciences. Rabbit anti-GFAP (AB5804) used during immunofluorescence experiments, Mouse anti-GFAP (mab360) used during western blot experiments, Amicon ultracentrifugal filter 10 K and ZipTip C18 were purchased from Millipore. Goat anti-rat IgG2a (A110-109A) was obtained from Bethyl Laboratories, Inc. (Montgomery, TX). Vectashield mounting medium was purchased from Vector Laboratories. NucleoSpin RNA, Mini kit for RNA purification, NucleoSpin Gel and PCR clean-up were obtained from Macherey-Nagel. SMARTer RACE 5’/3’ Kit and SeqAmp DNA Polymerase were purchased from Clontech. Q5 High-Fidelity DNA Polymerase, Quick CIP, T4 DNA ligase, KpnI-HF and BsrGI-HF were provided by NEW ENGLAND BioLabs. LentiCRISPRv2-sgRNA, pVSVg and psPAX2 were from addgene. Mouse anti-CD20 (D-10) (sc-393894) and peroxidase-conjugated mouse anti-IgGk BP (sc-516102) were purchased from Santa Cruz Biotechnology. IgKV antiserum referred to as anti-Heimdall was produced in rabbits by Biotem (Apprieu, France) using the chemically synthesized immunogenic sequence KPGKSPQLLIYYASSLQD coupled to KLH. Rabbit anti-rat IgG (312-005-003), Peroxidase-conjugated donkey anti-goat IgG (705-035-147), Peroxidase-conjugated goat anti-rabbit IgG (111-035-045) and Peroxidase-conjugated goat anti-mouse IgG (115-035-003) were purchased from Jackson ImmunoResearch (West Grove, PA, USA). Mouse anti-Beta-actin (3700 S) was provided by Cell Signaling Technology. Turbonuclease was purchased from BPS Bioscience. Polyjet™ in vitro DNA Transfection reagent was obtained from SignaGen Laboratories. Recombinant Human IL-2 (200-02), recombinant Human IL-1β (200-01B), recombinant Human TNF-α (300-01A), recombinant Human IL-12p70 (200-12), recombinant Human IFN-γ (300-02), recombinant Human IL-6 (200-06) and recombinant Human IL-4 (200-04) were purchased from Preprotech (Rocky Hill, NJ, USA). PlasmoTest^TM^ Mycoplasma Detection Kit was obtained from Invivogen (Toulouse, France).

### Cell culture

DI TNC1 cell line was grown in DMEM supplemented with 10% heat-inactivated FBS, 4 mM l-glutamine, 1 mM sodium pyruvate, 100 U/mL penicillin, and 100 μg/mL streptomycin. Any mycoplasma contamination was excluded using PlasmoTest^TM^ Mycoplasma Detection Kit. Rat astrocytes–adult (RA-a) primary culture and human astrocytes-spinal cord (HA-sp) were cultured in astrocyte medium-animal and astrocyte medium, respectively. All cell lines were maintained at 37 °C in a humidified atmosphere containing 5% CO_2_.

### Isolation and cultivation of rat primary astrocytes

Experiments on animals were carried out according to institutional animal care guidelines conforming to international standards and were approved by the State Veterinary and Food Committee of Slovak Republic (Ro-4081/17-221), and by the Ethics Committee of the Institute of Neuroimmunology, Slovak Academy of Science, Bratislava.

#### Cortex astrocyte cultures

After cervical dislocation (*n* = 11), cerebral cortices of 3–6-day-old Wistar rats were dissected, stripped of their meninges, and mechanically dissociated by repeated pipetting followed by passing through a nylon mesh (70 µm). Cells were plated at a density of 1 × 10^6^ in Petri dishes (60 mm in diameter) pre-coated with 20 µg/ml rat tail collagen, type I (Sigma-Aldrich, St. Louis, Missouri, USA) and cultivated in DMEM containing 10% heat-inactivated FBS and 2 mM l-glutamine, 100 U/mL penicillin, and 100 μg/mL streptomycin at 37 °C, 5% CO_2_ in a water-saturated atmosphere.

#### Spinal cord astrocyte cultures

Meninges were carefully removed from dissected spinal cord tissue. Afterward, 2 ml of papain + DNase solution enzymes (Worthington Biochemical Corporation, USA) were added, gently mixed with 5 ml pipette, and incubated for 15 min at 37 °C. The mixture was triturated and 2.7 ml of EBSS with 300 µl of albumin-ovomucoid inhibitor solution and 150 µl of DNase solution were added. The mixture was again gently triturated followed by centrifugation at 300×*g* for 5 min. Pelleted cells were resuspended in a fresh DMEM containing 10% heat-inactivated FBS, 2 mM l-glutamine, 100 U/mL penicillin, and 100 μg/mL streptomycin, plated at a density of 1 × 10^6^ in Petri dishes (60 mm in diameter) and incubated at 37 °C, 5% CO_2_ in a water-saturated atmosphere.

The medium for both astrocyte cultures (cortex astrocytes and spinal cord astrocytes) was changed twice a week. Mixed glial cultures reached confluence after 8 to 10 days in vitro. Afterward, confluent glial cultures were subjected to mild trypsinization (0.06% trypsin-EDTA). This resulted in the detachment of an intact layer of cells containing astrocytes, leaving undisturbed a population of firmly attached microglia cells [10–12]. The astrocyte cells (cells from the intact layer) were then cultivated and expanded for 20 days in the same cultivation conditions as used for mixed glial cultures, creating unsuitable conditions for more demanding cell populations, such as B cells in culture [13]. The purity of astrocyte cell cultures isolated by this procedure was routinely around 95% by anti-GFAP antibody staining. The confluent astrocyte cultures were frozen in a freezing medium composed of 45% DMEM, 45% FBS and 10% DMSO.

### Flow cytometry analysis to assess B-lymphocytes contamination in astrocyte cultures

One million of the cortex and spinal astrocyte cells were resuspended in 50 μL of PBS and incubated with 5 μL of Alexa Fluor 700-conjugated mouse anti-GFAP (Bio-Techne, USA) BV421-conjugated mouse anti-rat CD45RA and PE-conjugated mouse anti-rat CD45R (Becton Dickinson, USA). After 30 min of incubation in the dark at RT, the cells were fixed with BD FACS Lysing Solution CD45R (Becton Dickinson, USA) and centrifuged at 300×g for 5 min. The cell pellets were resuspended in 400 μL of PBS and analyzed by flow cytometry (BD LSRFortessa™ II cell analyzer).

#### Control staining of rat blood B lymphocytes

B lymphocytes were detected by flow cytometry with BV421-conjugated mouse anti-rat CD45RA and PE-conjugated mouse anti-rat CD45R (high positivity for both markers) (green, double-positive cells).

#### B lymphocyte staining in spinal cord and cortex cell cultures

Gates for positivity were set using an unstained sample, Alexa Fluor 700-conjugated mouse anti-GFAP, BV421-conjugated mouse anti-rat CD45RA and PE-conjugated mouse anti-rat CD45R were used for staining.

### Immunofluorescence

Rat brain and injured spinal cords were harvested, fixed in 4% paraformaldehyde, and bathed in sucrose solution of increasing concentration of 10 to 30% over 3 days. The tissues were then included in 2% cellulose and frozen at −80 °C. Samples were then cut with a cryostat in 12-μm thick sections and dried in a desiccator for 15 min. Sections were then post-fixed fixed in 2% paraformaldehyde for 10 min, rehydrated by 3 baths of PBS 0.1 M; pH 7.4 and saturated for 1 h in PBS 0.1 M; pH 7.4 containing 2% BSA buffer, 0.3% triton, 5% normal donkey serum, and 5% normal goat serum. Alexa Fluor® 647-conjugated goat anti-rat IgG (20 µg/mL) was then added for 1 h. After 2 washes with PBS 0.1 M; pH 7.4 containing 0.1% Tweeen 20 (PBS-T) and 3 washes with PBS 0.1 M; pH 7.4, sections were incubated overnight at 4 °C with mouse anti-GFAP (1:500). After two washes with PBS-T and three washes with PBS 0.1 M; pH 7.4, an incubation for 1 h at 37 °C with Alexa Fluor® 488-conjugated donkey anti-mouse IgG (2 µg/mL) was carried out. After two washes with PBS-T and three washes with PBS 0.1 M; pH 7.4, the nuclei were stained with Hoechst 33,342 (1/10,000). Finally, samples were again washed in 0.1 M PBS pH 7.4 and mounted with Dako fluorescent mounting medium.

Immunofluorescence experiments were carried out to detect IgG in DI TNC1 cells (control or *Igh6* KO), rat primary cortex astrocytes and rat primary spinal astrocytes. After fixation with PFA 4% for 10 min at room temperature, cells were subjected to a cytospin on a glass slide. To block non-specific protein activity, they were then immersed in PBS 0.1 M; pH 7.4 containing 10% normal goat serum (NGS) and 0.2% Triton X-100 for 2 h at room temperature. This was followed by overnight incubation at 4 °C with rabbit anti-GFAP (1:500) and goat anti-rat IgG2A (1:500) (control DI TNC1 cells and primary astrocyte cultures) or Alexa Fluor^®^ 647-conjugated goat anti-rat IgG (20 µg/mL) (control vs *Igh6* KO DI TNC1 cells). Afterward sections were washed three times in 0.1 M PBS pH 7.4 and incubated with Alexa Fluor^®^ 488-conjugated goat anti-mouse and Alexa Fluor^®^ 594-conjugated goat anti-rabbit at room temperature for 2 h. After three additional washes with 0.1 M PBS pH 7.4, the nuclei were stained with 4–6-diaminidino-2-phenylindol (DAPI, 1:200). Finally, samples were again washed in 0.1 M PBS pH 7.4 and mounted with Vectashield mounting medium.

To detect overexpressed IgG2B in HEK293 or DI TNC1 cells grown on coverslips, cells were fixed with PFA 4% for 10 min at room temperature. A blocking step was then performed in PBS 0.1 M; pH 7.4 containing 1% normal donkey serum (NDS), 1% BSA, 1% ovalbumin and 0.2% Triton X-100 for 1 h at room temperature. This was followed by overnight incubation at 4 °C with mouse anti-Flag (1:1000) or in the case of BioID experiments with Streptavidin Alexa Fluor 488 conjugate (1/10,000) to detect proximal proteins that were biotinylated. Dilution of mouse anti-Flag and Streptavidin Alexa Fluor 488 conjugate was performed in the blocking solution. Afterward, sections were washed three times in 0.1 M PBS pH 7.4 and incubated at room temperature for 1 h with Alexa Fluor^®^ 555-conjugated donkey anti-mouse diluted at 2 µg/mL in the blocking buffer. After three additional washes with 0.1 M PBS pH 7.4, the nucleus was stained with DAPI, washed in 0.1 M PBS pH 7.4 and mounted using Dako fluorescent mounting medium.

### Immunocytochemistry

Rat primary cortex astrocytes were fixed with PFA 4% for 10 min at room temperature and cells were subjected to a cytospin on a glass slide. To block non-specific Ab binding, they were then immersed in PBS 0.1 M; pH 7.4 containing 10% normal goat serum (NGS) and 0.2% Triton X-100 for 2 h at room temperature. This was followed by overnight incubation at 4 °C with goat anti-rat IgG2a (1:500). Afterwards, sections were washed three times in 0.1 M PBS pH 7.4 and incubated with Peroxidase-conjugated donkey anti-goat at room temperature for 2 h. After three additional washes with 0.1 M PBS pH 7.4, DAB was added to visualize peroxidase activity. Finally, sections were again washed in 0.1 M PBS pH 7.4 and mounted with a Vectashield mounting medium.

### RT-PCR experiments

RNA extraction was performed with NucleoSpin RNA, Mini kit from 2 million of DI TNC1 cells or rat primary cortex astrocytes or rat primary spinal astrocytes or rat primary adult astrocytes or Human primary spinal astrocytes following manufactory instructions. Two units of DNase RQ1 were used to treat 2 μg of RNA at 37 °C for 30 min. To stop the enzymatic reaction, 2 μL of Stop solution were added and the samples were incubated for 10 min at 65 °C. Reverse-transcription using random primers was then performed using the SuperScript® III kit. cDNAs were stored at −20 °C. A “negative RT” without the addition of the reverse transcriptase was also included as a control to assess gDNA contamination. RT-PCR reactions were then carried out using Go Taq polymerase, the primers listed in Supplementary Table 1 and according to the programs listed in Supplementary Table 2. cDNA fragments of interest were then purified, subcloned into the pGEM-T easy vector, and then sequenced.

### 5’-RACE-PCR

To characterize the 5’-end of rat *Igg2b* cDNA, 5’-RACE-PCR was performed. Full-length cDNA was synthesized using SMARTer RACE 5’/3’ Kit according to the manufacturer’s instructions. PCR reactions were then carried out using SeqAmp DNA Polymerase, Universal Primer Short, and the gene-specific primer 5’- CTC TGA AGG TGC TGT TGT ACT GC -3’. The cycling parameters are described in Supplementary Table 2.

### Illumina RNA sequencing

Total RNAs were extracted using TRI Reagent (Invitrogen) from control and LPS-stimulated (24 h, 48 h) spinal and cortex primary astrocytes. Genomic DNA was removed by DNase I treatment (Sigma-Aldrich). RNAseq experiments were then performed in triplicate on control or LPS-treated primary astrocytes either from the spinal cord or from the cortex. For each RNAseq sample, DNA-depleted total RNA were treated with the Ribo-Zero rRNA removal kit (Illumina) according to the manufacturer’s recommendations. The rRNA-depleted RNA were then used to build the Illumina library using the TruSeq RNA library preparation kit, followed by sequencing on a flowcell S’ de Novaseq a sequencer on Paired-End 25 O. The RNAseq data of each sample were analyzed using both Rockhopper v2.0.3 and SPARTA with the default parameters to calculate the FPKM and TPM values for each coding sequence. The sequencing performed gave 646 million reads generated (323 M fragments) and 645 million reads (322.5 M fragments) for astrocytes from rat cortex and rat spinal cord, respectively.

### CRISPR-CAS9

sgRNAs were designed using the Biology software Benchling (https://benchling.com). Optimization of DNA target specificity and minimization of the off-target effects were obtained as described in ref. [14] and in ref. [15], respectively. sgRNAs were cloned into the plasmid LentiCRISPRv2 [16]. The corresponding lentiviruses were generated in HEK293T cells by co-transfection of LentiCRISPRv2-sgRNA with the packaging plasmids pVSVg [17] and psPAX2 [18]. Lentiviral particles were purified from the HEK293T culture supernatant and utilized for infection of the target cells. Stable knock-out of *Igh6* was obtained by selection in puromycin at 3 µg/ml. The sequences of the sgRNAs targeting Igh6 were as follows: *Igh6* CRISPR #1: 5’-TGTGACATGTAGGGCATGTA-3’ (strand antisense); *Igh6* CRISPR #2: 5’-TGGTGATACAACCAGCTCCA-3’ (strand sense). As a non-target control, the experiment was performed with a sgRNA targeting human Trop2: 5’-GCCACACGGCCGCGCAGGAC-3’. To evaluate the efficiency of the infection, a control, using the empty vector, was also added.

### Western blot

Total protein extracts from DI TNC1 cells, rat primary cortex and spinal astrocytes were first obtained through cell lysis with RIPA buffer (150 mM NaCl, 50 mM Tris, 5 mM EGTA, 2 mM EDTA, 100 mM NaF, 10 mM sodium pyrophosphate, 1% Nonidet P-40, 1 mM PMSF, 1× protease inhibitors). Lysates were then sonicated three times for 5 s with a step on the ice for 30 s between each sonication. After 20 min of centrifugation at 14,000×*g* at 4 °C, the supernatants containing the proteins were collected. Forty micrograms of proteins were reduced in Laemmli buffer containing β-mercaptoethanol, 5 min denatured at 95 °C and separated by SDS-PAGE. Afterward, proteins were transferred onto a nitrocellulose membrane and incubated for 1 h with the blocking buffer (PBS, 0.01% Tween 20 and 5% nonfat dry milk). The primary antibodies diluted in the blocking solution were then added and incubated overnight at 4 °C. After three washes with PBS-Tween 0.01%, secondary antibodies were added and incubated for 1 h at room temperature. Membranes were washed three times with PBS-Tween 0.01% and revealed with an enhanced chemiluminescence kit. Primary antibodies used were mouse anti-CD20 (1/1000), mouse anti-FLAG (1/1000), mouse anti-GFAP (1/1000), rabbit anti-Heimdall (1/1000), rabbit anti-rat IgG (1/500). The respective secondary antibodies used were Peroxydase-conjugated goat anti-mouse (0.03 µg/mL), Peroxydase-conjugated goat anti-rabbit (0.08 µg/mL), and Peroxydase-conjugated mouse anti-IgGk BP. For Beta-actin Western blotting, membranes were first washed with PBS-Tween, stripped with 0.2 M citric acid for 30 min and washed with TBS-0.1% Tween. After saturation with blocking buffer (TBS-Tween containing 5% nonfat dry milk), mouse anti-Beta-actin diluted at 1/1000 in blocking buffer was added and incubated overnight at 4 °C. After three washes with TBS-Tween, membranes were incubated with Peroxydase-conjugated goat anti-mouse (0.03 µg/mL) for 1 h at room temperature. Three washes with TBS-Tween were then performed and revelation with an enhanced chemiluminescence kit was carried out.

### Two-dimensional gel electrophoresis

After two washes of LPS-treated DI TNC1 with an isotonic buffer (250 mM sucrose, 1 mM EDTA, 10 mM Tris-HCl pH 7.5), cells were lysed in a final concentration buffer of 7 M urea, 2 M thiourea, 4% CHAPS, 40 mM DTT, 20 mM spermine base. After 30 min of incubation at RT, the lysate was centrifuged 15 min at 10,000 x g to precipitate nucleic acids. The protein supernatant was then carefully collected, and the protein concentration estimated using a Bradford-type protein assay (Bio-Rad). Finally, 1% by volume of each carrier pharmalytes 3–10 and 4–7 (Bio-Rad) were added, and 200 µg protein aliquots were stored at −80 °C until use. 2D electrophoresis was performed according to [19]. IEF was performed using 7 cm IPG Strips pH 3–10 (Bio-Rad). IPG strips were first reswollen 30 min in 140 µL of solution containing 200 µg of proteins and then incubated overnight with mineral oil. After IEF, the strips were equilibrated for 2 × 15 min in 6 M Urea, 30% w/v glycerol, 2% w/v SDS, 0.125 M Tris, 0.1 M HCl, containing either 50 mM DTT (first equilibration step) or 150 mM iodoacetamide (second equilibration step). The second separation was performed using 10% SDS-PAGE. After migration, the gels were fixed (50% ethanol; 0.2 M orthophosphoric acid) and proteins were detected with colloidal blue staining (1 h in a mixture of 30% methanol, 0.2 M orthophosphoric acid, and 170 g/L ammonium sulfate, before incubation in the same solution to which 0.66 g/L of G250 brilliant blue had been added). Then, membranes were used for western blot.

### Filter-aided sample preparation (FASP) protein digestion

Forty micrograms of protein cell extracts were reduced using an equivalent volume of reduction buffer (dithiothreitol-DTT 0.1 M) for 40 min at 56 °C. After adjusting the volume to 200 µL with denaturing buffer (8 M Urea, 0,1 M Tris-HCL, H_2_O), the samples were centrifuged at 14,000 × *g* for 30 min. Two hundred microliters of the denaturing buffer were again added and followed by centrifugation at 14,000 × *g* for 30 min. The filtrate was discarded and 100 µL of alkylation solution (0.05 M Iodoacetamide in denaturing buffer) was added. The samples were then incubated in the dark for 20 min at room temperature and centrifuged at 14,000 × *g* for 25 min. One hundred microliters of 50 mM Ammonium Bicarbonate Buffer were added and the samples were centrifuged at 14,000 × *g* for 25 min. This step was repeated two times. One hundred microliters of Ammonium Bicarbonate buffer 50 mM were added and the samples were centrifuged at 14,000 × *g* for 25 min. This step was repeated twice. Filters were transferred into a new collection tube and 1.6 µg of Trypsin was loaded. After overnight incubation at 37 °C, samples were centrifuged at 14,000 × *g* for 25 min and filters were washed with 50 µL of 0.5 M NaCl. After centrifugation at 14,000 × *g* for 25 min, 10 µL of H_2_O-5% TFA were loaded to stop the digestion, and samples were dried using SpeedVac. After their resuspension with 20 µL of H_2_O-0.1%TFA, samples were desalted using C18 Millipore ZipTip and eluted with 20 µl of elution solution (80% ACN/20% H_2_O-0.1%TFA). The solution was then dried using the SpeedVac. Dried samples were solubilized in a resuspension solution (2% ACN/80% formic acid 0.1%) before LC-MS/MS analysis.

### Shotgun proteomics data analysis

RAW data obtained from the nLC-MS/MS run were treated using MaxQuant v1.6.1.0 using the LFQ annotation of the protein identified. Proteins were identified by searching MS and MS/MS data against the Decoy version of the complete proteome for *Rattus norvegicus* of the UniProt database (Release June 2014, 33,675 entries) combined with 262 commonly detected contaminants. Statistical analyses were carried out using Perseus software after filtering for “reverse”, and “contaminant” proteins. For the comparison between control and treated groups, a *t* test was performed with a permutation-based FDR of 0.05, and *p* values less than 0.05 were considered statistically significant. A heatmap of differentially expressed proteins across the two different groups was generated. Gene ontology (GO) analysis was performed using ClueGO [20], on Cytoscape v3.7.1 [21].

### Proteogenomic analyses

The AltProt database of rat is a prediction of the possible start codon around the classical Open Reading Frame (ORF), permitting the prevision of proteins on UTR, overlapping between UTR and coding sequence (CDS), and shift of ORF in +2 and +3 and conserving an initiator AUG codon. This database was combined with the reference Uniprot database on the same FASTA file. Label-Free Quantification (LFQ) was performed by MaxQuant 1.5.6.5. During this analysis, principal parameters were assigned as follows: Trypsin digestion with maximum missed cleavage of 2, carbamidomethylation as a fixed modification, and oxidation as a variable modification. The first search peptide tolerance was adjusted at 20 ppm and the main search peptide at 6 ppm. Finally, the minimum peptide length was restricted to 6 amino acids. The length of this kind of protein, a mean of 50 to 100 amino acids, obliges to decrease the number of unique peptides identified at 1. Statistical analysis was performed with Perseus 1.5.5.3, log2(x) was then realized and results were filtrated to eliminate identification by site as well as reverse and potential contaminants. Significant variations between samples were assessed by *t* test. Filtration for AltProt was applied to keep only the AltProt identified with a unique peptide and no classical protein redundancy on Majority ID. Variation of quantification was revealed by hierarchical clustering with Euclidian distance measurement. Identification of AltProt was performed using BlastP and non-redundant protein sequences to find their sequence homology with canonical and unknown proteins. The gene accession numbers were used to retrieve mRNA or ncRNA sequences from the Ensembl database.

### Subnetwork enrichment pathway analysis

Using Elsevier’s Pathway Studio (version 11.0//Elsevier), all relationships between the differentially expressed proteins among all conditions were depicted based on the Ariadne ResNet [22]. For proteins identified in the shotgun analysis, the Subnetwork Enrichment Analysis (SNEA) algorithm was used to detect the statistically significant altered biological pathways in which the identified proteins are involved. This algorithm uses Fisher’s statistical test to detect any non-random associations between two categorical variables organized by a specific relationship. Also, this algorithm starts by creating a central “seed” from all the relevant identities in the database and builds connections with associated entities based on their relationship with the seed. SNEA compares the subnetwork distribution to the background distribution using a one-sided Mann–Whitney *U* test and calculates a *p* value; thus, representing a statistical significance between different distributions. In all analyses that we performed, the GenBank ID was used to form experimental groups based on the different conditions present for analysis. The pathway networks were reconstructed based on biological processes and molecular functions for every single protein, along with its associated targets.

### Cloning of rat astrocyte IgG2B coding sequence in pcDNA5 FRT/TO C-ter BirA*Flag expression vector

Rat astrocyte IgG2B coding sequence was cloned into pcDNA5 FRT/TO C-ter BirA*Flag vector. All clones were sequenced and verified before co-transfection with pOG44 in Flp-In™ T-REx™ HEK293 cells. The construction includes a signal peptide and the sequence coding the IgG2B transmembrane form (ATGGAGTTTGGGCTGAGCTGGGTTTTCCTTGTTGTTATTTTACAAGGTGTCCAGTGTG CC CA*AACAAC AGCCCCATCT GTCTATCCAC TGGCTCCTGG ATGTGGTGAT ACAACCAGCT CCACGGTGAC CCTGGGATGC CTGGTCAAGG GCTATTTCCC TGAGCCAGTC ACCGTGACCT GGAACTCTGGAGCCCTGTCC AGCGATGTGC ACACCTTCCC AGCTGTCCTG CAGTCTGGGC TCTACACTCT CACCAGCTCA GTGACCTCCA GCACCTGGCC CAGCCAGACC GTCACCTGCA ACGTAGCCCA CCCGGCCAGCAGCACCAAGG TGGACAAGAA AATTGAGCGC AGAAATGACA ACATTGGACA CAAAAGC CCTACATGCC CTACATGTCA CAAATGCCCA GCTCCTGAAC TCTTGGGTGG ACCATCCGTC TTCATCTTCCCCCCAAAGCC CAAGGACATC CTCTTGATCT CCCAGAACGC CAAGGTCACG TGTGTGGTGG TGGATGTGAG CGAGGAGGAG CCGGACGTCC AGTTCAGCTG GTTTGTGAAC AACGTAGAAG TACACACAGCTCAGACACAA CCCCGTGAGG AGCAGTACAA CAGCACCTTC AGAGTGGTCA GTGCCCTCCC CATCCAGCAC CAGGACTGGA TGAGCGGCAA GGAGTTCAAA TGCAAGGTCA ACAACAAAG CCCTCCCAAGCCCCATCGAG AAAACCATCT CAAAACCCAA AGGGCTAGTC AGAAAACCAC AGGTATACGT CATGGGTCCA CCGACAGAGC AGTTGACTGA GCAAACGGTC AGTTTGACCT GCTTGACCTC AGGCTTCCTCCCTAACGACA TCGGTGTGGA GTGGACCAGC AACGGGCATA TAGAAAAGAA CTACAAGAAC ACCGAGCCAG TGATGGACTC TGACGGTTCT TTCTTCATGT ACAGCAAGCT CAATGTGGAA AGGAGCAGGTGGGATAGCAG AGCGCCCTTC GTCTGCTCCG TGGTCCACGA GGGTCTGCAC AATCACCACG TGGAGAAGAG CATCTCCCGG CCTCCG* GGG CTAGAAGTGGA TGATGATTGT GCTGAGGCTC AGGACGGGGAGCTGGACGGG CTCTGGACGA CCATCACCAT CTTCATCAGC CTCTTCCTGC TCAGTGTGTG CTACAGTGCC TCCATCACAC TCTTCAAGGT AAAGTGGATC TTCTCCTCAG TGGTGGAGCT GAAGCAGACAATC TCCC CTGACTACAG AAACATGATT GGTCAAGGAG CC TAG). As a control, another construction displaying a signal peptide and the sequence coding for the transmembrane domain only was built. (ATGGAGTTTGGGCTGAGCTGGGTTTTCCTTGTTGTTATTTTACAAGGTGTCCAGTGT GG G CTAGAAGTGGA TGATGATTGT GCTGAGGCTC AGGACGGGGAGCTGGACGGG CTCTGGACGA CCATCACCAT CTTCATCAGC CTCTTCCTGC TCAGTGTGTG CTACAGTGCC TCCATCACAC TCTTCAAGGT AAAGTGGATC TTCTCCTCAG TGGTGGAGCT GAAGCAGACAATC TCCC CTGACTACAG AAACATGATT GGTCAAGGAG CC TAG).

### Overexpression of rat astrocyte IgG2B-Flag in DI TNC1 cells

DI TNC1 cells were plated in six-well plates and grown in a complete medium until they reached about 80% confluence. One hour before the transfection, the medium was renewed. One microgram of the construct was mixed with 50 µL of DMEM-free medium and 3 µL of PolyJet were mixed with 50 µL of DMEM-free medium in another tube. The two solutions were mixed and after an incubation of 15 min at room temperature, the mixture was added to the cells. After overnight incubation, the medium was replaced by the fresh complete medium. After 24, 48, or 72 h, western blots were carried out.

### Characterization of IgG2B patterns in HEK293 cells

BioID samples were prepared as follows. Briefly, Flp-In™ T-REx™ HEK293 cells were grown in DMEM supplemented with 10% FBS GlutaMAX™ and penicillin–streptomycin (1×). Using the Flp-In system, Flp-In™ T-REx™ HEK293 stably expressing BirA*Flag alone and signal peptide with transmembrane domain (for control samples), or C-terminally tagged IgH bait proteins were generated by co-transfecting pOG44 with pcDNA5 FRT/TO IgH BirA*Flag bait protein sequence plasmid. After selection (DMEM + 10% FBS + P/S + 200 μg/ml hygromycin B), three independent replicates of two 150 cm^2^ plates of sub-confluent (60%) cells were incubated for 24 h in complete media supplemented with 1 μg/ml tetracycline (Sigma), and 50 μM biotin. Cells were collected and pelleted by centrifugation at 300 × *g* for 3 min. After two washes with PBS, dried pellets were snap frozen. Each cell pellet was resuspended in 5 ml of lysis buffer (50 mM Tris-HCl pH 7.5, 150 mM NaCl, 1 mM EDTA, 1 mM EGTA, 1% Triton X-100, 0.1% SDS, 1:500 protease inhibitor cocktail, 1:1000 Turbonuclease) and incubated on an end-over-end rotator at 4 °C for 1 h, briefly sonicated to disrupt any visible aggregates, then centrifuged at 45,000 × *g* for 30 min at 4 °C. The supernatant was transferred to a fresh 15 mL conical tube. Twenty-five microliters of packed, pre-equilibrated Streptavidin Ultralink Resin were added, and the mixture was incubated for 3 h at 4 °C with end-over-end rotation. Beads were pelleted by centrifugation at 300 × *g* for 2 min and transferred with 1 mL of lysis buffer to a fresh tube. Beads were washed once with 1 mL of lysis buffer and twice with 1 mL of 50 mM ammonium bicarbonate (pH = 8.3), then transferred in ammonium bicarbonate to a fresh centrifuge tube and washed two more times with 1 ml of ammonium bicarbonate buffer. Tryptic digestion was performed by incubating the beads with 1 μg MS-grade TPCK trypsin dissolved in 200 μl of 50 mM ammonium bicarbonate (pH 8.3) overnight at 37 °C. The following morning, 0.5 μg MS-grade TPCK trypsin was added to the beads and incubated for 2 additional hours at 37 °C. Following centrifugation at 2000 × *g* for 2 min, the supernatant was collected and transferred to a fresh tube. Two additional washes were performed with 150 μL of 50 mM ammonium bicarbonate and pooled with the first eluate. The sample was lyophilized and resuspended in buffer A (2% ACN 0.1% formic acid). 1/3rd of each sample was analyzed per mass spectrometer run.

### BioID data acquisition

The samples were separated by online reversed-phase chromatography using a Thermo Scientific Easy-nLC1000 system equipped with a Proxeon trap column (75 μm ID × 2 cm, 3 μm, Thermo Scientific,) and a C18 packed-tip column (Acclaim PepMap, 75 μm ID × 50 cm, 2 μm, Thermo Scientific). The digested peptides were separated using an increasing amount of acetonitrile in 0.1% formic acid from 2 to 30% for 2 h at a flow rate of 300 nL/min. A voltage of 2.4 kV was applied by the liquid junction to electrospray the eluent using the nanospray source. A high-resolution mass spectrometer Q-Exactive^TM^ Thermo Scientific^TM^ was coupled to the chromatography system to acquire the ten most intense ions of MS1 analysis (Top 10) in a data-dependent mode. The MS analyses were performed in positive mode at a resolving power of 70,000 FWHM, using an automatic gain control target of 3e6, the default charge state was set at 2 and a maximum injection time at 120 ms. For full-scan MS, the scan range was set between *m/z* 300 and 1600. For ddMS², the scan range was between *m/z* 200 to 2000, 1 Microscan was acquired at 17,500 FWHM, an AGC was set at 5e4 ions and an isolation window of *m/z* 4.0 was used.

### BioID data analysis

The proteins were identified by comparing all MS/MS data with the *Homo sapiens* proteome database (Uniprot, release March 2020, Canonical+ Isoforms, comprising 42,360 entries), using the MaxQuant software version 1.5.8.3. The digestion parameters were defined using trypsin with 2 maximum missed cleavages. The oxidation of methionine and N-terminal protein acetylation were defined as variable modifications. Label-free quantification (LFQ) was done while keeping the default parameters of the software. As for initial mass tolerance, 6 ppm were selected for MS mode, and 20 ppm were set for fragmentation data to match MS/MS tolerance. The identification parameters of the proteins and peptides were performed with a false discovery rate (FDR) at 1%, and a minimum of 2 unique peptides per protein. The LFQ values from the 20 control runs (regrouping FlagBirA* and BirA*Flag alone samples), were collapsed to the three highest values for each given ID. These three values were defined as the control group for comparison. The statistical analysis was done using Perseus software (version 1.6.2.3). Briefly, the LFQ intensity of each sample was downloaded in Perseus and the data matrix was filtered by removing the potential contaminants, reverse, and only identified by site. The data were then transformed using the log2(x) function. Only preys with detected values in all three replicates of a given bait protein were kept for further analysis. Missing values (not detected) were then replaced by the minimal valid LFQ value separately for each column. Two-sample Student’s *t* test was then performed comparing all three biological replicates of the bait against the three control runs. High-confidence proximal labeling interactors were defined with a cut-off of *p* < 0.05. Detailed experimental values are reported in Supplemental Data 7.

## Results

### Primary cell culture devoid of B cells

**Fig. 1:**
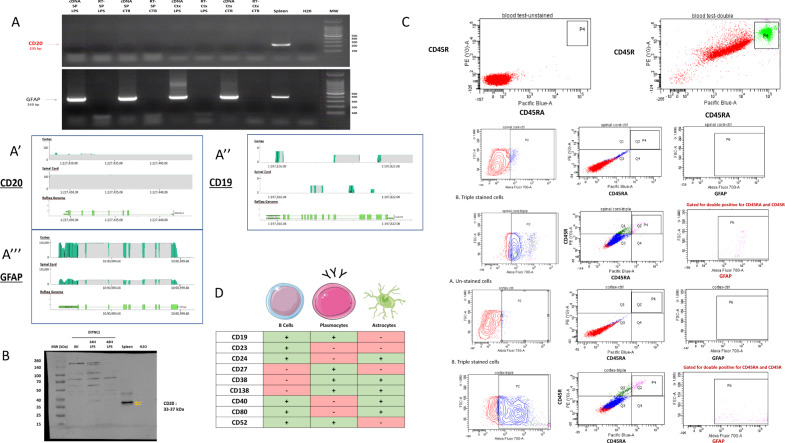
Validation that primary cultures of the cortex and spinal astrocytes are devoid of B cells. **A** RT-PCR amplification of *Cd20* in rat DI TNC1 astrocyte cell line and rat primary cortex and spinal astrocytes stimulated or not with 200 ng/mL of LPS. cDNA from the spleen served as a positive control. The experiment was also performed on a negative reverse transcriptase sample to rule out genomic DNA contamination. H_2_O: negative control. **A’** RNAseq analyses of *Cd20*, **A”** *Cd19* or **A”’** *Gfap* in rat primary cortex and spinal cord astrocytes. **B** Western blot analyses of CD20 in spleen and DI TNC1 cells stimulated or not with 200 ng/mL of LPS during 24 h and 48 h. **C** Flow cytometry analyses of primary cultures of the cortex and spinal astrocytes with CD45R–CD45RA double labeling and CD45R–CD45RA–GFAP triple labeling. Rat blood served as a positive control. **D** Comparison of CD markers found in B lymphocytes, plasmocytes and astrocytes (RNAseq data).

We previously detected the presence of IgG in astrocytes from sections of the injured spinal cord [23]. This experiment was performed 24 h after anti-CD20 treatment to exclude the presence of B cells. However, resident B cells like plasmocytes can still be there [24]. Thus, to exclude recaptured IgGs by astrocytes from blood circulation, we decided to perform our study on primary astrocyte cultures (spinal cord and cortex) from 3 days old rat pups. Three days old rat pups are devoid of B cells [25]. We also used a cell line of rat astrocytes (DI TNC1). All these astrocytes (primary cultures or cell lines) were stimulated or not with 200 ng/mL of lipopolysaccharides (LPS) or a cocktail of cytokines to mimic the inflammation that occurs during spinal cord injury [23, 26, 27]. Moreover, to ensure that our cultures were devoid of B cells, RT-PCR was performed to amplify *Cd*20 transcripts (Fig. 1A). cDNA from the spleen served as an internal control. In primary astrocytes and DI TNC1 cell line, no amplification was obtained for *Cd*20 mRNA (Fig. 1A). This was confirmed by RNAseq analyses. In RNAseq, only 16 reads were aligned with the first three exons of Ms4a1 (chromosome 1: 227,429,596-227,441,442 positions encoding for the CD20) in astrocytes from the cortex and none from the ones of the spinal cord (Fig. 1A’ and Supplementary Data 1). Thus, the coverage of all exons was not observed for *Cd*20, suggesting that *Cd*20 was not expressed in the astrocytes. Its expression was also analyzed by Western blot and no 33-37 kDa bands corresponding to CD20 were observed in astrocytes contrary to B cells (Fig. 1B). Moreover, no reads were observed for plasmacytic marker CD19 in RNAseq (Fig. 1A”). By contrast, RT-PCR experiments performed with primers directed against the cDNA sequence encoding for the Glial fibrillary acidic protein (GFAP), an astrocyte marker, allowed the amplification of a fragment of interest at the expected size of 1292 bp (Supplementary Fig. 1A). The nature of the transcript was confirmed by sequencing and RNAseq. Alignment with the *Gfap* gene on chromosome 10: 90,988,762-91,001,435 showed a complete and deep coverage with a depth of around 100,000 reads for the cortex and 45,000 reads for the spinal cord (Fig. 1A”’). FACS was performed on rat blood with anti-CD45R and CD45RA and confirmed the presence of B cells in the blood (Fig. 1C). Surprisingly, some cortex and spinal astrocytes exhibited staining with CD45R and CD45RA. However, triple staining confirmed that these cells were GFAP-positive (Fig. 1C). Additional RNAseq analyses allowed the comparison between CD markers of the different B-cell populations and astrocyte markers. This revealed that astrocytes are CD19, CD23, CD27, and CD52-negative but express some plasmocyte markers such as CD38 and CD138 (Fig. 1D). Altogether, this points out the existence of a subpopulation of astrocytes sharing some common features with plasmocytes. To verify the astrocytic nature of the cells, the detection of GFAP by western blot was carried out and confirmed the presence of its band at 55 kDa (Supplementary Fig. 1B). Moreover, additional bands corresponding to cleaved forms of GFAP were also detected which is in line with previous reports from literature [28]. Supporting the fact that a specific subpopulation of astrocytes exists, recent data reveal the presence of an astrocyte-like population in the spleen [29, 30]. Our data support that the study determined a faint GFAP expression in the spleen lymphoid center. Although smaller, the bands obtained at 40 and 45 kDa could correspond to the truncated form of GFAP (Supplementary Fig. 1B).

### Immunoglobulin chains Identification

**Fig. 2:**
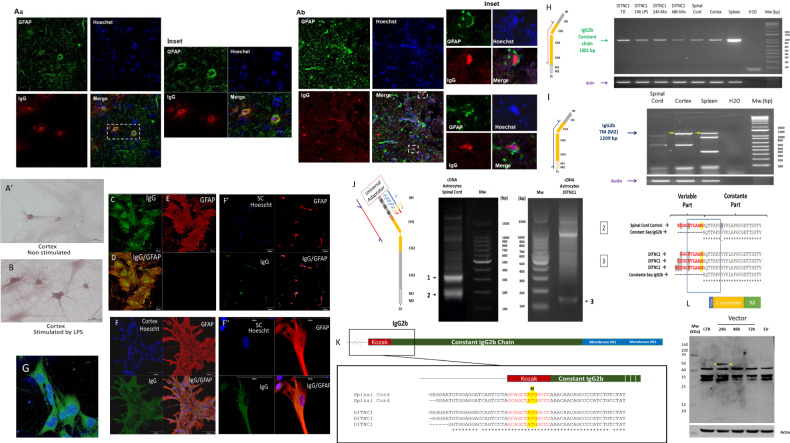
Identification of immunoglobulin constant heavy chain in astrocytes. **A** Immunofluorescence experiments performed on brain (**a**) and SCI (**b**) slices using anti-GFAP and anti-IgG. The nuclei were stained with Hoechst. Immunocytochemical labeling of rat primary cortex astrocytes control (**A’**) or stimulated with 200 ng/mL of LPS (**B**) using anti-IgG or (**C**–**E**) Immunofluorescence with anti-GFAP and anti-IgG performed on rat primary cortex astrocytes control. **F** Rat primary cortex astrocytes stimulated with 200 ng/mL of LPS during 48 h. (**F’**) Rat primary spinal astrocytes stimulated with 200 ng/mL of LPS during 48 h. (**F’'**) Magnification of the double staining observed in rat primary spinal astrocytes. **G** Immunofluorescence with anti-IgG carried out on DI TINC1 cells. **H** RT-PCR amplification of the complete sequence coding IgG2B constant heavy chain in rat DI TNC1 astrocyte cell line stimulated or not with 200 ng/mL of LPS during 24 h or a mix of cytokines for 24 h and 48 h as well as in rat primary cortex and spinal astrocytes. **I** Amplification of the mRNA coding the IgG2B transmembrane form. cDNA from the spleen served as a positive control. The experiment was also performed on a negative reverse transcriptase sample to rule out genomic DNA contamination. H_2_O: negative control. **J** 5’-RACE-PCR was performed on a cDNA library of DI TNC1 cells or astrocytes from the spinal cord to amplify the whole *Igg2B* mRNA. Three bands were amplified and sequenced. This revealed that no variable coding sequence was linked to IgG2B coding sequence. However, the presence of a Kozak sequence flanking the 5’-end of IgG2B coding sequence was identified in bands 2 and 3, indicating that this transcript encoded a free IgG2B constant chain. **K** 5’ Kozak sequence of the IgG2B sequence **L** Overexpression of rat astrocyte IgG2B transmembrane form in fusion with Flag Tag in DI TNC1 cells. Western blot analysis using an antibody directed against the Flag tag was carried out 24 h, 48 h, or 72 h after transfection. Non-transfected cells and transfection with an empty vector served as negative controls.

**Fig. 3:**
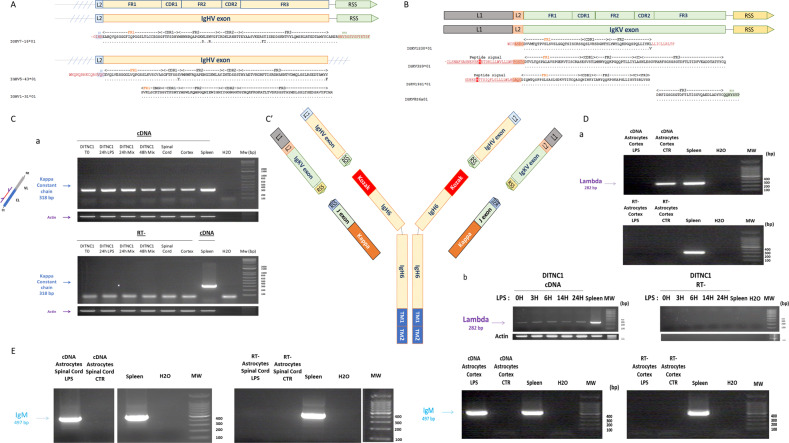
Characterization of mRNA coding variable chains in DI TNC1 cells and rat primary cortex and spinal astrocytes by transcriptomic approaches (RNAseq and RT-PCR). **A** Schematic representation of the variable heavy chain transcripts (*IgHV7.16.01*, *IgHV5-43.01*, and *IgHV1.31.01*) identified. The complete sequences are presented in Supplementary Fig. 4A, B. **B** Schematic representation of the variable Kappa chain mRNA (IgKV) identified with the leader and the RSS sequences. **Ca** RT-PCR amplification of the complete sequence coding IgK constant light chain in rat DI TNC1 astrocyte cell line stimulated or not with 200 ng/mL of LPS during 24 h or a mix of cytokines for 24 h or 48 h as well as in rat primary cortex and spinal astrocytes. Upper panel: Amplification of the mRNA coding the IgG2B secreted form. cDNA from spleen served as a positive control. The experiment was also performed on a negative reverse transcriptase sample to rule out genomic DNA contamination. H_2_O: negative control. **b** 5’ RACE-PCR amplification of *Igk*. The complete sequence is presented in Supplementary Fig. 4A. **c** schematic representation of IgKV. **C’** schematic presentation of the neural Immunoglobulin, **D** RT-PCR amplification of *IgL* mRNA in rat primary cortex astrocytes and DI TNC1 cells stimulated or not with 200 ng/mL of LPS. cDNA from spleen served as a positive control. The experiment was also performed on a negative reverse transcriptase sample to rule out genomic DNA contamination. H_2_O: negative control. **E** RT-PCR amplification of *Igh*m transcripts in primary spinal and cortex astrocytes stimulated with 200 ng/mL of LPS. The complete sequence is presented in Supplementary Fig. 4B. cDNA from spleen served as a positive control. The experiment was also performed on a negative reverse transcriptase sample to rule out genomic DNA contamination. H_2_O: negative control.

In line with our previous data, we established the presence of IgG in a subset of astrocyte population not only in the spinal cord but also in the brain (Fig. 2A). Interestingly, these IgGs were detected close to the nucleus. To confirm the presence of immunoglobulin chains in astrocytes, we performed immunocytochemistry (ICC) and immunofluorescence (IF) experiments on rat primary cortex and spinal astrocytes. ICC using an anti-IgG associated with peroxidase revealed immunoreactivity in normal astrocytes (Fig. 2A’) which was increased when stimulated with LPS (Fig. 2B). We noticed that under LPS stimulation, the number of astrocytes reactive to anti-IgG was higher and the immunolabelling was present in the cell body of the astrocytes but also at the level of their prolongments (Fig. 2B). Similar results were obtained using immunofluorescence experiments performed on astrocytes from the spinal cord. Immunofluorescence for IgG (green) was detected in cell bodies (close to the nucleus) and in prolongments of astrocytes without treatment (Fig. 2C). Co-localization with GFAP (Red) confirmed the fact that these cells were astrocytes in both cortex and spinal cord (SC) (Fig. 2D–F”). To be sure that the presence of IgG was not due to recapture from blood during the isolation procedure, we also conducted this analysis on rat DI TNC1 astrocyte cell line. The staining for IgG was also observed which confirmed the ability of astrocytes to produce IgG (Fig. 2G) as neurons [8]. The labeling appeared in granular structures scattered in the cytoplasm suggesting the possibility of being secreted (Fig. 2G). To confirm the ability of astrocytes to produce IgG, we performed pan-transcriptomic and proteomic analyses of the primary cultures and the cell line during normal and after LPS stimulation. After RNAseq analyses, MagicBlast alignment was performed against a specific bank containing all sequences coding for each V (Variable), D (Diversity), J (Junction) domains as well as heavy and light chains referenced in IMGT immunoglobulin database [31] for the *Rattus norvegicus*. It confirmed that reads corresponded to Ig constant part of heavy and light chains. Among them, 63 reads and 3 trinity corresponding to *Igg2b*; 30 reads for *Ighm* and 9 reads and 1 trinity for *IgK* were identified in spinal astrocytes (Supplementary Data 1). Only 2 reads for *Igg2b*, 26 reads, and 1 trinity of *Ighm*, 6 reads and 1 trinity for *Igg2a* and 4 reads and 1 trinity for *IgK* were found in astrocytes from the cortex (Supplementary Data 1). These data are in line with the results of RNAseq performed on astrocytes derived from NPCs of healthy children, brain of healthy rodents as well as the optic nerve of healthy and EAE mice. Indeed, the survey of the Geo DataSets repository revealed the expression in Human astrocytes of *IgL* gene and in rodents’ astrocytes of *Ighm*, *Igg2*, *Igg1*, *IgA* and *IgK* genes (Supplementary Data 1 bis). We then validated our results by RT-PCR performed on cDNA from primary culture of cortex or spinal astrocytes as well as DI TNC1 cell line (Fig. 2H). To mimic an inflammation as observed during the time course of SCI [23, 26], the cells were stimulated with LPS and for 24 h or 48 h with a cocktail of cytokines (Fig. 2H). cDNA from the spleen served as a positive control. The primers used were deduced from peptides identified by proteome analysis after purification processes using Ig enrichment procedure [8]. For IgG2B, these peptides were VDKKVERRNGGIGHKCPTCPTCHK, ALPSPIEKTISKPKGLVR, KNTEPVMDSDGSFFMYSKLNVERSR (Supplementary Fig. 2). Some of these peptides were previously identified in SCI [8]. Another round of RT-PCR allowed the amplification of the complete IgG2B constant heavy chain. The size at 1001 bp corresponded to the mRNA that did not contain the sequence coding for the transmembrane (TM1) or (TM2) domains (Fig. 2H) while the fragments at 1,209 bp exhibited TM1 and TM2 coding sequences (Fig. 2I and Supplementary Fig. 2). These results suggest that a transmembrane form of IgG2B is produced in astrocytes. We then performed a 5’-RACE-PCR to identify the complete transcript (Fig. 2J). Surprisingly, no VDJ coding sequences associated with the constant heavy chain cDNA were identified. However, bioinformatic analyses revealed the presence of a Kozak sequence directly before Exon1 flanking the IgG2B constant chain coding sequence (Fig. 2J, K). Constructs were performed, including this Kozak sequence and the sequence coding for the IgG2B transmembrane form followed by the Flag tag sequence (Fig. 2L). Moreover, the transmembrane domain associated with Kozak was also overexpressed (Fig. 2L’). These constructs were overexpressed in DI TNC1 cells and Western blot analyses confirmed the expression of the construct at the predicted size of 50 kDa at 24 h and slightly at 48 h and 72 h after transfection for the first construct (Fig. 2L) and for the second construct, we observed a specific band at 15 kDa corresponding to the transmembrane domain after 24 h (mostly), 48 h, and 72 h of overexpression (Fig. 2L’). Nevertheless, the second band at 260 kDa was also detected in the same conditions. None of the specific bands were detected after transfection of the empty vector (EV), showing the specificity of the detection. These results confirmed support that IgG2B was synthesized as a free constant heavy chain. Even if no VDJ sequences associated with IgG2B coding sequence were detected, we previously discovered by proteomic analysis alternative proteins corresponding to free variable heavy chain and Kappa light chains (Data not shown). Thus, we investigated our RNAseq data to identify these free variable chains. Several of them were detected (IGVH1-31*01, IGHV5-43*01, IGVH7-16*01) and some were confirmed by RT-PCR performed on cDNA from rat primary cortex and spinal astrocytes (Fig. 3A and Supplementary Fig. 3). Interestingly, no stop codon in the 3’ end (IgHV5-43.01 IgHV1.31.01 or IGVH7-16*01) was found. Moreover, IGVH7-16*01 exhibits an RSS sequence at its 3’ end meaning that it is a free variable chain (Fig. 3A). From previous proteomic analyses, several peptides were identified and covered parts of the IGVHs. RT-PCR that was performed against the sequences coding these variable parts validate their expression (Supplementary Fig. 3A). By contrast, using a forward primer directed against the sequences coding these variable parts and an antisense primer directed against the IgG2B encoding sequence, no amplification was observed. Therefore, these variable parts are not linked to IgG2B heavy chain. However, we cannot conclude whether they are associated with another isotype or if they are free chains.

We then investigated if free variable light chains were also produced. We previously established their existence by discovering from long coding RNA, two variable Kappa chains [9]. We thus also investigated their presence from classical mRNA. We retrieved from our RNAseq data, several free variable kappa light chains with a coding leader sequence in the 5’ position (IGKV3S9*01, IGKV19S1*0) (Fig. 3B and Supplementary Data 1). At the genomic level, an intronic sequence separates the leader sequence (L1) and (L2) attached to the exon coding the variable heavy chain. Their association occurs during pre-mRNA splicing. Thus, its presence in our sequence without any intronic sequence demonstrates that the variable sequences identified are a true transcript and is not linked to genomic amplification. Moreover, IGKV8S6*01 presents a RSS sequence in 3’ position (Fig. 3B and Supplementary Data 1). We also amplified by RT-PCR the complete sequence coding the constant Kappa chain from DI TNC1 cells treated or not with LPS or a cytokine mix, as well as in rat primary spinal and cortex astrocytes (Fig. 3Ca). We then conducted 5’ RACE-PCR on a cDNA library from DI TNC1 cells (Fig. 3Cb). This revealed the presence of a 5’ RSS sequence followed by a joining sequence associated directly with the *Igk* sequence (Fig. 3Cc). Thus, the complete schematic representation of astrocytic IgG2B is presented in Fig. 3C’. IgK were identified in rat primary spinal and cortex astrocytes, and proteomic analyses confirmed their translation (Supplementary Fig. 4A). On the contrary, the Lambda chain encoding sequence was only amplified in rat primary cortex and DI TNC1 astrocytes (Fig. 3Da, Db). Furthermore, from our RNAseq database and RT-PCR analyses, we also identified IgM encoding sequence in rat primary spinal and cortex astrocytes stimulated by LPS (Fig. 3E and Supplementary Fig. 4B). In astrocytes from adult rats, we also identified the sequences coding IgG2B and a variable IgVH chain corresponding to IGVH7-16*01, found in astrocytes isolated from pups (Supplementary Fig. 4Ca, Cb). In primary astrocytes from Human embryos, we identified transcripts related to IgG1 isotype, and heavy constant and light chains (Supplementary Fig. 4Cc). Interestingly, our previous results on the alt IgKV, a free variable kappa light chain named Heimdall protein, established that the chains were associated, and formed aberrant antibodies as observed in cancer cells [32].

### Recombination enzymatic complex

**Fig. 4:**
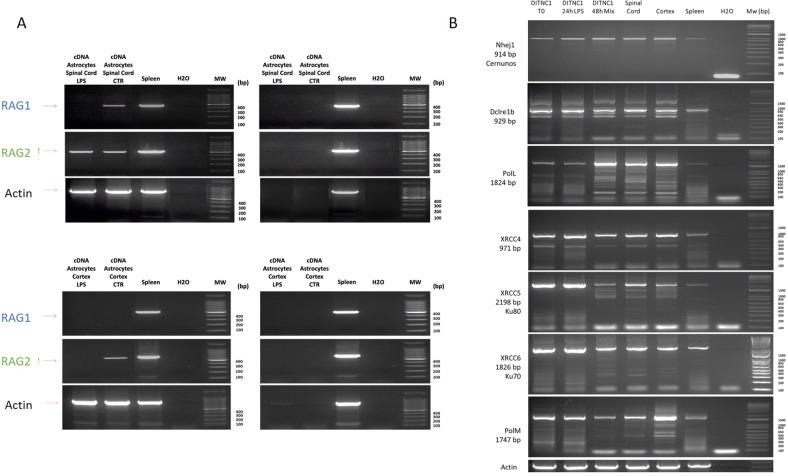
RT-PCR amplification of the transcripts coding the enzymes of the enzymatic complex involved in V(D)J recombination. The experiments were performed on cDNA isolated from rat primary cortex and spinal astrocytes stimulated or not with 200 ng/mL of LPS as well as from DI TNC1 cells stimulated or not with 200 ng/mL of LPS during 24 h or a mix of cytokines for 24 h and 48 h. cDNA from spleen served as a positive control. The experiment was also performed on a negative reverse transcriptase sample to rule out genomic DNA contamination. H_2_O: negative control. **A** RT-PCR amplification of the mRNA coding RAG1 and RAG2. **B** RT-PCR amplification of the mRNA coding Cemunnos-XLF, DCLRE1B, PolL, XRCC4, XRCC5, XRCC6 and PolM.

Even though we only detected aberrant Ig, we investigated whether astrocytes exhibit the classic enzymatic machinery involved in V(D)J recombination. Indeed, in the first step, the two transposases RAG1 and RAG2 will bind to the recombination signal sequences (RSS) 12–23 that flank the selected V, D, or J genes to cleave the DNA. Then, to initiate the repair process, KU70 (XRCC5) and KU80 (XRCC6) will form hairpins and break the ends of these RSS segments. Then, DNA-dependent protein kinase (DNA-PKCs) and Artemis endonuclease (DCLRE1C) are recruited to open these hairpins and allow Terminal deoxynucleotidyl transferase (Tdt, or new nomenclature DNTT) to add nucleotides randomly. This nucleotide addition adds more diversity to antibody synthesis, called junctional diversity. Finally, DNA Ligase IV (LIG4), XRCC4, and Cemunnos-XLF (NHEJ1) link the two segments together [33]. RT-PCR experiments followed by sequencing allowed the identification of all the sequences coding these enzymes (Fig. 4). This was confirmed at the protein level by proteomic analyses performed on primary astrocyte cultures (Supplementary Data 3). The fact that few variable light and heavy chains were identified questions the role of this machinery in pups astrocytes used in this study i.e. V(D)J recombination or DNA repair. However, it cannot be excluded that this machinery is expressed for IgG diversity at a later developmental stage of astrocytes like in lymphopoiesis of B cells.

### The Function of IgG2B constant heavy chain

**Fig. 5:**
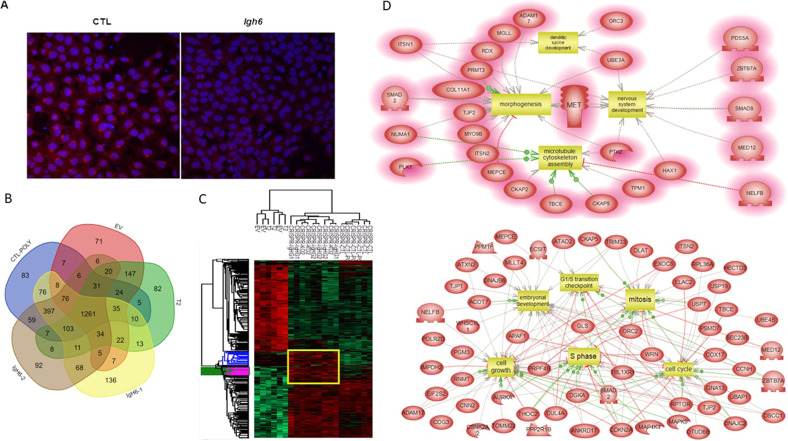
Proteomic study of the effect of rat *Igh6* KO in DI TNC1 astrocyte cell line. **A** Immunofluorescence with anti-IgG carried out on control and *Igh6*-2 KO DI TNC1 cells. **B** Venn diagram of the specific proteins identified in DI TNC1 cells after *Igh6*-1 KO, *Igh6*-2 KO, infection with an empty vector (EV), control cells treated or not with polybrene and *Trop2* KO as a non-target control by Shotgun analyses (*n* = 3). **C** Heatmap representative of the proteins displaying a variation of abundance in DI TNC1 cells after I*gh6*-1 KO, *Igh6*-2 KO, *Heimdall* KO (KO2), control cells treated or not with polybrene, infection with EV and *Trop2* KO as a non-target control, (*n* = 3). **D** Systemic biology representative of the specific cluster common to *Igh6*-1 KO, *Igh6*-2 KO, and *Heimdall* KO (KO2).

We then aimed to investigate the function of IgG2B constant heavy chain. For this purpose, a CRISPR-Cas9 invalidation of IgG2B encoding gene was performed on the rat astrocyte DI TNC1 cell line. This invalidation was performed on two regions of the IgG2B coding sequence using two different sgRNAs. They will be referred to as *Igh6*-1 and *Igh6*-2. As a first control, we performed an immunofluorescence experiment on control and *Igh6*-2 as an example (Fig. 5A). We again detected IgG close to the nucleus whereas the staining disappeared in KO cells. Next, to perform a proteomic study, we added various controls as follows: uninfected cells as well as cells infected with an empty vector or a sgRNA directed against a non-target sequence *Trop-2* (T2) known to not be expressed in astrocytes. We performed a comparison with our previous CRISPR-Cas9 experiment targeting the sequence coding for a free kappa variable light chain named Heimdall (KO2) (data not shown). After invalidation, the cells were harvested, and the proteins were extracted for shotgun proteomics. For *Igh6*-1 KO cells, 71 exclusive proteins were found whereas 73 proteins were exclusively identified in *Igh6*-2 KO cells (Fig. 5B and Supplementary Data 4). Interestingly, among the proteins identified in *Igh6*-1 KO cells, we found Yap1, Tead3, Mcc, Kmt2d, and Ddhd2, which are known to be associated with neural progenitor cells [34] (Supplementary Fig. 5A). We also characterized proteins related to hematopoietic cells (FRG1, Pir), apoptosis regulations (Casp3, Cdk11b, Bax) or neuritogenesis (Plxna1, Rock1, Magi3, Tbl1x). In *Igh6*-2 KO cells, we identified proteins known as polarizing players in brain development (LLGL1, Atxn2) [35, 36] or related to neural stem cells (Gpc4, MAPK). We also identified Akt2, known to be implicated in survival growth and cell proliferation like Pfdn1. Moreover, 30 of the proteins identified were found in organelles and 28 were membrane-bound organelle proteins (Supplementary Fig. 5B). After shotgun proteomics, we applied an ANOVA test with a significance threshold of *p* < 0.01. A heatmap depicting the proteins showing a significant difference in LFQ expression between the different conditions was created. One main cluster was identified with *Igh6*-1 KO and *Igh6*-2 KO compared to control conditions (empty vector (EV), *Trop2* KO, control cells with or without polybrene treatment). (Of note, *Heimdall* KO (KO2) colocalized with *Igh6* KO). This suggests that this free kappa variable light chain and free IgG2B heavy chain control the same biological processes (Fig. 5C and Supplementary Data 5). This cluster representing the over abundant proteins in *Igh6* and *Heimdall* KO conditions encompassed proteins involved in transcription regulation of TP53, metabolism, cell cycle, neuronal system, and developmental biology. Moreover, Reactome analyses established the involvement of these proteins in transcription, oncogenesis, cell cycle and stress response. Taken together, functional enrichment analyses of the specific clusters found for *Igh6* KO followed by Elsevier’s Pathway Studio revealed that most of the proteins are linked to cell proliferation, cell cycle, cell growth, associated with BMP receptor signaling (including Smad2, Smad9), morphogenesis and embryonic development related to neuronal stem cells progenitors (Fig. 5D). As a first validation, we performed two western blots (Supplementary Data 5 bis) to detect Notch2, an astrocytic lineage marker [37] and TGF-B III known to specify differentiation of midbrain progenitors toward neuronal fate and dopaminergic phenotype [38]. Interestingly, we detected transmembrane/intracellular region (NTM) and intracellular region (NCID) of Notch2 in control conditions (Ctrl, EV, and T2), whereas it disappeared after *Igh6* KO. By contrast, TGF-beta III only appeared after KO. Taken together, IgG2B could regulate astrocytes at the level of the cell cycle, transcription, and to maintain the phenotype by regulating transcription factors involved in neuronal cell reprogramming and growth. This suggests that when *Igh6* is KO then astrocytes could be reprogrammed to switch in neurons as we previously demonstrated for the free variable kappa light chain, Heimdall [9].

### IgG2B overexpression impact on DI TNC1 cells

Two constructions have been realized. The first one, already described in (Fig. 2K), consisted of the Kozak sequence followed by the *Igh6* and its Transmembrane domain with a Flag tag at the end of the construct (Supplementary Fig. 6A). The second construct is the same as the previous one without the TM domain and the third one, consisted only of the Kozak sequence and the TM domain with the flag sequence. The Western blot performed in non-reducing conditions revealed for the first construction presence of a specific band near a size of 260 kDa after 24 h mostly and 48 h of overexpression (Supplementary Fig. 6A). Taken together, it seems that the IgG2B chain could associate with itself but also with the light chain (IgKV and IgGV) as we already demonstrated in anti-IgKV western blot performed in reducing conditions on LPS-stimulated extracts from primary cultures of spinal astrocytes stimulated with LPS (Supplementary Fig. 6B). Moreover, Western blot analyses in reducing conditions conducted with anti-IgKV on secretomes of LPS-stimulated *Igh6* KO cells compared to controls (T2, EV and CTR) cells, showed the lack of the band at 60 KDa in *Igh6* KO cells (Supplementary Fig. 6C). Furthermore, shotgun proteomic analyses of the cell extract from the 3 different constructs compared to control and EVs showed differential specific proteomic representation illustrated in the Venn diagram schematic (Supplementary Data 6 and Supplementary Fig. 6D). Forty-six common proteins for the three conditions have been identified compared to EV and control and 41 between the two first conditions. Among these identified proteins, the Yap1 transcription factor is known to be implicated in astrocytic proliferation and is known to promote astrocytoma [39]. Similarly, enhanced activity of Crtc1 is implicated in cancers [40]. Moreover, the presence of the immunoglobulin superfamily (embigin) and the immunity-related GTPase family reinforced the inflammatory and proliferative profile of astrocytes. Altogether, overexpression of IgG2B constant chain seems to reinforce the astrocyte fate but also switches it towards proliferation and astrocytoma.

#### IgG2B interactors identified in proximal interactome uncovers CD98hc-like features

**Fig. 6:**
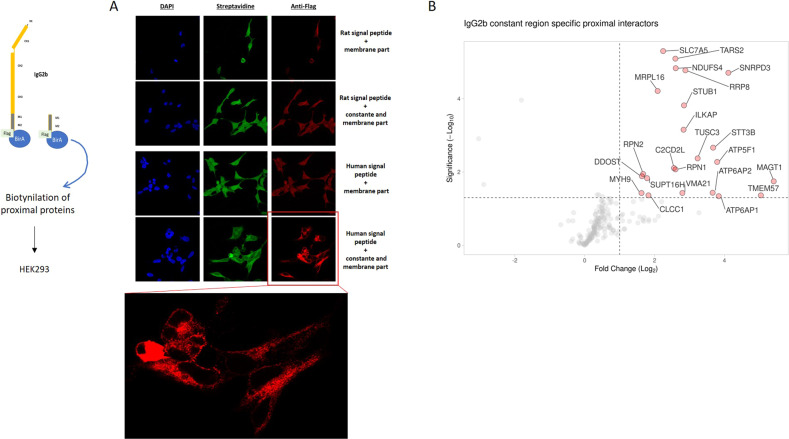
Proximal labeling study in HEK293 cells with rat IgG2B transmembrane form as a bait. **A** Detection of biotinylated proteins (green, Streptavidin Alexa Fluor 488 conjugate) and rat IgG2B (red, anti-Flag) in HEK293 cells after overexpression of IgG2B in fusion with BirA*Flag. IgG2B was overexpressed with a rat or a human signal peptide. Nucleis were stained with DAPI (blue). A control consisting of the overexpression of the transmembrane domain itself fused to a rat or a human signal peptide has been included in the experiment. The inset represents a zoom of the results observed after detection of IgG2B with anti-Flag. **B** Systemic biology representative of proximal interactome of rat IgG2B transmembrane form.

To get insight in IgG2B intracellular protein network, we performed a series of proximity-based biotin labeling experiments (Fig. 6A). These experiments were designed to identify the intracellular proteins at the proximity of IgG2B in a constant heavy chain domain-dependent manner. We performed a series of BirA* alone controls and expressed the signal peptide (SP) fused to the IgG2B transmembrane (TM) domain with a C-terminal BirA*Flag tag (BF) to remove the BioID basal signal and the membrane protein processing-specific backgrounds, respectively. Both the SP-TM-BF and SP-Constant-TM-BF identified Signal Recognition Particle (SRP) receptor (SRPRB, SRPR, SRP14) and 32/159 structural constituent of the ribosome, supporting correct recognition of the SP and proper processing through the ER (Fig. 6B). Comparing the SP-Constant IgG2B domain (C)-TM-BF data to both BirA* alone and SP-TM-BF controls, we identified 23 proximal high-confidence SP-C-TM-BF interactors (i.e. proteins brought to SP-C-TM-BF through the constant domain). Amongst those, we identified 6 components of the oligosaccharyltransferase complex (DDOST, MAGT1, RPN1, RPN2, STT3B and TUSC3) which suggests that SP-C-TM-BF is processed through the N-linked glycosylation pathway. Of note, SP-C-TM-BF harbors a single N-X-S/T glycosylation sequon located at the N183 (-S–T). We also identified three components of the proton-transporting two-sector ATPase complex (ATP5F1, ATP6AP1 and ATP6AP2) which catalyzes the synthesis or hydrolysis of ATP coupled to an H + transport across membranes. Together, these proximal interactions strongly suggest a plasma, lysosomal or vesicular membrane addressing of SP-C-TM-BF. In line with this hypothesis, we detected TMEM57 (Macoilin), C2CD2L, ILKAP, and SLC7A5 among the remaining 14 high-confidence interactors (Supplementary Data 7). TMEM57 association is strongly dependent of the IgG2B constant domain. It is a poorly characterized protein which has solely been reported to regulate neuronal activity, resulting in Ca^2+^ imbalance [41, 42]. Of peculiar interest, the phospholipid transfer protein C2CD2L ensures the transit of phosphatidylinositol from ER to the plasma membrane in a Ca^2+^-dependent manner [43]. We hypothesize that the interactions between IgG2B, TMEM57 and C2CD2L may occur at the tethering sites between ER and plasma membrane and could link membrane-embedded IgG2B to neuronal physiology through PIP and Ca^2+^ regulation. The large neutral amino acids transporter small subunit 1 (SLC7A5, LAT1) appears as another partner of membrane-embedded IgG2B. LAT1 is crucial in transporting large neutral amino acids as well as drugs or metabolites (e.g. L-DOPA [44] across the blood–brain barrier (BBB)). Beyond its function, SLC7A5 acts through heterodimerizing with SLC3A2 (CD98 heavy chain; CD98hc). This is interesting regarding our study since CD98hc is a N-glycosylated protein [45] and SLC7A5 (LAT1) is one of its associated light chain ensuring amino acids transport. These data suggest that membrane-embedded IgG2B could substitute SLC3A2 to form a membrane heterodimer with SLC7A5. In addition, the specific interaction with ILKAP (integrin-linked kinase-associated phosphatase) potentially indicates a link between IgG2B and the PI3K/AKT pathway through its reported ability to dephosphorylate GSK3 [46]. This pathway is downstream the canonical CD98hc-LAT1 complex. Our data thus sketch an alternative CD98 receptor where IgG2B could replace CD98hc and associate with LAT1 to form a functional entity potentially impacting multiple pathways, e.g., PI3K/AKT, Wnt (through modulating GSK3b activity, thus β-catenin regulation) and mTORC1 (because of its amino acids transporter activity when associated with LAT1). Of note, we did not detect integrins as IgG2B interactors, suggesting a partial recapitulation of CD98 organization.

Together, our proximal interactomics study suggests that IgG2B could be: (i) N-glycosylated by the OST complex; (ii) addressed at the vesicle membrane through ATPase complex-containing vesicles; and (iii) partially behaves as CD98hc through its association with LAT1 and ability to recruit ILKAP which potentially regulates downstream signaling pathways. These experiments were performed in HEK293 and certainly present cell-type-dependent limitations. However, LAT1 is expressed in astrocytes, and we believe that this dataset uncovers a putative CD98hc-like function which represents a great interest for pharmaceutical research (because of the ability of CD98 to ensure BBB large molecule crossing). Further experiments are needed to characterize IgG2B-LAT1 interaction in astrocytes and could represent an interesting path for astrocytic neoplasms understanding [47]. Furthermore, it will be of peculiar interest to investigate whether the IgG2B-LAT1 proximal interaction occurs concomitantly with TMEM57 and C2CD2L, at the core of a potentially novel cell signaling platform.

### Antigen recognition by astrocytes Ig

**Fig. 7:**
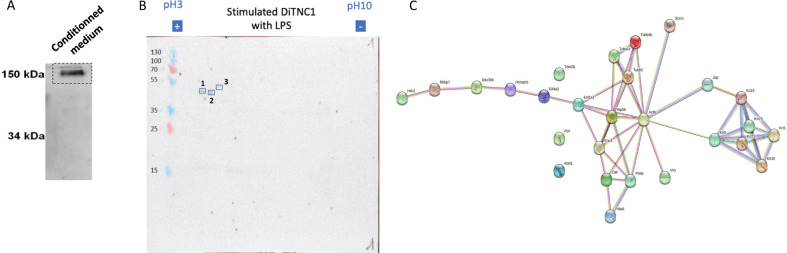
Identification of neural immunoglobulin targets. **A** Detection of a complete secreted IgG2B by western blot in non-denaturing condition performed on secretome of DI TNC1 cells stimulated with 200 ng/mL of LPS. **B** Western blot of 2D gel carried out on protein extracts of DI TNC1 astrocytes incubated with secretome of DI TNC1 astrocytes stimulated during 24 h with 200 ng/mL of LPS. **C** String of proteins identified during western blot performed on 2D gel and related to intermediate filaments of the cytoskeleton.

We established that aberrant Ig have been identified in astrocyte extracts. Their secretion must be demonstrated and their ability to recognize antigens and function as antibodies too. This supports the fact that all different free chains may associate to produce a structure functioning as a complete antibody (Fig. 7A). To evaluate whether this IgG antibody can recognize antigens, Western blot of protein extracts from DI TNC1 cells was performed on 2D gels (Fig. 7B). All membranes were incubated with secretome of DI TNC1 cells stimulated with 200 ng/mL of LPS as a source of primary antibodies. Of note, these secretomes were collected after the culture of cells in DMEM without FBS for 24 h. Then secondary antibody was added, and positive spots were detected (Fig. 7B). Each recognized spot was then analyzed by shotgun proteomics. Their identification revealed proteins involved in microtubule cytoskeleton organization i.e. Tubulin alpha chain and beta chains, Vimentin as well as Eif4a2 and Pdia6 (Supplementary Data 8). These two factors are known to be involved in stem cell pluripotency [48] and metastasis [49].

**Fig. 8:**
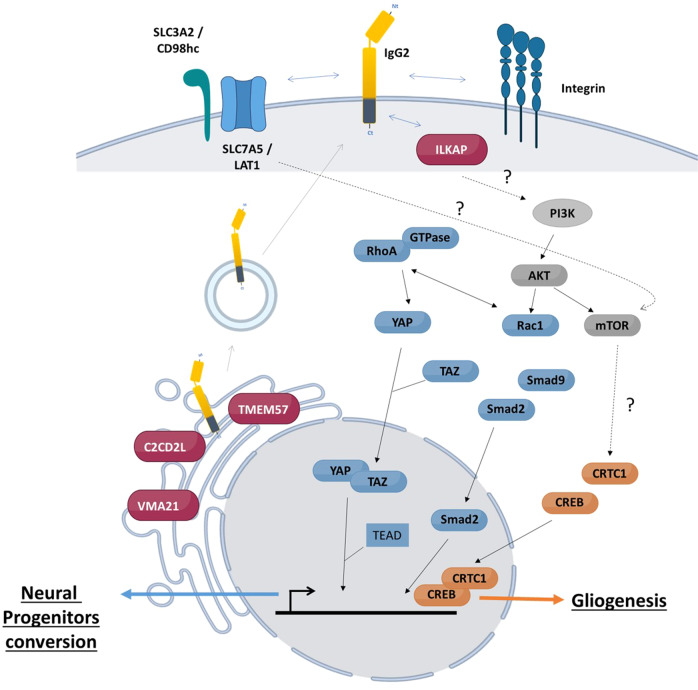
Schematic representation of the potential intracellular role of IgG2B to preservation and conversion of the astrocyte phenotype. IgG2B can interact either with proteins from the endoplasmic reticulum, with LAT1 or integrins at the membrane surface. The blue pathway is found upon CRISPR-Cas9 invalidation of the IgG2B heavy chain while the orange pathway was identified upon overexpression. The proteins in red were demonstrated in proximal interaction with IgG2B. The ones in gray are related to proteins involved in ILKAP and CD98hc/LAT1/SLC7A5 signaling pathway.

Taken together, these results are in line with the CRISPR-Cas9 data. Intermediate filaments (IFs) are key players in the control of cell morphology and structure as well as in active processes such as cell polarization and migration. Previous studies have shown that IFs are major players in fetal brain development. Vimentin was observed in astrocytes and glial progenitor cells/glioblasts while tubulin beta II was only found in some glial progenitor cells/glioblasts and not at all in mature astrocytes [50]. In vitro, astrocytes lacking GFAP or vimentin were shown to be the substrate for increased neuronal plasticity [51]. Thus, regulation of IFs by aberrant IgG chains in astrocytes is reminiscent of what is observed during the control of the astrocyte-to-neuronal progenitor stem cell conversion as presented in (Fig. 8).

## Discussion

In this study, we identified variable and constant heavy and light chain coding sequences in astrocytes. If some are from the CDS, others are considered pseudogenes. However, their translation was demonstrated by proteomic analysis. The use of pseudogenes to synthesize multiple IglV and IghV has already been observed in mice and chickens. Within the multiple IglV and IghV pseudogenes, the number of stop codons contained in the “coding” sequence is far lower than would be expected while nucleotide substitutions occurred at random. In fact, most stop codons introduced by point mutations are “corrected” and eliminated by further point mutations within the same codon [52–54]. In rodent B cells, the conservation of open reading frames plays a role in any putative function of pseudogenes involved in somatic gene arrangement [55] as we showed in astrocytes not only from pups but also from adult rats and human embryos. Similar results were already observed in human immunome. In fact, gene conversion with pseudogenic sequences like somatic hypermutation or class switch conversion or recombination (CSR) of the constant region increases the repertoire for a vast diversity of immunological recognition molecules starting from a limited number of gene segments [56, 57]. This ensures the detection of all possible foreign organisms and substances. Moreover, under inflammatory conditions, our results demonstrated the existence of a specific subpopulation of astrocytes displaying an expression of plasmocyte markers such as CD38 and CD138. This is consistent with their ability to synthesize a diversity of immunoglobulin subtypes such as IgG2B and IghM. We can rely upon these results with those depicting a glia subpopulation in the spleen [58]. Splenic glia differentially expresses genes associated with an immune response such as cytokine–cytokine receptor interactions, phagocytosis, and the complement cascade. It thus can be envisaged that activated astrocytes share common features with this splenic glial population.

In the CNS, astrocytes present multiple important physiological functions such as maintenance of the blood–brain barrier, production of neurotrophic factors, adjustment of the density of ions in extracellular space, modulation of inflammation, regulation of neuronal activity, synaptic transmission, and neural circuit function [59]. Astrocyte proliferation stops after 1 month of age in rodents. However, in response to insults such as traumatic brain injury or spinal cord injury (SCI), astrocytes proliferate [60] to perform astrogliosis [23, 26, 61–64]. Astrocytes and neurons originate from the same progenitor cells. Therefore, astrocytes might have kept intrinsic neuronal conversion potential. Supporting this fact, it has been observed in certain circumstances that astrocytes can dedifferentiate and switch their phenotype to become neuron-like cells [65]. It has even been speculated that glial cells might be a kind of NPC-like cells due to the observations that astrocytes can express certain NPC markers such as Nestin and Sox2, whereas certain glial markers such as GFAP are also observed in NPCs. However, it has recently been shown that only cortical astrocytes but not astrocytes from other brain regions such as the hippocampus and the cerebellum can be converted into neurons under the current condition [66, 67]. Pax6 was found as one of the key transcription factors that can reprogram astrocytes to neurons [68]. Other transcription factors (TFs) such as Ascl1, Lmx1a and Nurr1 (Nr4a2) so-called ALN TFs have also been used to switch astrocytes to dopaminergic neurons. Similarly, the combination of ALN with NG2 glia switches it into GABAergic neurons in mice [59]. These TFs seem to promote chromatin remodeling and activate the TGFβ, Sonic hedgehog (Shh) and Wnt signaling pathways [69]. NeuroD1, a bHLH pro-neural TF essential for embryonic development and adults’ neurogenesis, is also involved with Sox2, Ngn2 and Ascl1 in the conversion of glia into neurons. According to these data, we can speculate that factors implicated downstream of neural TFs regulation of Shh and Wnt signaling pathways can participate in astrocytes to neuron conversion [70]. Accordingly, it has been demonstrated that astrocytes can be converted into neurons by the knockdown of *Ptbp1* [71]. Ptbp1 plays an essential role in pre-mRNA splicing and mediates several cellular processes in certain types of cells, including the activation of immune cells as well as the growth and differentiation of neuronal cells. In such a line, our present study suggests that IgG2B could also play a role as a gatekeeper of astrocytes to neural progenitor conversion.

Indeed, when we Knocked-out the rat *Igh6* gene by CRISPR-Cas9, we demonstrated that TFs such as YAP, TEAD and SMAD2/9 were more abundant and Notch2 forms disappeared [37] whereas TGF-Beta III [38] appeared. It is known that YAP and TEAD gain of function causes marked expansion of the neural progenitor population (Fig. 8). This is partly linked to their ability to promote cell cycle progression by inducing cyclin D1 activity and to inhibit cell differentiation by suppressing NeuroM. Their loss of function results in increased apoptosis, whereas repressing their target genes leads to premature neuronal differentiation [34]. In accordance with these results, an over-proliferation molecular profile of astrocytes was also observed after the KO of *Igh6* gene. Besides, it has previously been demonstrated that these various proteins play a key role during neuronal differentiation by inhibiting the upstream kinases of the Hippo signaling pathway and the input for their conversion to neuronal progenitor stem cells [72] by triggering the TGF-beta/BMP pathways. Moreover, it is well known that Notch2 is an astrocytic lineage marker [37], and TGF-B III specifies the differentiation of midbrain progenitors toward neuronal fate and dopaminergic phenotype [38]. To better define IgG2B activity, we then performed its overexpression in HEK293 cells and study its localization. We observed that it was synthesized as a free transmembrane constant chain through its Kozak sequence located upstream of its first exon and was addressed to vesicular structures such as endosomes or extracellular vesicles. Through proximal BioID studies, we also found that IgG2B could be: (i) N-glycosylated by the OST complex; (ii) addressed at the membrane of ATPase complex-containing vesicles and (iii) partially behaves as CD98hc through its association with LAT1 and its ability to recruit ILKAP, which potentially regulates downstream signaling pathways (Fig. 8). These experiments were performed in HEK293 and certainly present cell-type dependent limitations. However, LAT1 is expressed in astrocytes, and we believe this dataset uncovers a putative CD98hc-like function which represents a great interest for pharmaceutical research since CD98 ensures BBB crossing of large molecules. Further experiments are needed to characterize IgG2B-LAT1 interaction in astrocytes and could represent an interesting path to understand astrocytic neoplasms [47]. During these experiments, we also detected TMEM57 and C2CD2L as putative IgG2B interactors (Fig. 8). It will be of peculiar interest to investigate whether the IgG2B-LAT1 proximal interaction occurs concomitantly with TMEM57 and C2CD2L, at the core of a potentially novel cell signaling platform. Indeed, it is worth noting that TMEM57 (MACO-1) is known in *C. elegans* to be involved in different neuronal functions. It regulates the forgetting of olfactory adaptation to isoamyl alcohol, which is an attractive odorant perceived by different types of sensory neurons [73]. In humans, MACO-1 is expressed in neural progenitors particularly during memory consolidation in the hippocampus [74]. Moreover, it also acts in the ER to regulate the assembly or traffic of ion channels or ion channel regulators to modulate neuronal excitability [75]. Based on these data and our results, it is tempting to speculate that IgG2B acts as a scaffold protein bringing together various regulators involved in the astrocytic phenotype.

In parallel with IgG2B, we also identified free variable heavy chains synthesized without the DJ sequences, free variable kappa light chains as well as a constant kappa light chain linked to a joining sequence. As suggested by our previous Western blot data and proteomic experiments [9], this does not exclude the possibility that these free variable chains may associate with their respective constant chains. The heavy and light chains formed independently may then interact to form a structure related to a BCR expressed at the membrane of endosomes/extravesicular vesicles or to be secreted to recognize some antigens or activate specific FcγR. In such a line, we recently demonstrated that neurons express CD16 and CD32b, which are linked to the antibody-dependent neurite outgrowth modulation responses (A.D.N.M) [8]. Moreover, we demonstrated that these neural immunoglobulins can recognize antigens in astrocytes such as the PRSS1 protein, known to be involved in neuroprogenitor stem cell conversion [76]. This recognition of autoantigens may also constitute another way to stabilize the fate of astrocytes by blocking their conversion to neurons. Taken together, these results strongly suggest that astrocyte immunoglobulins coding by CDS or pseudogenes are aberrant IgG chains maintaining their fate and preventing their conversion to neuronal lineages. Thus, these proteins can clearly be novel therapeutic targets for in vivo direct conversion of astrocytes to neurons in neurodegenerative diseases such as TBI, SCI or stroke incidents.

## Supplementary information

Original Data File

aj-checklist

Table 1

Table 2

Supp Figures Legends

Supp Figure 1

Supp Figure 2

Supp Figure 4

Supp Figure 3

Supp Figure 5

Supp Figure 6

Supp Figure 7

Suppemental Informations data

Supplementary Dataset 1

Supplementary Dataset 1bis

Supplementary Dataset 2

Supplementary Dataset 3

Supplementary Dataset 4

Supplementary Dataset 5

Supplementary Dataset 5bis

Supplementary Dataset 6

Supplementary Dataset 7

Supplementary Dataset 8

## Data Availability

The datasets and the Perseus result files used for analysis were deposited at the ProteomeXchange Consortium (http://proteomecentral.proteomexchange.org) via the PRIDE partner repository with the dataset identifier PXD040562 (User name: reviewer_pxd040562@ebi.ac.uk; password: ptWWnlXS). Genbank accessory numbers: OQ559557 (truncated IgG2B) and OQ559558 (Jexon IgKc). BioSample accessions for RNA-Seq data: SRX19591519, SRX19591520.
