# Supplementary material for: Astrocytes express aberrant immunoglobulins as putative gatekeeper of astrocytes to neuronal progenitor conversion
Source: Cell Death Dis. 2023 Apr 4;14(4):237. doi: 10.1038/s41419-023-05737-9 (PMC10073301; doi:10.1038/s41419-023-05737-9)
Supplement: Supplementary file 1 — Original Data File [file 41419_2023_5737_MOESM1_ESM.pdf]

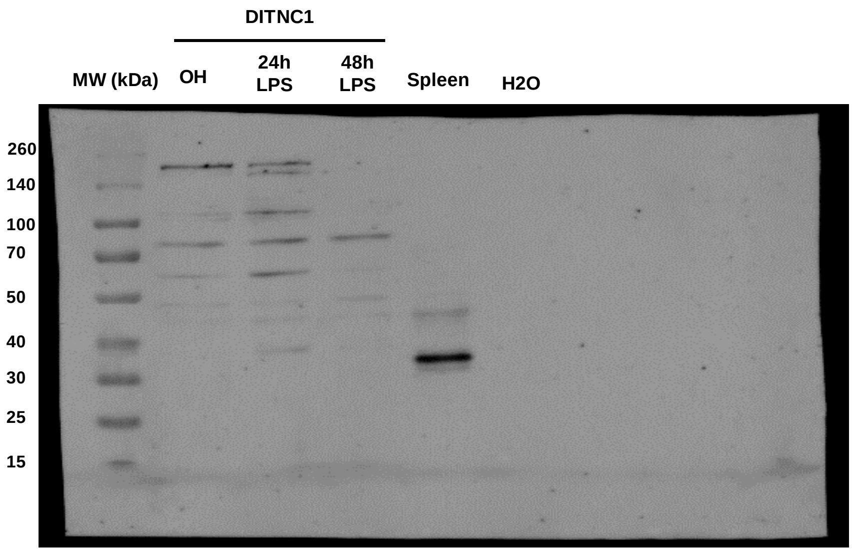

Figure 1 : Validation that primary cultures of the cortex and spinal astrocytes are devoid of B cells. B) Western blot analyses of CD20 in spleen and DI TNC1 cells stimulated or not with 200 ng/mL of LPS during 24h and 48H.

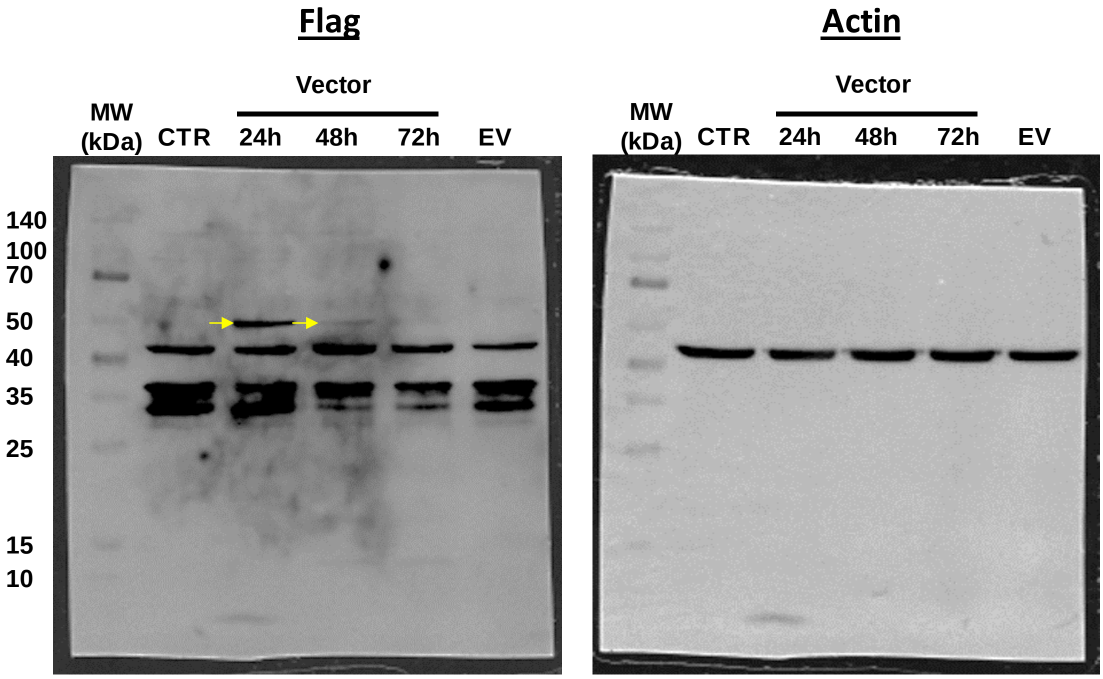

Figure 2 : Identification of immunoglobulin constant heavy chain in astrocytes. L) Overexpression of rat astrocyte IgG2B transmembrane form in fusion with Flag Tag in DI TNC1 cells. Western blot analysis using an antibody directed against the Flag tag was carried out 24h, 48h, or 72h after transfection. Non-transfected cells and transfection with an empty vector served as negative controls.

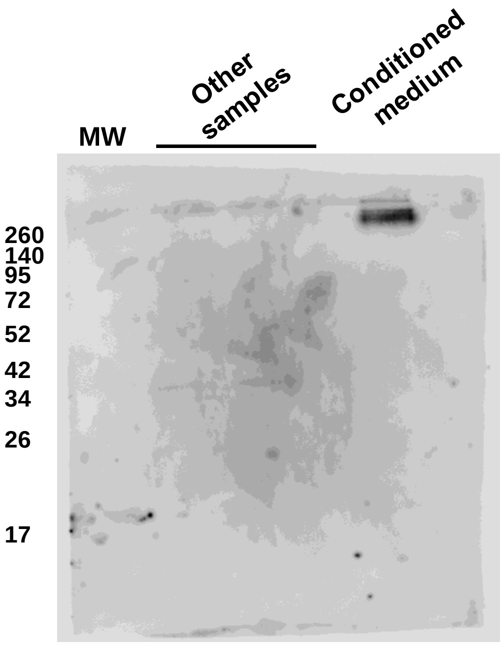

Figure 7: Identification of target neural immunoglobulins. A) Detection of a complete secreted IgG2B by western blot in non-denaturing condition performed on secretome of DI TNC1 cells stimulated with 200 ng/mL of LPS.

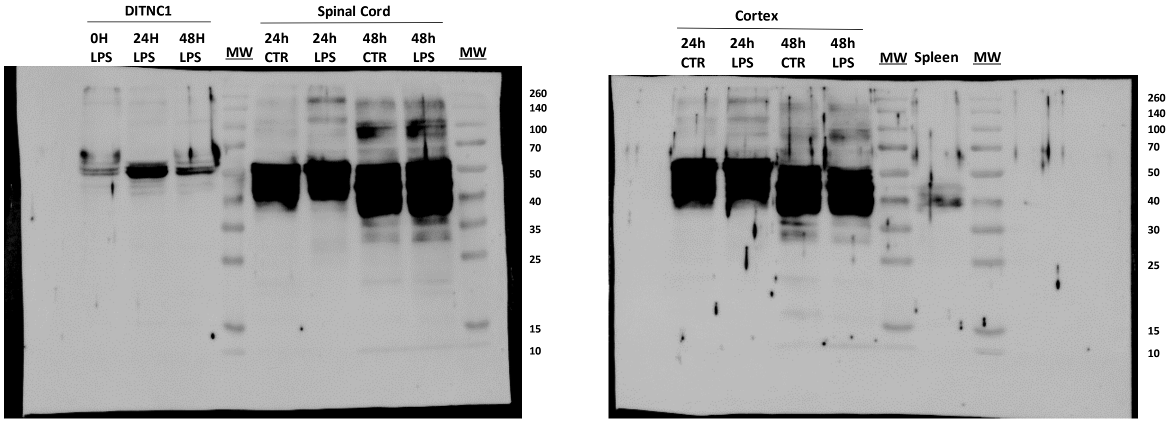

Supp. Figure 1 : B) Western blot analyses of GFAP in DI TNC1 cells and rat primary cortex and spinal astrocytes stimulated or not with 200 ng/mL of LPS during 24h or 48H as well as in spleen.

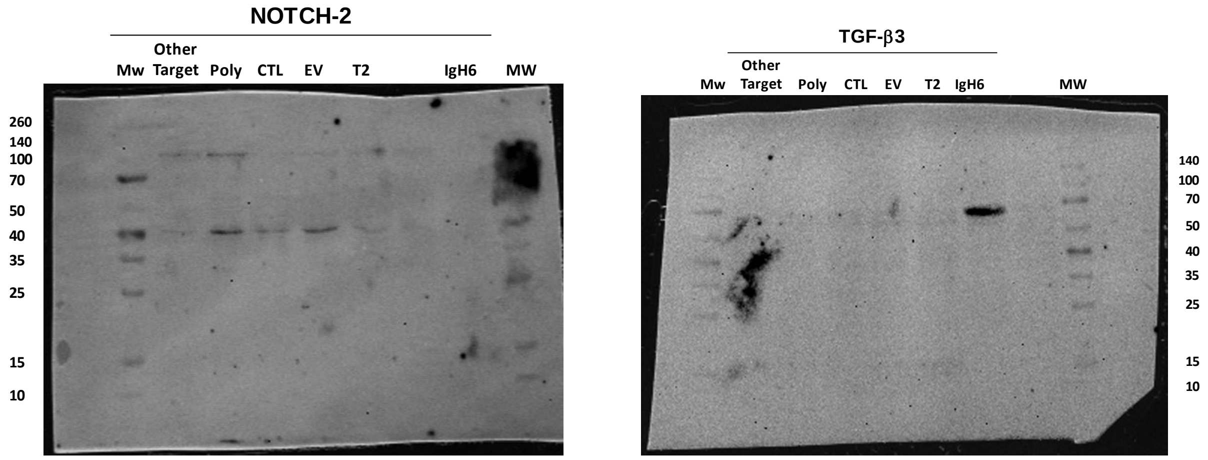

Supp data 5 bis : Detection of Notch 2 transmembrane/intracellular region (NTM) and intracellular region (NCID) forms as well as TGF-beta III by western blot. The experiments were performed on protein extracts from DI TNC1 cells after *IgH6* KO, control cells, infection with empty vector (EV) and *Trop2* KO as a non-target control. Poly = Polybrene.

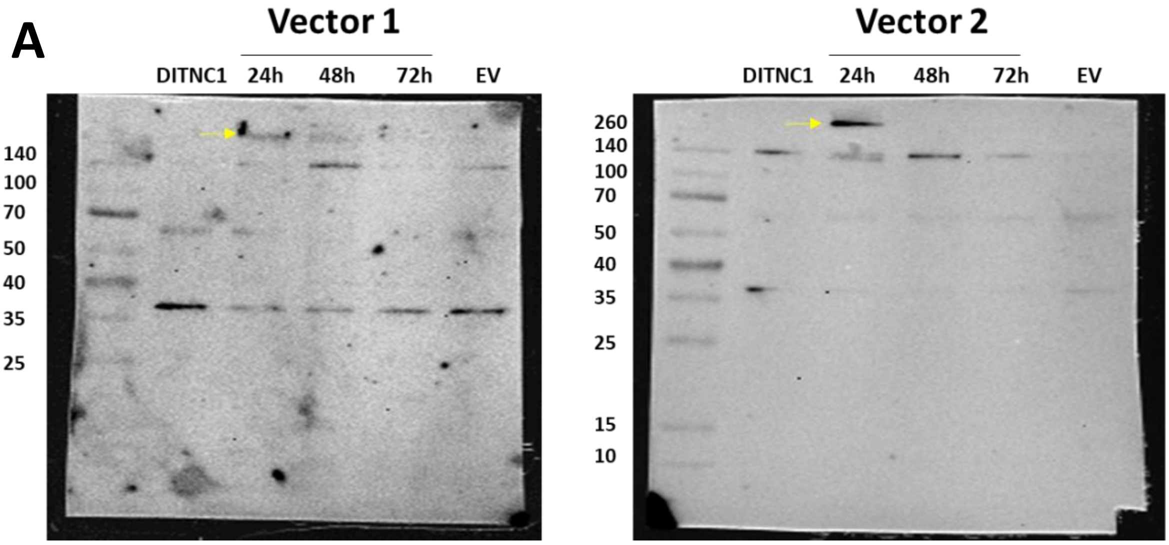

B

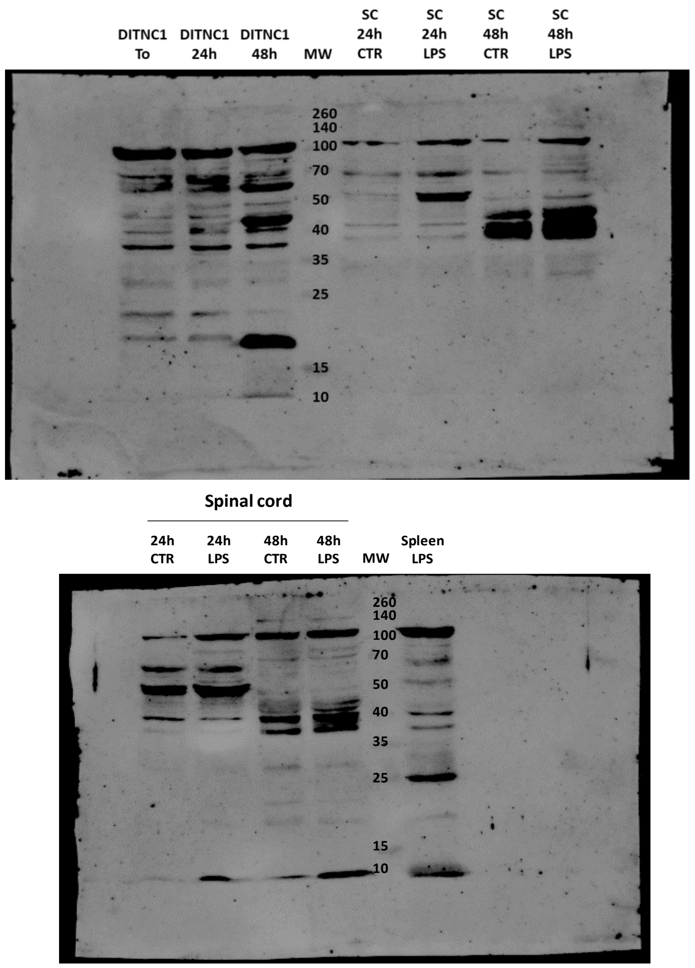

C

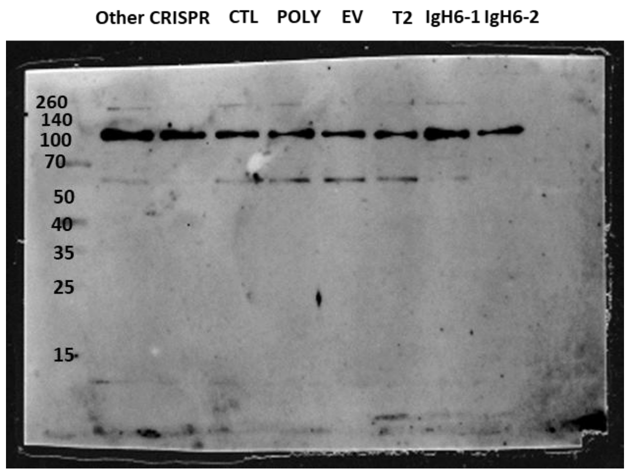

Supp. Figure 6: A) Anti-Flag Western blot analyses from Gel electrophoresis in non-reducing conditions of IgGH constructs with or without TM domain in DI TNC1, cells transduced with empty vector (EV) or LPS-stimulated for 24h, 48h or 72h. **B)** anti-IgKV Western blot analyses under reducing conditions of DI TNC1 cells as well as spinal and cortex primary astrocytes stimulated or not with LPS, **C)** Western blot analyses of secretome of LPS-stimulated CRIPSR-CAS 9 (IgH6-1, IgH6-2) DI TNC1 cells versus T2 (Trop2), control, polybrene and empty vector transfected cells with anti-Heimdall.
