# Supplementary figures and images for: Astrocytes express aberrant immunoglobulins as putative gatekeeper of astrocytes to neuronal progenitor conversion
Source: Cell Death Dis. 2023 Apr 4;14(4):237. doi: 10.1038/s41419-023-05737-9 (PMC10073301; doi:10.1038/s41419-023-05737-9)

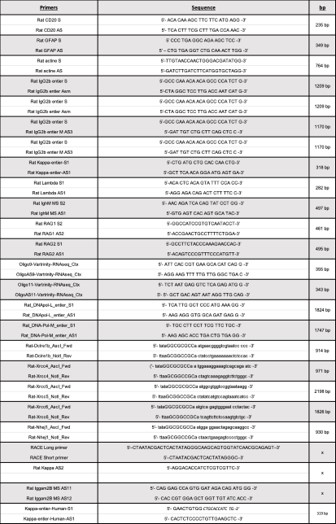

Supplement: Supplementary file 3 — Table 1 [file 41419_2023_5737_MOESM3_ESM.jpg]

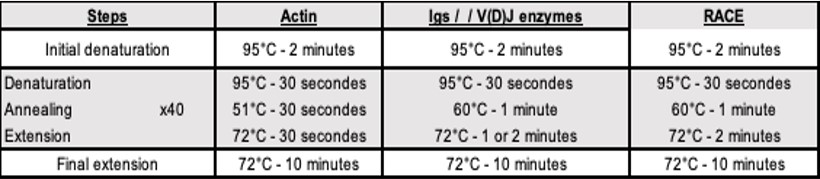

Supplement: Supplementary file 4 — Table 2 [file 41419_2023_5737_MOESM4_ESM.jpg]

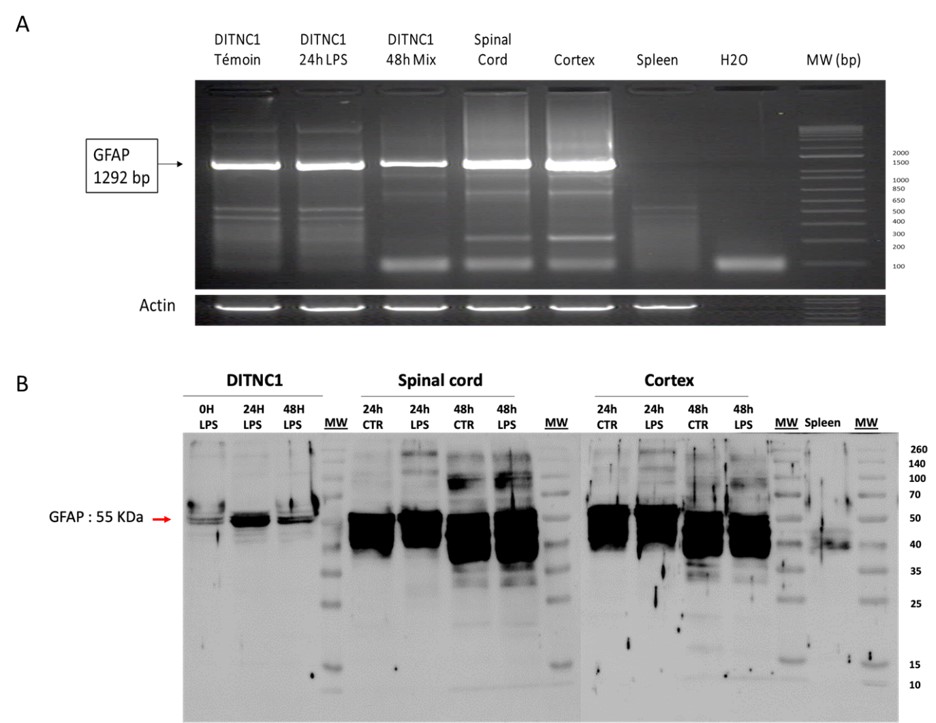

Supplement: Supplementary file 6 — Supp Figure 1 [file 41419_2023_5737_MOESM6_ESM.jpg]

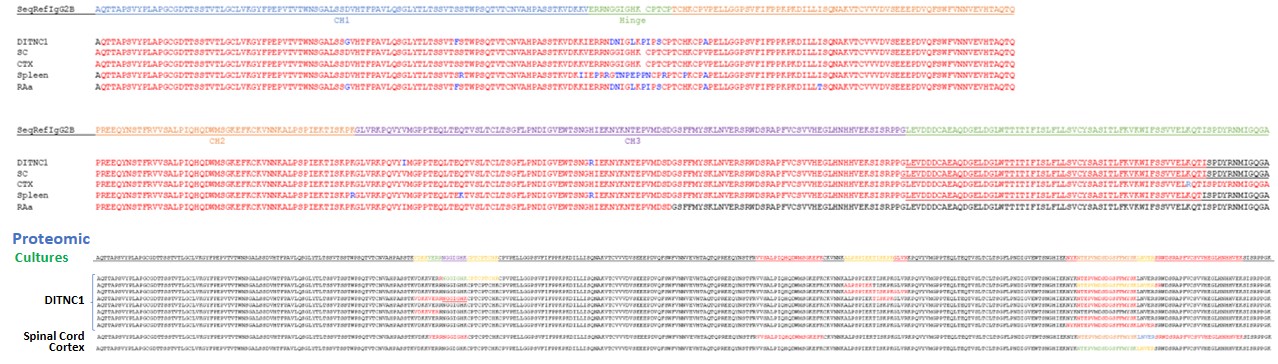

Supplement: Supplementary file 7 — Supp Figure 2 [file 41419_2023_5737_MOESM7_ESM.jpg]

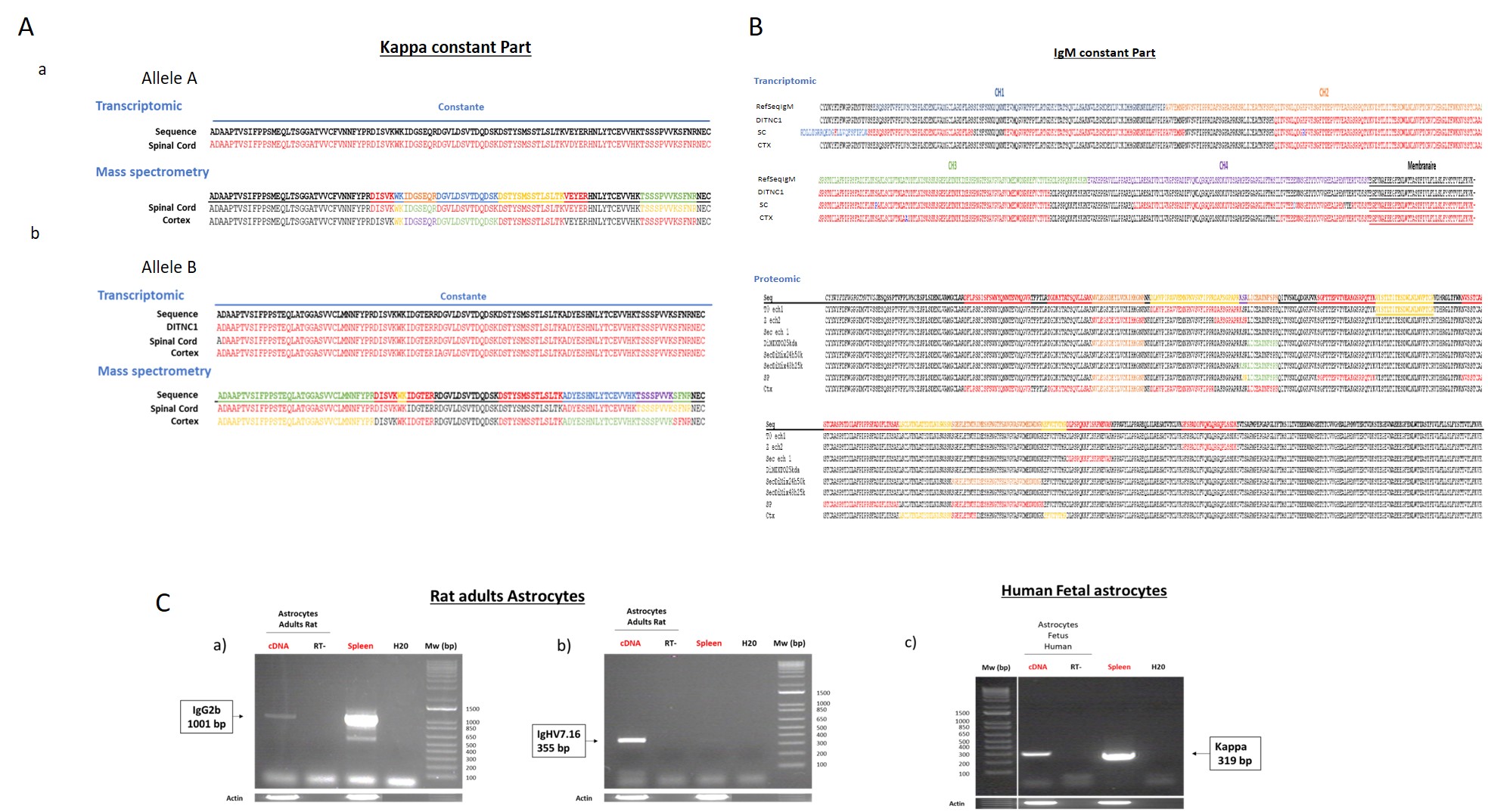

Supplement: Supplementary file 8 — Supp Figure 4 [file 41419_2023_5737_MOESM8_ESM.jpg]

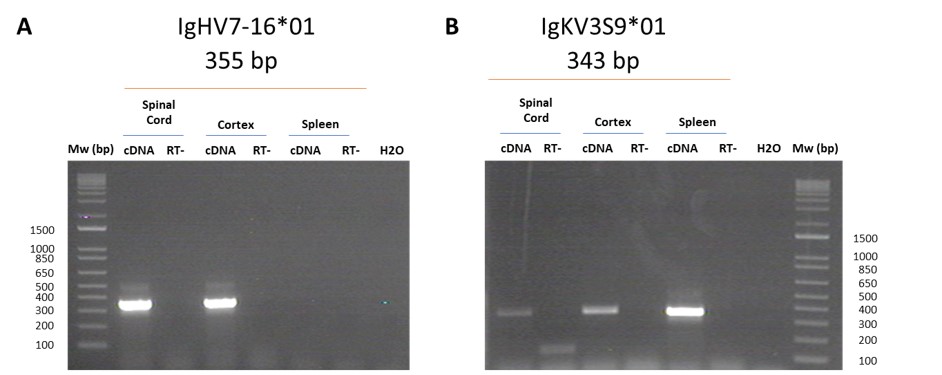

Supplement: Supplementary file 9 — Supp Figure 3 [file 41419_2023_5737_MOESM9_ESM.jpg]

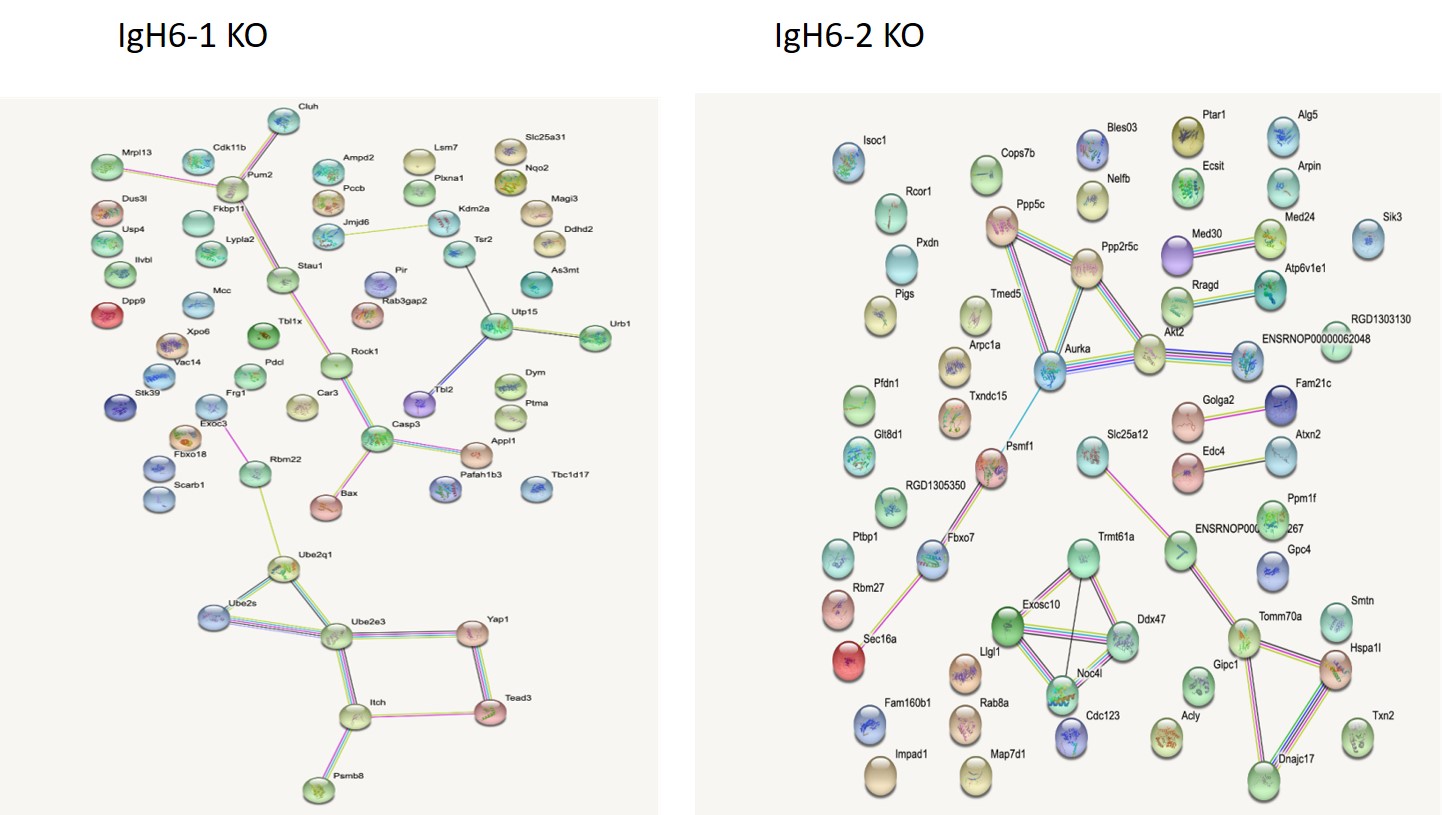

Supplement: Supplementary file 10 — Supp Figure 5 [file 41419_2023_5737_MOESM10_ESM.jpg]

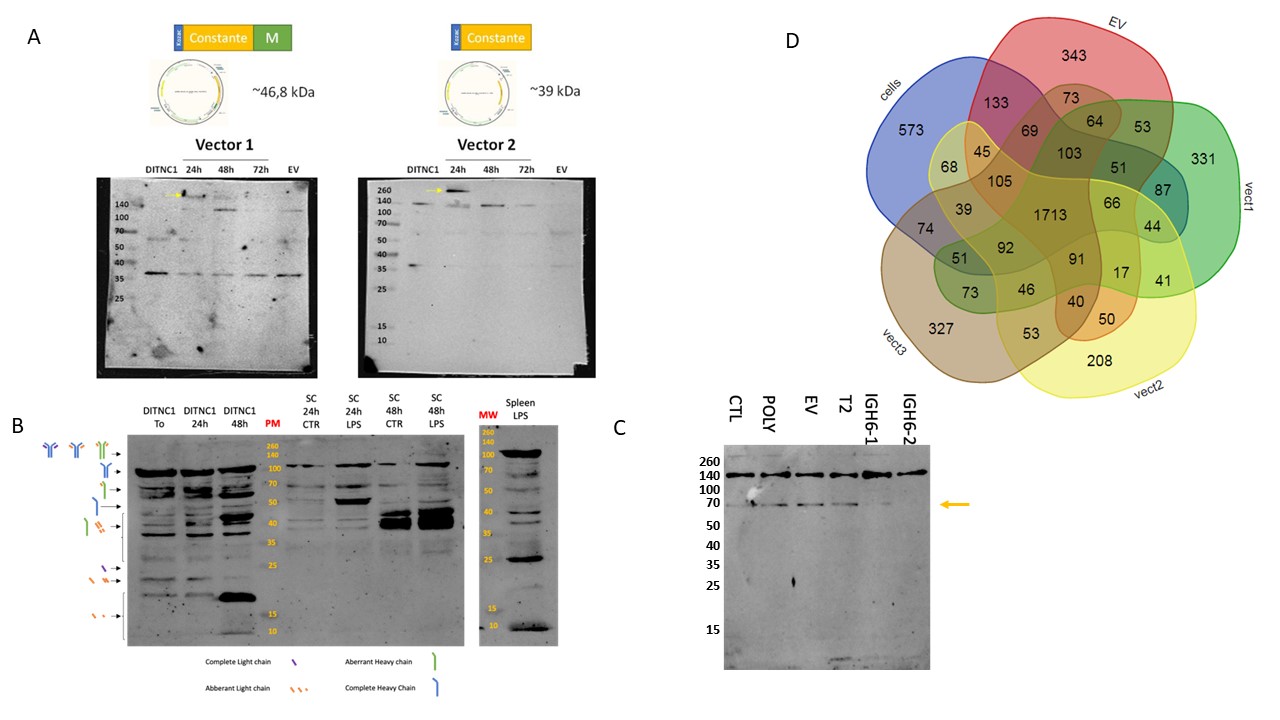

Supplement: Supplementary file 11 — Supp Figure 6 [file 41419_2023_5737_MOESM11_ESM.jpg]

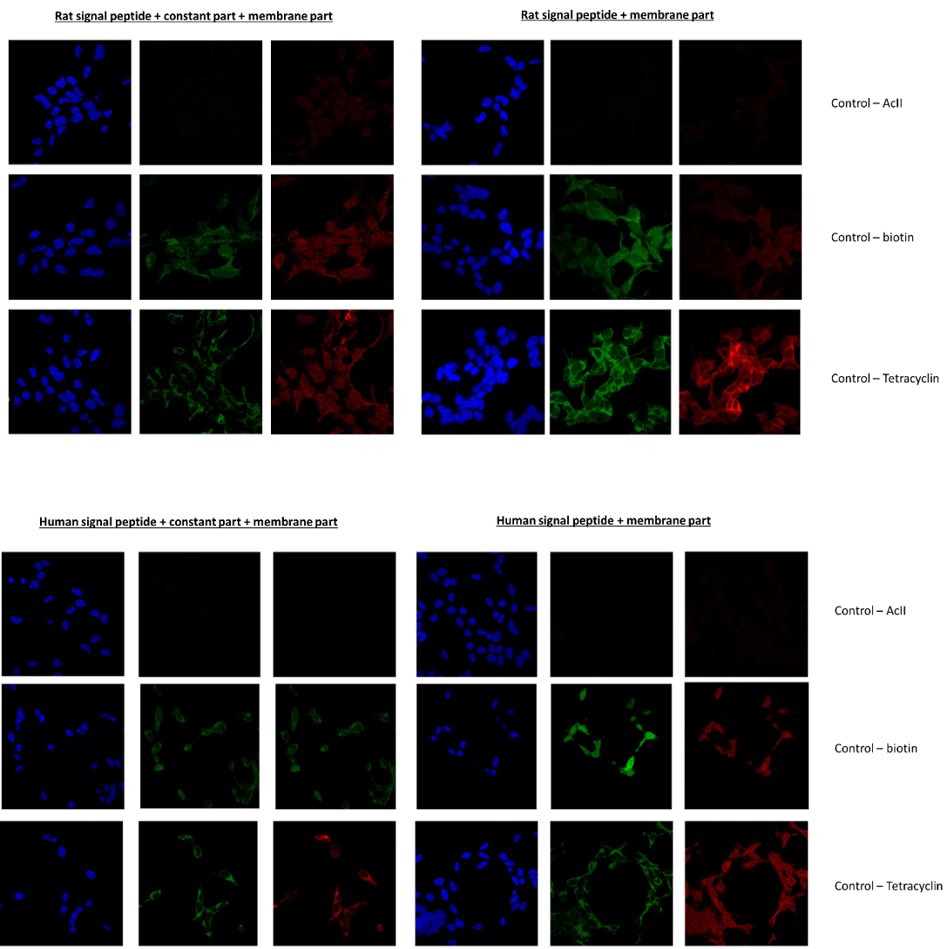

Supplement: Supplementary file 12 — Supp Figure 7 [file 41419_2023_5737_MOESM12_ESM.jpg]
