# Supplementary material for: Astrocytes express aberrant immunoglobulins as putative gatekeeper of astrocytes to neuronal progenitor conversion
Source: Cell Death Dis. 2023 Apr 4;14(4):237. doi: 10.1038/s41419-023-05737-9 (PMC10073301; doi:10.1038/s41419-023-05737-9)
Supplement: Supplementary file 5 — Supp Figures Legends [file 41419_2023_5737_MOESM5_ESM.docx]

**Supplementary Figures**

**Supp. Figure 1** : **A)** RT-PCR amplification of *Gfap* in rat DI TNC1 astrocyte cell line and rat primary spinal astrocytes stimulated or not with 200 ng/mL of LPS during the time points indicated. H_2_O: negative control. **B)** Western blot analyses of GFAP in DI TNC1 cells and rat primary cortex and spinal astrocytes stimulated or not with 200 ng/mL of LPS during 24h or 48H as well as in spleen.

**Supp. Figure 2** : Comparison of rat IgG2B sequences obtained from transcriptomic and proteomic analyses performed on DI TINC1 cells and rat primary cortex and spinal astrocytes with data obtained from spinal cord injury ^8^.

**Supp. Figure 3** : RT-PCR amplification of IgHV and IgKV variable chains in rat primary spinal or cortex astrocytes. cDNA from spleen served as a positive control. H_2_O and negative Reverse Transcriptase (RT-) serve as negative controls

**Supp. Figure 4** : **A)** Sequences alignment of different IgVH retrieved from RNAseq analyses performed on DI TNC1 cells and rat primary cortex and spinal astrocytes. These sequences were confirmed by RT-PCR amplification. **B)** Rat IgM sequence characterized by RNAseq, RT-PCR and proteomic analyses carried out on rat primary spinal and cortex astrocytes **C)** RT-PCR amplification of IgG2B constant **a**) and variable heavy chains **b**) coding sequences from primary astrocytes from adult rats. cDNA from spleen served as a positive control. **c**) RT-PCR amplification of *kappa* light constant chains and *kappa* light variable chain mRNA from embryonic primary culture of human astrocytes. The experiment was also performed on a negative reverse transcriptase sample to rule out genomic DNA contamination. H_2_0: negative control.

**Supp. Figure 5** : String analyses of the specific proteins identified after *IgH6*-1 KO or *IgH6*-2 KO.

**Supp. Figure 6: A)** Anti-Flag Western blot analyses from Gel electrophoresis in non-reducing conditions of IgGH constructs with or without TM domain in DI TNC1, cells transduced with empty vector (EV) or LPS-stimulated for 24h, 48h or 72h. **B)** anti-IgKV Western blot analyses under reducing conditions of DI TNC1 cells as well as spinal and cortex primary astrocytes stimulated or not with LPS , **C)** Western blot analyses of secretome of LPS-stimulated CRIPSR-CAS 9 (IgH6-1, IgH6-2) DI TNC1 cells versus T2 (Trop2), control, polybrene and empty vector transfected cells with anti-Heimdall **D)** Venn diagram of the three conditions with controls and EV conditions.

**Supp. Figure 7** : Proximal labeling study in HEK 293 cells with rat IgG2B transmembrane form as a bait in control conditions. Detection of biotinylated proteins (green, Streptavidin Alexa Fluor 488 conjugate) and rat IgG2B (red, anti-Flag) in HEK293 cells after overexpression of IgG2B constant part in fusion with BirA*Flag. IgG2B was overexpressed with a rat or a human signal peptide. Nuclei were stained with DAPI (blue).

**Supp. Table 1 :** List of the primers used

**Supp. Table 2** : RT-PCR and RACE_PCR parameters
