# Supplementary material for: Astrocytes express aberrant immunoglobulins as putative gatekeeper of astrocytes to neuronal progenitor conversion
Source: Cell Death Dis. 2023 Apr 4;14(4):237. doi: 10.1038/s41419-023-05737-9 (PMC10073301; doi:10.1038/s41419-023-05737-9)
Supplement: Supplementary file 13 — Suppemental Informations data [file 41419_2023_5737_MOESM13_ESM.docx]

**SUPPLEMENTAL INFORMATION**

**Data S1**: Complete sequences coding rat IgG2B and IgM heavy constant chains and the variable heavy and light chains

**Data 1 bis** : Expression of IgL gene in Human astrocytes and Ighm, Igg1, Igg2, IgA and IgK genes in rodents' astrocytes. The thick lines indicate the sequences covered by the reads retrieved from Geo DataSets repository. The accession numbers corresponding to the different RNA seq experiments analyzed as well as the tissue origin of the astrocytes are indicated.

**Data S2:** Sequence and construct for overexpression of IgG2B with Kozak sequence, with or without the transmembrane domain

**Data S3:** Nucleic sequences coding all enzymes of the recombination V(D)J complex, CD20 and CD19. Sequences were identified in the spleen and astrocytes.

**Data S4:** List of exclusive proteins characterized by shotgun proteomic performed on extracts from DI TNC1 cells after *IgH6*-1 KO, *Igh6*-2 KO, *Heimdall* KO (KO2), control cells treated or not with polybrene, infection with an empty vector (EV) and *Trop2* KO as a non-target control, (n=3). Statistical significance was evaluated with the ANOVA test (p<0.01).

**Data S5:** List of proteins characterized in the clusters of the heatmap obtained after shotgun proteomic performed on extracts of DI TNC1 cells after *IgH6*-1 KO, *Igh6*-2 KO, *Heimdall* KO (KO2), control cells treated or not with polybrene, infection with an empty vector (EV) and *Trop2* KO as a non-target control, (n=3). Statistical significance was evaluated with the ANOVA test (p<0.01).

**Data 5 Bis** : Detection of Notch 2 transmembrane/intracellular region (NTM) and intracellular region (NCID) forms as well as TGF-beta III by western blot. The experiments were performed on protein extracts from DI TNC1 cells after IgH6 KO, control cells, infection with empty vector (EV) and Trop2 KO as a non-target control.

**Data S6:** List of proteins identified by shotgun proteomic after overexpression of the Kozak, IgG2B with or without the transmembrane domain or with only the Kozak sequencer and the transmembrane domain compared to empty vector and control.

**Data S7:** List of proteins identified by shotgun proteomic after proximal labeling carried out in HEK293 cells using rat IgG2B transmembrane form fused to BirA*Flag as a bait.

**Data S8**: List of proteins identified by shotgun proteomic performed on spots highlighted by western blot carried out on 2D gel. This western blot was conducted on protein extracts of DI TNC1 astrocytes using secretome of DI TNC1 astrocytes stimulated for 24h with 200 ng/mL of LPS.
