# Supplementary material for: Astrocytes express aberrant immunoglobulins as putative gatekeeper of astrocytes to neuronal progenitor conversion
Source: Cell Death Dis. 2023 Apr 4;14(4):237. doi: 10.1038/s41419-023-05737-9 (PMC10073301; doi:10.1038/s41419-023-05737-9)
Supplement: Supplementary file 15 — Supplementary Dataset 1bis [file 41419_2023_5737_MOESM15_ESM.pdf]

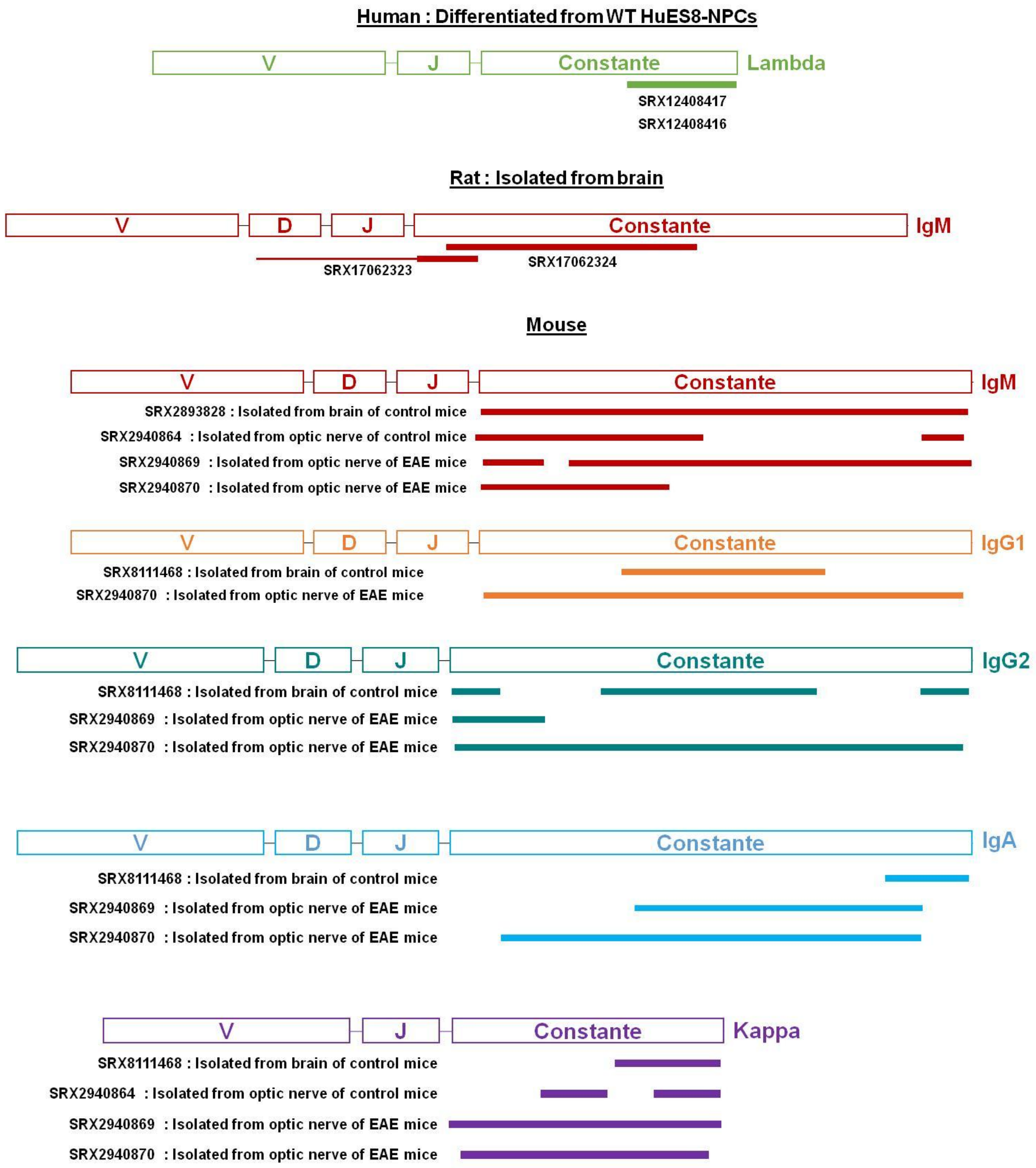

Supp. Data 1 bis : Expression of *IgL* gene in Human astrocytes and *Ighm*, *Igg1*, *Igg2*, *IgA* and *IgK* genes in rodents' astrocytes. The thick lines indicate the sequences covered by the reads retrieved from Geo DataSets repository. The accession numbers corresponding to the different RNA seq experiments analyzed as well as the tissue origin of the astrocytes are indicated.
