# Supplementary material for: Astrocytes express aberrant immunoglobulins as putative gatekeeper of astrocytes to neuronal progenitor conversion
Source: Cell Death Dis. 2023 Apr 4;14(4):237. doi: 10.1038/s41419-023-05737-9 (PMC10073301; doi:10.1038/s41419-023-05737-9)
Supplement: Supplementary file 16 — Supplementary Dataset 2 [file 41419_2023_5737_MOESM16_ESM.docx]

**I Design : primers**

- Human Signal Peptide + IgG2b constante + membrane part

1266NT=421AA+stop

ATGGAGTTTGGGCTGAGCTGGGTTTTCCTTGTTGTTATTTTACAAGGTGTCCAGTGT

**G** CC CAAACAAC AGCCCCATCT GTCTATCCAC TGGCTCCTGG ATGTGGTGAT ACAACCAGCT CCACGGTGAC CCTGGGATGC CTGGTCAAGG GCTATTTCCC TGAGCCAGTC ACCGTGACCT GGAACTCTGGAGCCCTGTCC AGCGATGTGC ACACCTTCCC AGCTGTCCTG CAGTCTGGGC TCTACACTCT CACCAGCTCA GTGACCTCCA GCACCTGGCC CAGCCAGACC GTCACCTGCA ACGTAGCCCA CCCGGCCAGCAGCACCAAGG TGGACAAGAA AATTGAGCGC AGAAATGACA ACATTGGACA CAAAAGC CCTACATGCC CTACATGTCA CAAATGCCCA GCTCCTGAAC TCTTGGGTGG ACCATCCGTC TTCATCTTCCCCCCAAAGCC CAAGGACATC CTCTTGATCT CCCAGAACGC CAAGGTCACG TGTGTGGTGG TGGATGTGAG CGAGGAGGAG CCGGACGTCC AGTTCAGCTG GTTTGTGAAC AACGTAGAAG TACACACAGCTCAGACACAA CCCCGTGAGG AGCAGTACAA CAGCACCTTC AGAGTGGTCA GTGCCCTCCC CATCCAGCAC CAGGACTGGA TGAGCGGCAA GGAGTTCAAA TGCAAGGTCA ACAACAAAG CCCTCCCAAGCCCCATCGAG AAAACCATCT CAAAACCCAA AGGGCTAGTC AGAAAACCAC AGGTATACGT CATGGGTCCA CCGACAGAGC AGTTGACTGA GCAAACGGTC AGTTTGACCT GCTTGACCTC AGGCTTCCTCCCTAACGACA TCGGTGTGGA GTGGACCAGC AACGGGCATA TAGAAAAGAA CTACAAGAAC ACCGAGCCAG TGATGGACTC TGACGGTTCT TTCTTCATGT ACAGCAAGCT CAATGTGGAA AGGAGCAGGTGGGATAGCAG AGCGCCCTTC GTCTGCTCCG TGGTCCACGA GGGTCTGCAC AATCACCACG TGGAGAAGAG CATCTCCCGG CCTCCG

GGG CTAGAAGTGGA TGATGATTGT GCTGAGGCTC AGGACGGGGAGCTGGACGGG CTCTGGACGA CCATCACCAT CTTCATCAGC CTCTTCCTGC TCAGTGTGTG CTACAGTGCC TCCATCACAC TCTTCAAGGT AAAGTGGATC TTCTCCTCAG TGGTGGAGCT GAAGCAGACAATC TCCC CTGACTACAG AAACATGATT GGTCAAGGAG CC TAG

AscI_HumPS+Cte+Membr

tataGGCGCGCCa ATGGAGTTTGGGCTGAGCTGGGTTTTCCTTGTTGTTATTTTACAAGGTGTCCAGTGT G CC CAAACAAC AGCCCCATC

NotI_BF_Ig2bMembr

ttaaGCGGCCGC GGCTCCTTGACCAATCATGTTTCTG

MEFGLSWVFLVVILQGVQCAQTTAPSVYPLAPGCGDTTSSTVTLGCLVKGYFPEPVTVTWNSGALSSDVHTFPAVLQSGLYTLTSSVTSSTWPSQTVTCNVAHPASSTKVDKKIERRNDNIGHKSPTCPTCHKCPAPELLGGPSVFIFPPKPKDILLISQNAKVTCVVVDVSEEEPDVQFSWFVNNVEVHTAQTQPREEQYNSTFRVVSALPIQHQDWMSGKEFKCKVNNKALPSPIEKTISKPKGLVRKPQVYVMGPPTEQLTEQTVSLTCLTSGFLPNDIGVEWTSNGHIEKNYKNTEPVMDSDGSFFMYSKLNVERSRWDSRAPFVCSVVHEGLHNHHVEKSISRPPGLEVDDDCAEAQDGELDGLWTTITIFISLFLLSVCYSASITLFKVKWIFSSVVELKQTISPDYRNMIGQGA-

- Human Signal Peptide + membrane part

273NT=90AA+stop

ATGGAGTTTGGGCTGAGCTGGGTTTTCCTTGTTGTTATTTTACAAGGTGTCCAGTGT

GG G CTAGAAGTGGA TGATGATTGT GCTGAGGCTC AGGACGGGGAGCTGGACGGG CTCTGGACGA CCATCACCAT CTTCATCAGC CTCTTCCTGC TCAGTGTGTG CTACAGTGCC TCCATCACAC TCTTCAAGGT AAAGTGGATC TTCTCCTCAG TGGTGGAGCT GAAGCAGACAATC TCCC CTGACTACAG AAACATGATT GGTCAAGGAG CC TAG

AscI_HumPS+MembrSeule

tataGGCGCGCCa ATGGAGTTTGGGCTGAGCTGGGTTTTCCTTGTTGTTATTTTACAAGGTGTCCAGTGT GG G CTAGAAGTGGA TGATG

MEFGLSWVFLVVILQGVQCGLEVDDDCAEAQDGELDGLWTTITIFISLFLLSVCYSASITLFKVKWIFSSVVELKQTISPDYRNMIGQGA-

- Rat Signal Peptide + IgG2b constante + membrane part

1266NT=41AA+stop

ATGAAGTTGTGGCTGAACTGGATTTCCCTTTTAACACTTTTAAAAGGTATCCAGTGT

**G** CC CAAACAAC AGCCCCATCT GTCTATCCAC TGGCTCCTGG ATGTGGTGAT ACAACCAGCT CCACGGTGAC CCTGGGATGC CTGGTCAAGG GCTATTTCCC TGAGCCAGTC ACCGTGACCT GGAACTCTGGAGCCCTGTCC AGCGATGTGC ACACCTTCCC AGCTGTCCTG CAGTCTGGGC TCTACACTCT CACCAGCTCA GTGACCTCCA GCACCTGGCC CAGCCAGACC GTCACCTGCA ACGTAGCCCA CCCGGCCAGCAGCACCAAGG TGGACAAGAA AATTGAGCGC AGAAATGACA ACATTGGACA CAAAAGC CCTACATGCC CTACATGTCA CAAATGCCCA GCTCCTGAAC TCTTGGGTGG ACCATCCGTC TTCATCTTCCCCCCAAAGCC CAAGGACATC CTCTTGATCT CCCAGAACGC CAAGGTCACG TGTGTGGTGG TGGATGTGAG CGAGGAGGAG CCGGACGTCC AGTTCAGCTG GTTTGTGAAC AACGTAGAAG TACACACAGCTCAGACACAA CCCCGTGAGG AGCAGTACAA CAGCACCTTC AGAGTGGTCA GTGCCCTCCC CATCCAGCAC CAGGACTGGA TGAGCGGCAA GGAGTTCAAA TGCAAGGTCA ACAACAAAG CCCTCCCAAGCCCCATCGAG AAAACCATCT CAAAACCCAA AGGGCTAGTC AGAAAACCAC AGGTATACGT CATGGGTCCA CCGACAGAGC AGTTGACTGA GCAAACGGTC AGTTTGACCT GCTTGACCTC AGGCTTCCTCCCTAACGACA TCGGTGTGGA GTGGACCAGC AACGGGCATA TAGAAAAGAA CTACAAGAAC ACCGAGCCAG TGATGGACTC TGACGGTTCT TTCTTCATGT ACAGCAAGCT CAATGTGGAA AGGAGCAGGTGGGATAGCAG AGCGCCCTTC GTCTGCTCCG TGGTCCACGA GGGTCTGCAC AATCACCACG TGGAGAAGAG CATCTCCCGG CCTCCG

GGG CTAGAAGTGGA TGATGATTGT GCTGAGGCTC AGGACGGGGAGCTGGACGGG CTCTGGACGA CCATCACCAT CTTCATCAGC CTCTTCCTGC TCAGTGTGTG CTACAGTGCC TCCATCACAC TCTTCAAGGT AAAGTGGATC TTCTCCTCAG TGGTGGAGCT GAAGCAGACAATC TCCC CTGACTACAG AAACATGATT GGTCAAGGAG CC TAG

AscI_RatPS+Cte+Membr

tataGGCGCGCCa ATGAAGTTGTGGCTGAACTGGATTTCCCTTTTAACACTTTTAAAAGGTATCCAGTGT **G** CC CAAACAAC AGCCCCATC

MKLWLNWISLLTLLKGIQCAQTTAPSVYPLAPGCGDTTSSTVTLGCLVKGYFPEPVTVTWNSGALSSDVHTFPAVLQSGLYTLTSSVTSSTWPSQTVTCNVAHPASSTKVDKKIERRNDNIGHKSPTCPTCHKCPAPELLGGPSVFIFPPKPKDILLISQNAKVTCVVVDVSEEEPDVQFSWFVNNVEVHTAQTQPREEQYNSTFRVVSALPIQHQDWMSGKEFKCKVNNKALPSPIEKTISKPKGLVRKPQVYVMGPPTEQLTEQTVSLTCLTSGFLPNDIGVEWTSNGHIEKNYKNTEPVMDSDGSFFMYSKLNVERSRWDSRAPFVCSVVHEGLHNHHVEKSISRPPGLEVDDDCAEAQDGELDGLWTTITIFISLFLLSVCYSASITLFKVKWIFSSVVELKQTISPDYRNMIGQGA-

- Rat Signal Peptide + membrane part

273NT=90AA+stop

ATGAAGTTGTGGCTGAACTGGATTTCCCTTTTAACACTTTTAAAAGGTATCCAGTGTGG G CTAGAAGTGGA TGATGATTGT GCTGAGGCTC AGGACGGGGA

GCTGGACGGG CTCTGGACGA CCATCACCAT CTTCATCAGC CTCTTCCTGC TCAGTGTGTG CTACAGTGCC TCCATCACAC TCTTCAAGGT AAAGTGGATC TTCTCCTCAG TGGTGGAGCT GAAGCAGACAATC TCCC CTGACTACAG AAACATGATT GGTCAAGGAG CC TAG

AscI_RatPS+MembrSeule

tataGGCGCGCCa ATGAAGTTGTGGCTGAACTGGATTTCCCTTTTAACACTTTTAAAAGGTATCCAGTGT GG G CTAGAAGTGGA TGATG

MKLWLNWISLLTLLKGIQCGLEVDDDCAEAQDGELDGLWTTITIFISLFLLSVCYSASITLFKVKWIFSSVVELKQTISPDYRNMIGQGA-

- Kozac + IgG2b constante + membrane part

GCAGCT ATG
**G** CC CAAACAAC AGCCCCATCT GTCTATCCAC TGGCTCCTGG ATGTGGTGAT ACAACCAGCT CCACGGTGAC CCTGGGATGC CTGGTCAAGG GCTATTTCCC TGAGCCAGTC ACCGTGACCT GGAACTCTGGAGCCCTGTCC AGCGATGTGC ACACCTTCCC AGCTGTCCTG CAGTCTGGGC TCTACACTCT CACCAGCTCA GTGACCTCCA GCACCTGGCC CAGCCAGACC GTCACCTGCA ACGTAGCCCA CCCGGCCAGCAGCACCAAGG TGGACAAGAA AATTGAGCGC AGAAATGACA ACATTGGACA CAAAAGC CCTACATGCC CTACATGTCA CAAATGCCCA GCTCCTGAAC TCTTGGGTGG ACCATCCGTC TTCATCTTCCCCCCAAAGCC CAAGGACATC CTCTTGATCT CCCAGAACGC CAAGGTCACG TGTGTGGTGG TGGATGTGAG CGAGGAGGAG CCGGACGTCC AGTTCAGCTG GTTTGTGAAC AACGTAGAAG TACACACAGCTCAGACACAA CCCCGTGAGG AGCAGTACAA CAGCACCTTC AGAGTGGTCA GTGCCCTCCC CATCCAGCAC CAGGACTGGA TGAGCGGCAA GGAGTTCAAA TGCAAGGTCA ACAACAAAG CCCTCCCAAGCCCCATCGAG AAAACCATCT CAAAACCCAA AGGGCTAGTC AGAAAACCAC AGGTATACGT CATGGGTCCA CCGACAGAGC AGTTGACTGA GCAAACGGTC AGTTTGACCT GCTTGACCTC AGGCTTCCTCCCTAACGACA TCGGTGTGGA GTGGACCAGC AACGGGCATA TAGAAAAGAA CTACAAGAAC ACCGAGCCAG TGATGGACTC TGACGGTTCT TTCTTCATGT ACAGCAAGCT CAATGTGGAA AGGAGCAGGTGGGATAGCAG AGCGCCCTTC GTCTGCTCCG TGGTCCACGA GGGTCTGCAC AATCACCACG TGGAGAAGAG CATCTCCCGG CCTCCG

AA

GGG CTAGAAGTGGA TGATGATTGT GCTGAGGCTC AGGACGGGGAGCTGGACGGG CTCTGGACGA CCATCACCAT CTTCATCAGC CTCTTCCTGC TCAGTGTGTG CTACAGTGCC TCCATCACAC TCTTCAAGGT AAAGTGGATC TTCTCCTCAG TGGTGGAGCT GAAGCAGACAATC TCCC CTGACTACAG AAACATGATT GGTCAAGGAG CC TAG

Cte Ig2b avec Kozak et ATG + Partie membranaire + FLAG

Kozac_IgG2b_Fwd

AGTCCTAGCAGCTATGGCCCAAACAACAGCCCCATCTGTCTATCCACTGG

ATG_IgG2b_AscI_Fwd

tataGGCGCGCCa GCAGCT ATG G CC CAAACAAC AGC

NotI_BF_Ig2bMemb

ttaaGCGGCCGC GGCTCCTTGACCAATCATGTTTCTG

Cte_IgG2b_NotI_Rev

ttaaGCGGCCGC CGGAGGCCGGGAGATGCTCTTCTCC

MembrSeule_Kozak_AscI_Fwd

tataGGCGCGCCa GCAGCTATGGCCC aa GGG CTAGAAGTGGA TGATGATTG
