# Supplementary material for: Astrocytes express aberrant immunoglobulins as putative gatekeeper of astrocytes to neuronal progenitor conversion
Source: Cell Death Dis. 2023 Apr 4;14(4):237. doi: 10.1038/s41419-023-05737-9 (PMC10073301; doi:10.1038/s41419-023-05737-9)

# Sequencing

## 1. Identification of *gfap* mRNA from rat astrocytes (DI TNC1, spinal cord and cortex)

```
ATGGAGCGGAGACGTATCACCTCTGCACGCCGCTCCTATGCCTCCTCCGAGACGATGGTC
AGGGGCCATGGTCCTACCAGACACCTGGGTACCATTCCGCGCCTCTCCCTGTCTCGAATG
ACGCCTCCACTCCCTGCCAGGGTAGACTTCTCCCTGGCCGGGGCGCTCAATGCCGGCTTC
AAAGAGACTCGGGCCAGCGAGCGCGCGGAGATGATGGAGCTCAATGACCGCTTTGCTAGC
TACATCGAGAAGGTCCGCTTCCTGGAACAGCAAAACAAGGCGCTGGCAGCTGAGCTGAAC
CAGCTTCGAGCCAAGGAGCCCACCAAACTGGCTGACGTTTACCAGGCAGAACTTCGGGAG
CTGCGGCTGCGTCTGGACCAGCTTACTACCAACAGTGCCCGGCTGGAGGTGGAGAGGGAC
AATCTCACACAGGACCTCGGCACCCTGAGGCAGAAGCTCCAAGATGAAACCAACCTGAGG
CTGGAGGCGGAGAACAACCTGGCTGTGTACAGACAGGAGGCGGATGAAGCCACCTTGGCT
CGTGTGGATCTGGAGAGGAAGGTTGAGTCGCTGGAGGAGGAGATCCAGTTCTTGAGGAAG
ATCCATGAGGAGGAAGTTCGAGAACTCCAGGAGCAGCTGGCCCAGCAGCAgGTCCACGTG
GAGATGGATGTGGCCAAGCCAGACCTCACAGCGGCTCTGAGAGAGATTCGCACTCAGTAC
GAGGCAGTGGCCACCAGTAACATGCAAGAAACAGAAGAGTGGTATCGGTCCAAGTTTGCA
GACCTCACAGACGTTGCTTCCCGCAACGCAGAGCTGCTCCGCCAGGCCAAGCACGAGGCT
AATGACTATCGCCGCCAACTGCAGGCCTTGACCTGCGACCTTGAGTCCTTGCGCGGCACG
AACGAGTCCTTGGAGAGGCAAATGCGCGAACAGGAGGAGCGCCACGCGCGGGAGTCGGCG
AGTTACCAGGAGGCACTCGCTCGGCTGGAGGAGGAGGGCCAAAGCCTCAAGGAGGAGATG
GCCCGCCACCTGCAGGAGTACCAGGATCTACTCAACGTTAAGCTAGCCCTGGACATCGAG
ATCGCCACCTACAGGAAATTGCTGGAGGGCGAAGAAAACCGCATCACCATTCCTGTACAG
ACTTTCTCCAACCTCCAGATCCGAGAAACCAGCCTGGACACCAAATCTGTGTCAGAAGGC
CACCTCAAGAGGAACATCGTGGTAAAGACGGTGGAGATGCGGGATGGCGAGGTCATTAAG
GAGTCGAAGCAGGAGCACAAGGATGTGATGTGA
```

Alignment with the reference sequence

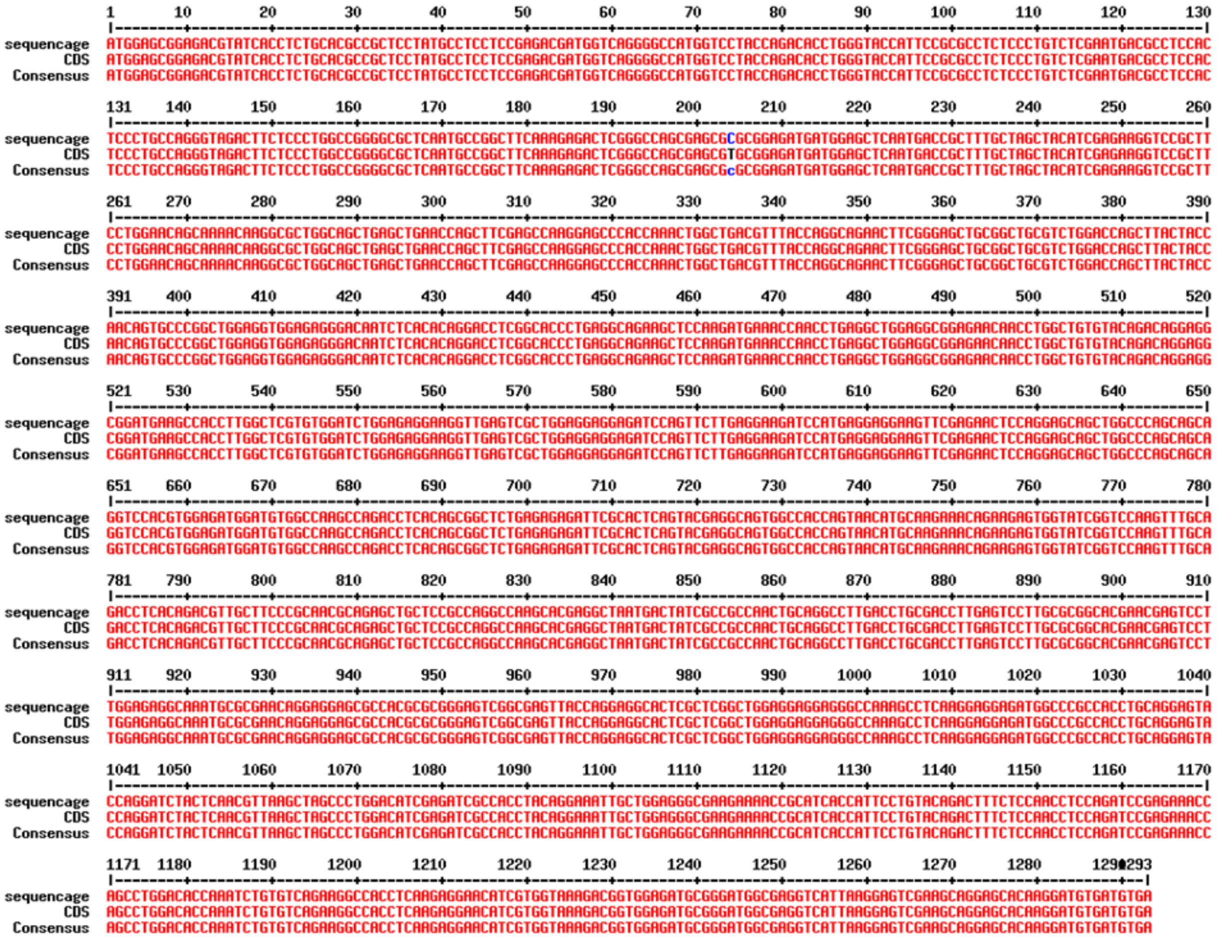

## 2. Identification of *CD19* mRNA from rat spleen

```
ATGCCATCTCCTCTCCCTGTCTCCCTCCTCCTCTTCCTTACCTTAGTAGGAGGCAGGCCC
CAGAATTCCTTACTGGTGGAGGTAGAAGAGGGAGACAATGTTGTGCTGTCATGCCTCCGG
GACTCCTCACCTGTCTCTTCTGAGAAGCTGGCTTGGTATCGAGGTAACCAGTCAACACCC
TTCCTGGAGCTGAGCCTCAGGTCCCCGGACCTGGGTCTGCACATCGGGCCCCTGGGCATC
TTGCTAGTGATTGTCAATGTCTCGGACCATAGGGGGGGCTTCTATCTGTGCCAAAAGAGG
CCCTCTTTCAAGGACACCTGGCAGCCTGCCTGGACCGTAAACGTGGAGGATAGTGGGGAG
CTGTTCCGGTGGAATGCTTCAGACTTGGGGGACCTGGATTGTGACCTAGGAAACAGGTCC
TCAGGGAGCCACAGGTCCACTTCTGGTTCCCAGCTGTATGTGTGGGCTACAGATCATCCT
GAGGTCTGGAAAACAAAGCCTGTATGTGCCCCACGGGAGATCAGTCTGAATCAGAGTCTA
ATCAACCAAGATCTCACTGTGGCCCCTGGCTCCACACTTTGGCTGTCCTGTGGCGTACCC
CCTGTCCCAGTGACCAAAGGCTCCATCTCCTGGACCCATGTGCATCCTAAGACGCTTAAC
GTTTCATTACTGAGCCTAAGCCTTGGGGGAGAGCACCCAGTGAGAGAGATGTGGGTTTGG
GGGTCTCTTTTGCTACTGCCCCAAGCCAAAGCTTCAGATGAAGGTACCTATTATTGTC
TCCAAGGAGGCCTGACCATCAAGATGCACGTGAAGGTCATTGCAAGGTCAGCAGTGTGGC
TCTGGCTGTTGAGGACTGGTGGATGGATAGTCCCAGTTGTGACTTTAGTATATGTCATCT
TCTGTATGGTTTCTATGGCGGCTTTTCTCTATTTTCGAAGAGCCTTCATCCTGAGGAGGA
AGAGGAAGCGAATGACCGACCCTGCTAGGAGATTCTTCAAAGTGACGCCTCCCTCAGGAA
ACGGGACCCAGAACCAGTATGGGAATGTGCTCTCCCTTCCTACATCTACCTCTGGCCAGG
CCCATGCTCAGCGTTGGGCTGCTAGCCTAGGGAGTGGTCCTGTGTCCTATGGAAATCCAC
GCATTGAAGTCCAGGATGCTGGAGCTCAGAGACATGAAACAGGACTGGAAGAAGAAGGGG
AGGCCTATGAAGAGCCAGACAGCGAGGAGGGCTCTGAATTCTATGAGAATGACTCCAACC
TTGAGCAGGATCAACTTTCCCAGGATGGCAGTGGCTATGAGAACCCTGAGGATGACCCCG
TAGGTCCAGAGGAGGAAGACTCCTTCTCCAATGCCGAGTCTTATGAAAATGCAGATGAGG
AGCTGGCCCAACCAGTTGGCAGGACGATGGACTTCCTGAGCCCCCACGGGTCTGCATGGG
ACCCCAGCCGGGAAGCATCCTCCCTTGGGTCCCAGTCCTACGAAGATATGAGAGGGATCC
TGTATGCAGCTCCTCAGCTCCAATCAATTCGGTCTGGTCCCAGTCATGAGGAAGATGCAG
ACTCTTATGAAAACATGGATAAATCTGACGACCCAGAGCCAGCATGGGCAGGAGAGGGCC
ACATGGGGACTTGGGGTGCCACGTGA
```

Alignment with the reference sequence

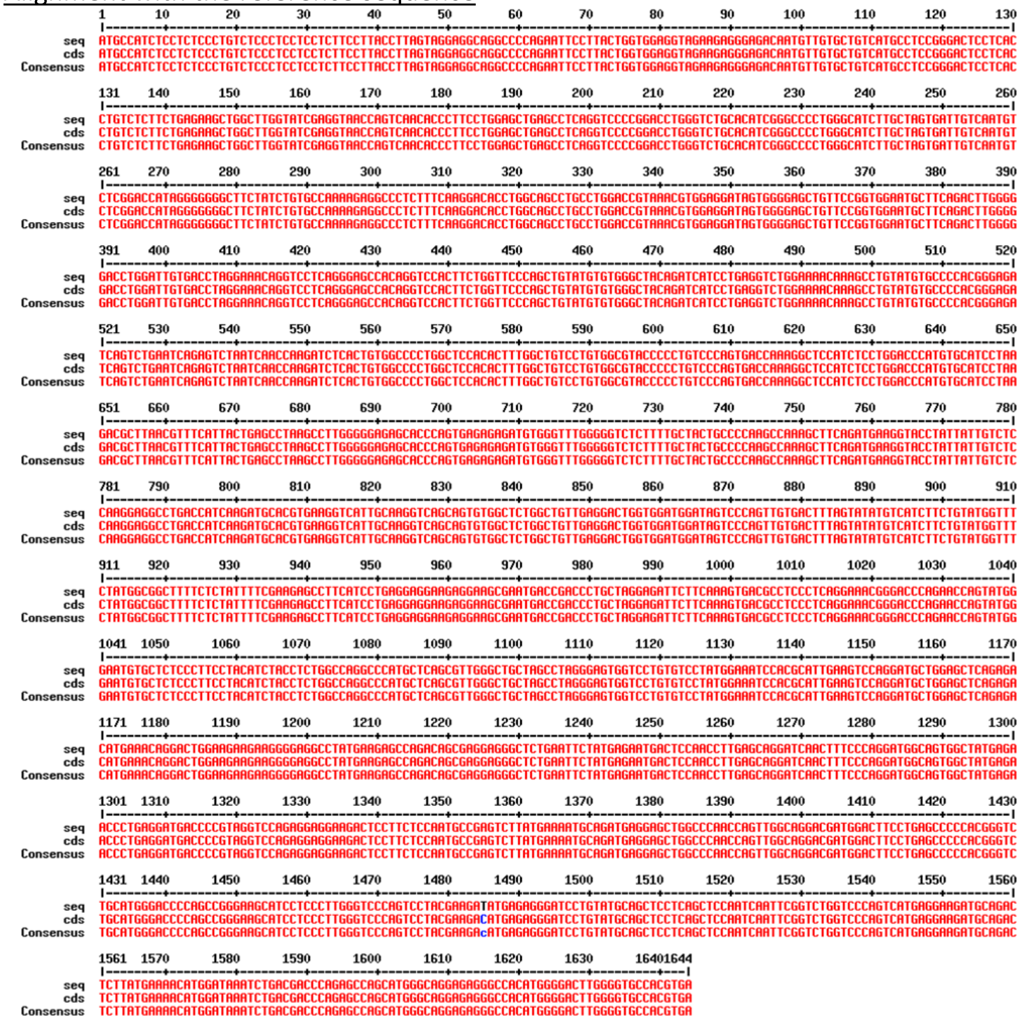

### 3. Identification of *Ms4a1* (CD20*)* mRNA from rat spleen

```
ATGACAACACCCAGAAATTCAGTGAGTGGACCTTTCCCAACAGAGCCTACAAAAGGACCC
CTCGCCATGCAACCTGCTCAAAAAGTAATCCCCAGAAGACCATCTTCACTGGTGGGCCCC
ACACAAAGCTTCTTCATGAGGGAATCAAAGGCTTTGGGGGCTGTCCAGATCATGAATGGC
CTCTTCCACATTTCCCTGGGGGGACTGCTGATGATCCCCACAGGAGTTTTTGCACCCATC
TGTTTGAGTGTATGGTACCCTCTGTGGGGAGGCATTATGTACATCATTTCAGGATCACTC
CTGGCAGCTGCAGCAGAGAAAACCTCCAGGAAGAGTTTGGTCAAAGCGAAAGTGATAATG
AGCTCTCTAAGCCTCTTTGCTGCCATTTCTGGAATAATTCTTTCAATCATGGACATACTT
AACATTACAGTTTCTCATTTTTTAAAAATGGAGAGACTGGAACTGATTAAAACTCCCAAG
CTATATGTTGATATCTACAACTGTGAGCCATCCAATTCCTCAGAGAAAAACTCCCCATCT
ACACAGTACTGTAACAGCGTGCAGTCTGTGTTCTTGGGCATTCTGTCGGTGATGCTGATC
TCTGCCTTCTTCCAgAAGCTCGTGACAGCCGGTGTTGTAGAGAATGAGTGGAAAAGAGTG
TGCTCCAGGCCCAAATCTAATGTGGTTCTGCTGTCAGCTGGAGAAAAAAAAGAGCAGACA
ATTAAAATGAAAGAAGAAATCATTGAGCTAAGTGGAGTATCTTCCCAACCAAAGAATGAA
GAGGAAATTGAAATTATTCCAGTGCAGGAGGAAGAAGAAGAAGAAGCAGAAATAAACTTC
CCAGCACCTCCCCAAGAGCAGGAATCCTTGCCAGTGGAAAATGAGATCTCTCCTTAA
```

Alignment with the reference sequence

```
           1       10        20        30        40        50        60        70        80        90        100       110       120       130
sequenc    ATGACAACACCCAGAAATTCAGTGAGTGGACCTTTCCCAACAGAGCCTACAAAAGGACCCCTCGCCATGCAACCTGCTCAAAAAGTAATCCCCAGAAGACCATCTTCACTGGTGGGCCCCACACAAAGCT
CDS        ATGACAACACCCAGAAATTCAGTGAGTGGACCTTTCCCAACAGAGCCTACAAAAGGACCCCTCGCCATGCAACCTGCTCAAAAAGTAATCCCCAGAAGACCATCTTCACTGGTGGGCCCCACACAAAGCT
Consensus  ATGACAACACCCAGAAATTCAGTGAGTGGACCTTTCCCAACAGAGCCTACAAAAGGACCCCTCGCCATGCAACCTGCTCAAAAAGTAATCCCCAGAAGACCATCTTCACTGGTGGGCCCCACACAAAGCT

           131     140       150       160       170       180       190       200       210       220       230       240       250       260
sequenc    TCTTCATGAGGGAATCAAAGGCTTTGGGGGCTGTCCAGATCATGAATGGCCTCTTCCACATTTCCCTGGGGGGACTGCTGATGATCCCCACAGGAGTTTTTGCACCCATCTGTTTGAGTGTATGGTACCC
CDS        TCTTCATGAGGGAATCAAAGGCTTTGGGGGCTGTCCAGATCATGAATGGCCTCTTCCACATTTCCCTGGGGGGACTGCTGATGATCCCCACAGGAGTTTTTGCACCCATCTGTTTGAGTGTATGGTACCC
Consensus  TCTTCATGAGGGAATCAAAGGCTTTGGGGGCTGTCCAGATCATGAATGGCCTCTTCCACATTTCCCTGGGGGGACTGCTGATGATCCCCACAGGAGTTTTTGCACCCATCTGTTTGAGTGTATGGTACCC

           261     270       280       290       300       310       320       330       340       350       360       370       380       390
sequenc    TCTGTGGGGAGGCATTATGTACATCATTTCAGGATCACTCCTGGCAGCTGCAGCAGAGAAAACCTCCAGGAAGAGTTTGGTCAAAGCGAAAGTGATAATGAGCTCTCTAAGCCTCTTTGCTGCCATTTCT
CDS        TCTGTGGGGAGGCATTATGTACATCATTTCAGGATCACTCCTGGCAGCTGCAGCAGAGAAAACCTCCAGGAAGAGTTTGG-CAA--CGAAAGTGATAATGAGCTCTCTAAGCCTCTTTGCTGCCATTTCT
Consensus  TCTGTGGGGAGGCATTATGTACATCATTTCAGGATCACTCCTGGCAGCTGCAGCAGAGAAAACCTCCAGGAAGAGTTTGG.CAA..CGAAAGTGATAATGAGCTCTCTAAGCCTCTTTGCTGCCATTTCT

           391     400       410       420       430       440       450       460       470       480       490       500       510       520
sequenc    GGAATAATTCTTTCAATCATGGACATACTTAACATTACAGTTTCTCATTTTTTAAAAATGGAGAGACTGGAACTGATTAAAACTCCCAAGCTATATGTTGATATCTACAACTGTGAGCCATCCAATTCCT
CDS        GGAATAATTCTTTCAATCATGGACATACTTAACATTACAGTTTCTCATTTTTTAAAAATGGAGAGACTGGAACTGATTAAAACTCCCAAGCTATATGTTGATATCTACAACTGTGAGCCATCCAATTCCT
Consensus  GGAATAATTCTTTCAATCATGGACATACTTAACATTACAGTTTCTCATTTTTTAAAAATGGAGAGACTGGAACTGATTAAAACTCCCAAGCTATATGTTGATATCTACAACTGTGAGCCATCCAATTCCT

           521     530       540       550       560       570       580       590       600       610       620       630       640       650
sequenc    CAGAGAAAAACTCCCCATCTACACAGTACTGTAACAGCGTGCAGTCTGTGTTCTTGGGCATTCTGTCGGTGATGCTGATCTCTGCCTTCTTCCAGAAGCTCGTGACAGCCGGTGTTGTAGAGAATGAGTG
CDS        CAGAGAAAAACTCCCCATCTACACAGTACTGTAACAGCGTGCAGTCTGTGTTCTTGGGCATTCTGTCGGTGATGCTGATCTCTGCCTTCTTCCAGAAGCTCGTGACAGCCGGTGTTGTAGAGAATGAGTG
Consensus  CAGAGAAAAACTCCCCATCTACACAGTACTGTAACAGCGTGCAGTCTGTGTTCTTGGGCATTCTGTCGGTGATGCTGATCTCTGCCTTCTTCCAGAAGCTCGTGACAGCCGGTGTTGTAGAGAATGAGTG

           651     660       670       680       690       700       710       720       730       740       750       760       770       780
sequenc    GAAAAGAGTGTGCTCCAGGCCCAAATCTAATGTGGTTCTGCTGTCAGCTGGAGAAAAAAAAGAGCAGACAATTAAAATGAAAGAAGAAATCATTGAGCTAAGTGGAGTATCTTCCCAACCAAAGAATGAA
CDS        GAAAAGAGTGTGCTCCAGGCCCAAATCTAATGTGGTTCTGCTGTCAGCTGGAGAAAAAAAAGAGCAGACAATTAAAATGAAAGAAGAAATCATTGAGCTAAGTGGAGTATCTTCCCAACCAAAGAATGAA
Consensus  GAAAAGAGTGTGCTCCAGGCCCAAATCTAATGTGGTTCTGCTGTCAGCTGGAGAAAAAAAAGAGCAGACAATTAAAATGAAAGAAGAAATCATTGAGCTAAGTGGAGTATCTTCCCAACCAAAGAATGAA

           781     790       800       810       820       830       840       850       860       870       880       890    897
sequenc    GAGGAAATTGAAATTATTCCAGTGCAGGAGGAAGAAGAAGAAGAAGCAGAAATAAACTTCCCAGCACCTCCCCAAGAGCAGGAATCCTTGCCAGTGGAAAATGAGATCTCTCCTTAA
CDS        GAGGAAATTGAAATTATTCCAGTGCAGGAGGAAGAAGAAGAAGAAGCAGAAATAAACTTCCCAGCACCTCCCCAAGAGCAGGAATCCTTGCCAGTGGAAAATGAGATCTCTCCTTAA
Consensus  GAGGAAATTGAAATTATTCCAGTGCAGGAGGAAGAAGAAGAAGAAGCAGAAATAAACTTCCCAGCACCTCCCCAAGAGCAGGAATCCTTGCCAGTGGAAAATGAGATCTCTCCTTAA
```

## 4. Identification of *Dclre1b* mRNA from rat astrocytes (DI TNC1, spinal cord and cortex)

```
ATGAACGGGGTCGTAATCCCCCAAACACCCATCGCTGTGGATTTTTGGAGCCTGCGCCGT
GCTGGTACCGCGCGGCTCTTCTTCTTATCCCACATGCACTGTGACCACACGGTGGGCCTG
TCTAGCACTTGGGCACGGCCCCTCTACTGCTCTCCCATCACTGCCCACCTCTTGCATCGT
CGCCTGCAGGTGTCTAAGCAGTGGATCCGAGCTCTGGAGATTGGTGAGAGCCATGTATTA
CCTCTAGATGAAATTGGACAAGAAACCATGACTGTAACCCTCATAGATGCCAATCACTGT
CCTGGTTCTGTCATGTTTCTCTTTGAAGGATACTTTGGAACAATTCTCTACACAGGTGAT
TTTCGATATACACCGTCCATGCTGAAGGAGCCTGCTCTGACACTAGGGAAACAGATTCAT
ACTTTATATCTAGACAACACCAATTGCAACCCAGCCCTTGTTCTTCCTTCCCGACAGGAA
GCTACTCAACAGATTATCCAGCTAATCCGACAGTTCCCCCAACACAACATAAAGATTGGC
CTCTATAGTCTAGGAAAAGAATCACTGCTGGAGCAGCTAGCCCTTGAGTTTCAGACCTGG
GTGGTATTGAGTCCTCAACGCCTGGAGTTGGTACAGCTGCTGGGCCTGGCAGACGTGTTC
ACAGTGGAGGAAGAAGCTGGGCGAATCCACGCTGTGGACCACATGGAAATCTGCCATTCG
GcCATGCTTCAgTGGAACCAGACCCACCCCACCATTGCTATTTTCCCCACAAGCCGGAAA
ATACGAAGCCCTCACCCCAGCATCTACAGCATCCCTTACTCTGACCATTCATCCTACTCT
GAGCTTCGAGCATTTGTTGCAGCTCTGAGGCCTTGCCAGGTGGTGCCCATAGTCCGTGAA
CAGCCTTGTGGAGAGTTTTTTTCAGGATAG
```

<u>Alignment with the reference sequence</u>

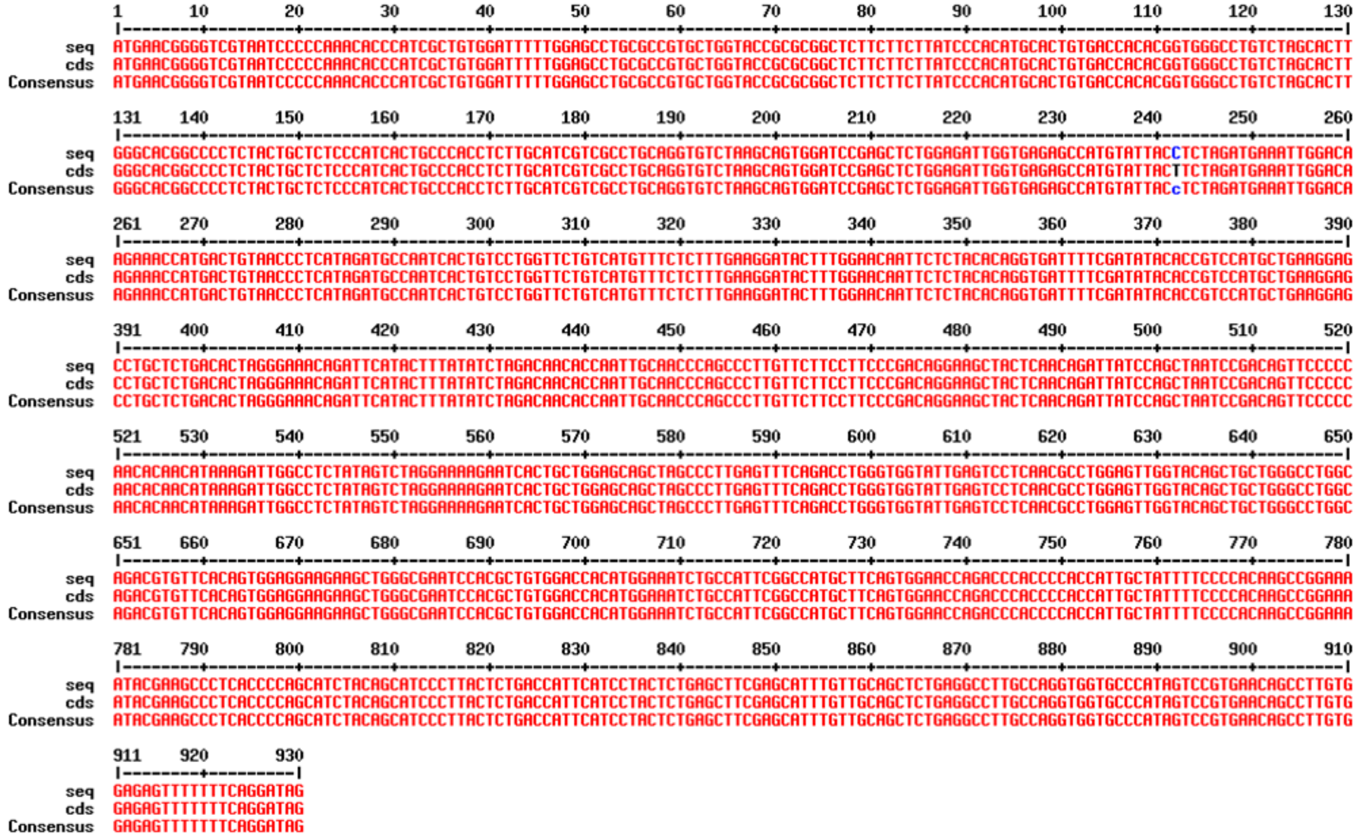

## 5. Identification of *Dclre1c* mRNA from rat astrocytes (DI TNC1, spinal cord)

```
ATGAGTTCTTTCCAGGGACAGATGGAGGAATATCCAACCATCTCCATTGACCGCTTCGAC
AGAGAGAACCTGAAAGCCCGTGCCTACTTCCTTTCGCACTGCCACAAGGATCACATGAAA
GGATTAAGGGCTCCTTCCATGAAAAGAAGGCTGGAATGCAGTTTGAAGGTCTTCCTGTAC
TGTTCTCCAGTCACTAAGGAGCTGCTCTTAACCAGCCCAAAGTACAAGTTCTGGGAAAAC
CGAATTATAGCAATTGAAATTGAAACTCCTACCCAGGTATCTTTAGTTGATGAGGCATCG
GGTGAGAAGGAAGAAGTTGTTGTGACTCTCTTACCAGCTGGTCACTGCCCAGGTTCAGTT
ATGTTTCTATTTCAGGGCAGTAATGGAACTGTCTTATACACAGGAGACTTCAGACTGGCG
AAAGGAGAAGTTTCCAGAATGGAGCTTCTGCACTCTGGGGGCAGAGTAAAAGACATCCAA
AGTGTATATTTAGACACCACTTTCTGTGACCCAAGGTTTTATCAAATTCCAAGTCGTGAG
GAGTGCTTGAGGGGCGTTCTGGAGCTGGTTCGGAGCTGGATCACGAGGAGTCCGAAGCAC
GTCGTGTGGCTGAACTGCAAGGCCGCCTACGGCTATGAGTACTTATTCACCAACCTGAGT
GAGGAGCTGGGAGTCCAGGTTCATGTGGACAAGCTGGACATGTTTAAAAACATGCCTGAT
ATCCTGCACCATCTCACAACGGATCGAAACACCCAGATCCACGCCTGCCGCCACCCGAAG
GCAGAAGAGTATTTTCAGTGGAATAAGTTACCCTGTGGCATGGCTTCCAAAACTAAAACT
GTACTCCACACAATCAGCATCAAGCCATCTACCATGTGGTTTGGAGAGAGGACCAGAAAA
ACAAACGTGATTGTGAGAACCGGAGAGAGCTCATACAGAGCTTGCTTCTCTTTCCACTCC
TCCTACAGTGAGATTAAAGATTTTTTGAGCTACATCTGCCCAGTGAATGCGTATCCAAAT
GTCATTCCGATCGGCCTCACTGTGGATAAGGTCATGGACTTTTTAAAGCCTCTGTGCCGC
TCTTCCCAATGTGCTGAACCAAAGTACAAGCCCCTCGGGAAACTGAAGAGAGCTAGAACA
GTTCACCTTGACTCAGAGGAAGATGATGATCTCTTTGATGACCCTCTACTAACACATTCA
AGGCGCAAAGTTCCGTACCAAGTAACTCTTCACCCTGAGGTATTTTCAATGAAGGCACTA
CCGCTAGATCAGCCTGAACTGGGACAAAGCCCCGGATGCTGTAAAGCAGAGAGTATGCCG
AGTCCTTCTTTGGCCAACTTTGTGGACTGTGACGAGTCCAACAGTGACAGTGAAGGAGAG
TTAGAGACCCCGCCTTCTCTGCAGGGAGGTCTCGGCCCCACAACACTCCCACAGCAAAAC
GCTGATCCGGATGTGGATGTACCTCGGTGGGAAGTATTCTTCAAAAGAAAAGATGAAATC
ACAGATGAATGTTTGGAAAACTTACCTTCCTCCATAGAGACAGGGGGATCGCAGTCACCA
AAGCGTTTCAGTGACTCACCAAAGCTGGGCAGTGACTCTGATGGAGAATCCACCCATATC
TCTTCCCAGAATTCATCTCAGTCAACCCACATAACGGATCAAGGAAGCCAAGGCTGGGAC
AGCCAGTGTGATACCGTTTTGTTATCTTCCCAAGAGAAAAGTGGTGGGGATAGCACCTCT
TTGAACAAGGATACCTACAAACCAAAACCCAAAGATAGTATTTCTGCCTCCCAAATAGAA
CAGAATGCACTTTGTCCACAGGATACTCACTGTGATTTGAAAAGCGGAGCTGAAGTAAAT
GGAGTCCCTTGTATTGAAGAACCAGACACTGTGAGTGGCAGGAAATCTTCACCTGAGAAA
ACATCGCTAACCAGCACACAGGCAGACTCACAGAGCTCCTCTGACTTTGAAATCCCCTCA
ACTCCAGAAGCTGAGCTTCCCAAGCCAGAGCATTTACAGTTCTTATATGGAAAGCTGGCA
ACAGGTGAGAGTATAGTGTTGAAAAAAGAAAATGTTCACTCCCAGATATTTAAATAA
```

<u>Alignment with the reference sequence</u>

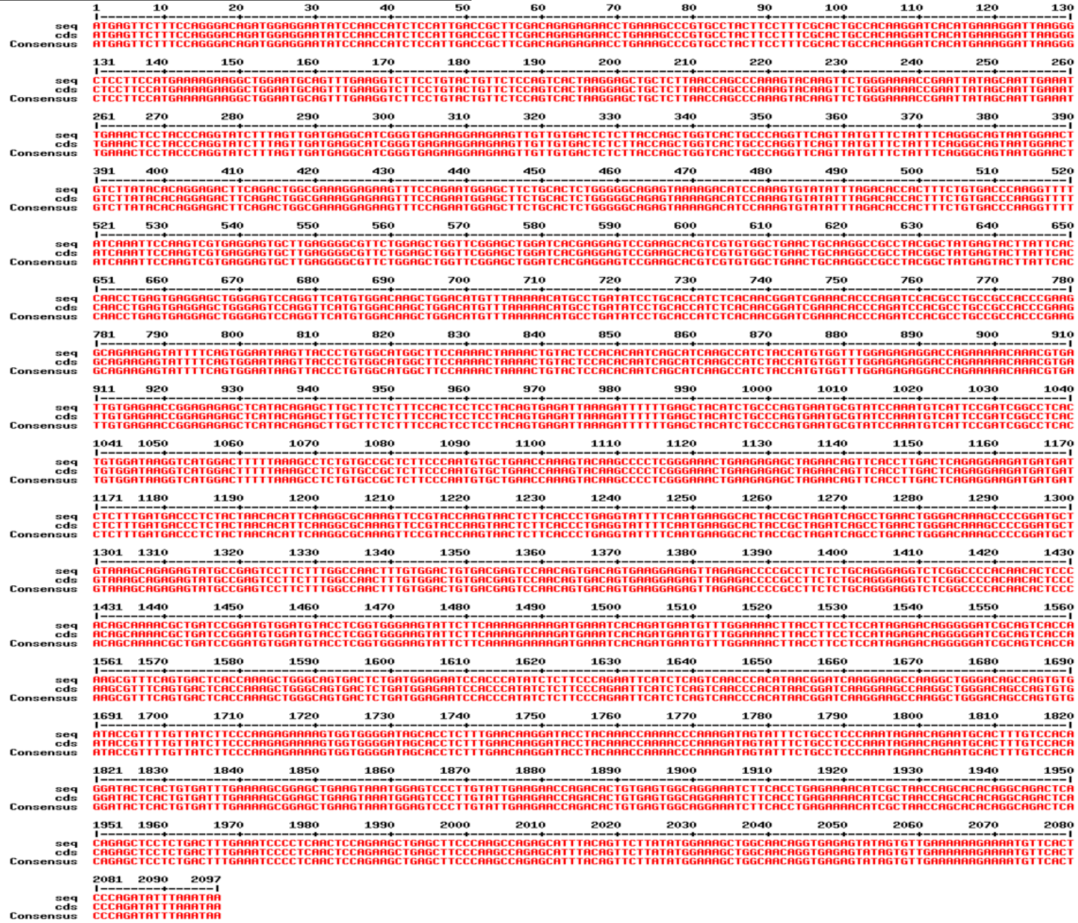

## 6. Identification of *Dntt (TDT)* mRNA from rat spleen and astrocytes (DI TNC1, cortex)

```
ATGGATCCGCTGCAAGCAGTCCACCTGGGTCCTCGGAAGAAGAGACCGAGGCAGACGGGC
GCCTCAGGGGCCTCCACACCTCACGACATCCGATTTCGAGACTTGGTCCTCTTCATTTTG
GAGAAGAAGATGGGAACAACTCGAAGAGCCTTCCTCATGGAGCTGGCCCGAAGGAAAGGG
TTCAGGGTGGAAAATGAGCTCAGTGACTCTGTCACCCACATCGTGGCAGAGAACAACTCG
GGATCCGACGTCCTGGAGTGGCTCCAGCTACAGAACATCAAAGCCAGCTCTGAGTTCGAA
CTCTTGGACATCTCCTGGCTGATTGAATGCATGGGAGCTGGGAAACCGGTGGAGATGGCG
GGGAGACATCAGCTCGTTGTGAGAAGAAACCCTTCCCTGAGTCCTGTCCCAGGCTCCCAG
ACCGTTCCGCCACCTCCCATGCAGAAGATCTCCCAGTACGCTTGTCAGCGGAGAACCACA
TTGAACAATCACAACCAACTATTCACGGATGCGTTTGATATCCTGGCTGAAAATTATGAG
TTTAGAGAGAATGAAGTTTCTTGTCTGCCATTCATGCGAGCTGCCTCTGTACTGAAATCT
CTGTCCTTCCccATCGTCAGCATGAAAGACATAGAGGGAATTCCTTGCCTAGGGGACAAG
GTGAAGTGTGTCATAGAGGGAATTATTGAAGATGGAGAAAGTTCTGAAGTGAAAGCTGTG
TTAAATGATGAGCGATATAAATCCTTCAAACTCTTTACATCTGTGTTTGGCGTGGG
ACTGAAGACAGCTGAGAAATGGTTCAGGATGGGGTTCAGAACTCTCAGCAAAATAAAGT
CAGACAAAAGCCTGAGGTTCACACACATGCAGAAAGCAGGGTTCCTCTACTATGAAGACC
TCGTTAGCTGTGTGAACAGGGCAGAAGCAGAGGCCGTCAGCATGCTAGTTAAGGAGGCAG
TTGTGGCATTTCTTCCAGATGCCTTGGTCACCATGACTGGGGGGTTCCGCAGGGGTAAGA
TGACCGGACATGACGTAGACTTTCTAATTACCAGCCCAGAAGCCACAGAGGAAGAAGAGC
AGCAGCTCTTGCATAAAGTAACAAACTTTTGGAGACAGCAGGGGTTGCTTTTGTACTGCG
ACATCATAGAGTCGACCTTTGAAAAGTTCAAGCTGCCCAGCAGGAAGGTGGACGCCCTCG
ATCACTTCCAGAAGTGCTTCCTTATTCTGAAGCTGCACCGTGGCCTAGTGCGCAGTGAGG
AGAGCGGCCAGCAGGAAGGAAAGGACTGGAAGGCCATCCGTGTAGATCTGGTCATGTGCC
CCTATGAGCGCCGGGCCTTCGCCCTGCTCGGGTGGACTGGATCCAGGCAGTTTGAGAGAG
ACTTGCGGCGCTACGCCACACACGAGCGGAAGATGATGCTGGACAACCACGCTCTGTATG
ACAAGACCAAGAGGGTGTTTCTTGAAGCAGAAAGTGAAGAAGAGATCTTTGCCCATCTGG
GATTGGACTACATTGAACCATGGGAAAGAAATGCTTAA
```

<u>Alignment with the reference sequence</u>

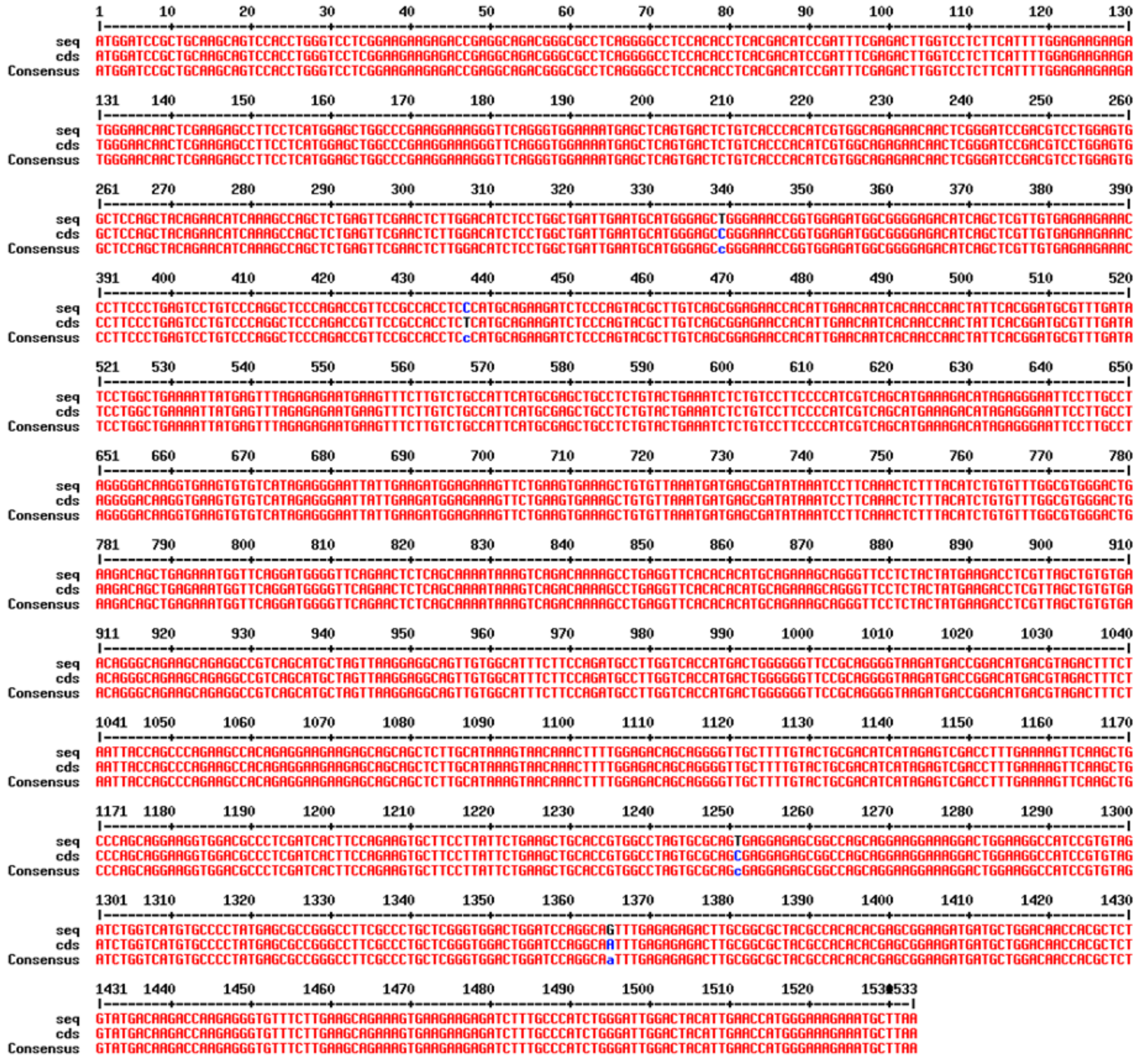

## 7. Identification of *Lig4* mRNA from rat spleen and astrocytes (DI TNC1, spinal cord)

ATGGCTTCCTCACAAGCCTCACGAACTGTCGCAGCTCATGTCCCCTTTGCAGACTTGTGC
TCCACCTTAGAACGAATACAGAAAGGTAAAGACCGTGCAGAAAAAATCAGGCACTTCAAG
GAGTTTCTGGATTCTTGGAGAAAATTTCATGATGCCCTTCATAAGAACAAGAAGGATGTT
CCGGACTCTTTTTACCCAGCAATGAGACTTATTCTCCCTCAGCTAGAGAGAGAGAGGATG
GCTTACGGAATCAAAGAAACCATGCTGGCTAAGCTTTACATCGAATTGCTCAATTTACCA
AGAGAAGGCAAGGATGCCCAGAAGCTCCTGAATTACCGAACCCCCAGTGGAGCTCGCTCA
GATGCTGGGGACTTTGCCACGATTGCATACTTTGTTTTGAAGCCAAGGTGCTTACAGAAA
GGAAGCTTAACCATACAGAAGGTGAATGAACTCTTAGACTTAGTTGCCAGCAATAACTCT
GGCAAAAGAAAAGATCTAGTGAAAAAGAGCCTTCTTCAGTTAATAACCCAGTGTTCAGCA
CTCGAGCAAAAGTGGCTGATTCGCATGATTATTAAGGACTTAAAGCTTGGTGTCAGTCAG
CACACGATATTGTCCATCTTCCACAATGATGCAGTCGAGTTGCACAATGTCACCACAGAT
CTGGAAAAGGtCTGTAGGCAGCTACATGACCCCTCCGTAGGGCTCAGCGACATCTCCATC
ACTTTGTTCTCTGCCTTTAGGCCAATGCTAGCTGCTGTAGCAGATGTGGAGCGTGTGGAG
AAGGACATGAAGCAGCAGAGTTTCTACATCGAAACTAAGCTCGATGGCGAGCGCATGCAG
ATGCACAAGGATGGCGCCCTGTACCGGTACTTCTCCAGAAATGGTTACAACTACACCGAT
CAGTTCGGCGAATCTCCACAGGAAGGCTCTCTCACCCCGTTTATTCACAACGCATTCAAG
ACAGATGTGCAAGTGTGCATCCTTGATGGCGAGATGATGGCCTACAACCCAACGACACAG
ACTTTCATGCAGAAGGGGGTCAAGTTTGATATCAAAAGGATGGTCGAAGATTCTGACCTA
CAGACATGCTACTGTGTTTTCGATGTGCTGATGGTTAATAATAAGAAGCTAGGGCGTGAG
ACTCTCAGGAAGAGGTATGATATCCTTAGTAGCACTTTCACACCCATACAAGGTCGAATA
GAGATAGTGCAGAAAACTCAAGCTCAGACAAAGCAGGAAGTAGTGGATGCATTAAATGAT
GCCATAGACAAGAGAGAAGAGGGGaTCATGGTTAAACACCCTCTGTCCATTTACAAGCCA
GACAAAAGAGGTGAAGGGTGGCTAAAGATTAAACCAGAGTATGTCACTGGATTGATGGAT
GAATTAGACCTCTTAATTGTTGGGGGCTACTGGGGTAAAGGTTCTCGAGGTGGCATGATG
TCTCACTTTCTGTGTGCAGTGGCAGAGACACCACCTCCTGGTGACAAGCCATCTGTGTTC
CACACTCTGTGCCGTGTTGGGTCGGGTTACACGATGAAAGAACTCTATGACCTGGGCTTG
AAATTGGCAAAATACTGGAAGCCTTTCCAAAGAAAATCTCCACCAAGCTGTATTTTGTGT
GGCACAGAGAAGCCAGAAGTGTACATTGAACCCCAGAACTCTGTCATCGTTCAGATCAAG
GCGGCAGAGATCGTCCCCAGTGACATGTACAAGACTGGCTCTACCTTGCGCTTCCCACGC
ATCGAGAAGATCAGAGATGACAAAGAGTGGCATGACTGTATGACGCTGGATGACCTGGAG
CAGCTGAGGGGGAAAGCATCTGGGAAGCTTGCCACAAAACACCTTCACATAGGTGACGAT
GACGAACCCAGAGAAAAAAGGCGGAAACCCATCTCTAAGACGAAGAAAACCATTGGAATC
ATTGAACACCTAAAAGCGCCTAACCTTTCTAACATAAACAAAGTTTCTAATGTATTTGAA
GATGTTGAGTTTtGTGTCATGAGTGGATTAGATGGTTATCCAAAGCCTGACCTAGAGAAC
AAAATTGCAGAATTTGGTGGTTATATAGTCCAGAATCCAGGCCCAGATACATACTGTGTG
ATTGCAGGTTCTGAGAACATTAGGGTCAGAAACATCATCTCTTCAGATAGGCATGATGTT
GTCAAGCCCGAGTGGCTTTTAGAGTGTTTTAAAACAAAAACATGTGTGCCATGGCAACCC
CGCTTTATGATTCACATGTGCCCATCAACAAAGCAGCATTTTGCCCGTGAATATGACTGC
TATGGTGATAGCTATTTCGTTGATGCAGATTTGGATCAATTGAAAGAAGTGTTTTTAAGA
ATTAAACCCAGTGAGCAGCAGACTCCTGAAGAAATGGCCCCTGTGATTGCTGACCTAGAA
TGTCGTTATTCCTGGGATCACTCTCCTCTCAGTATGTTTCGACACTACACCGTTTATTTG
GACTTGTATGCTGTTATTAATGACTTAAGTTCCAAAATCGAAGCCACAAGGTTAGGTGTT
ACAGCACTTGAGCTGCGGTTTCGTGGAGCAAAGGTGGTTTCCCGCTTATCTGAAGGGGTG
TCTCACGTAATCATCGGGGAAGATCAGAGACGAGTCAGTGACTTTAAAACGTTCAGAAGA
ACGCTTAAGAAAAAGTTTAAAATCCTGCAGGAACGTTGGGTGATCGATTCAGTAGACAAG
TGTGAACTGCAGGAAGAAAACCTGTATTTGCTTTAG

Alignment with the reference sequence

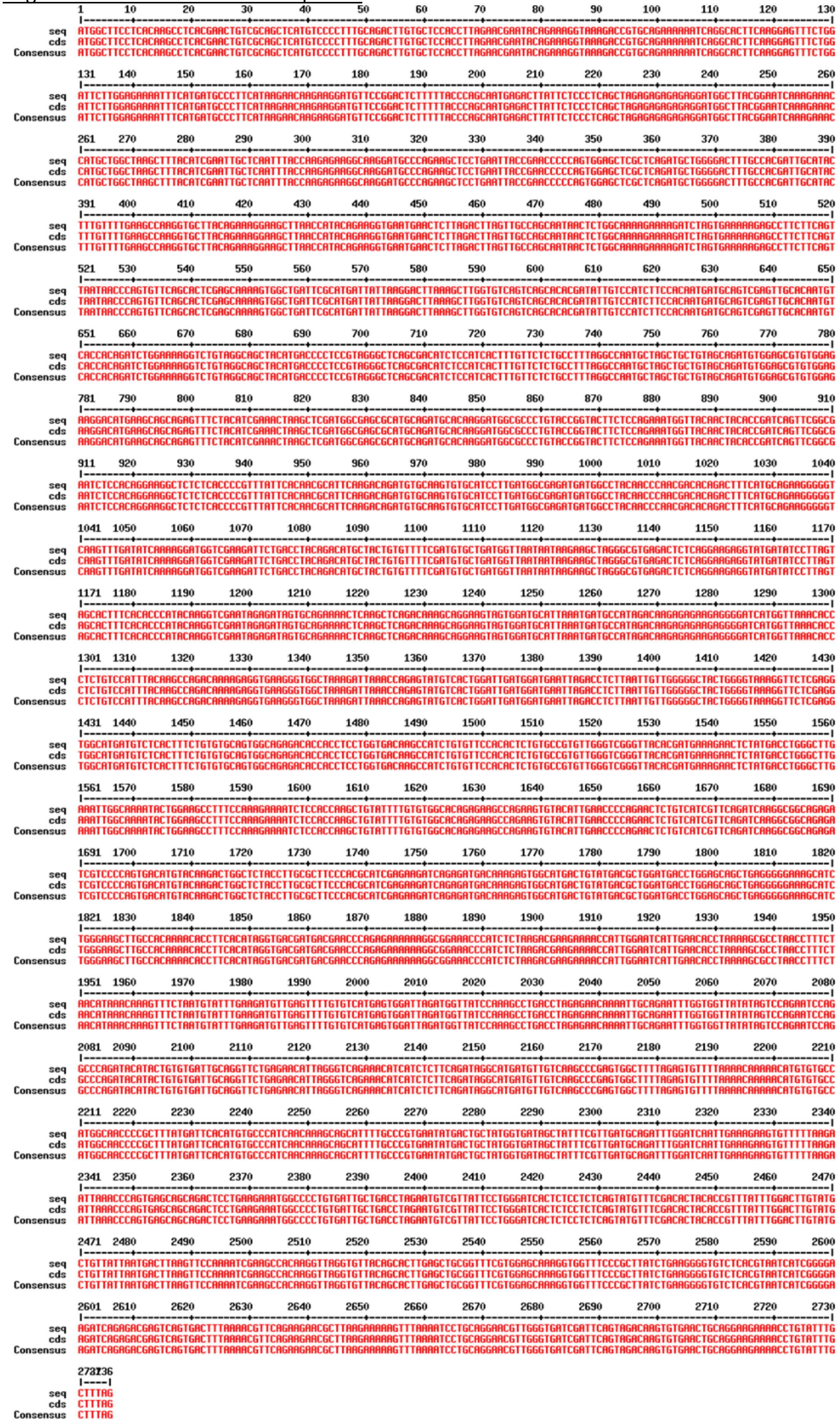

## 8. Identification of *Nhej1* mRNA from rat astrocytes (DI TNC1, spinal cord and cortex)

```
ATGGAGGAACTAGAGCAAGGCCTGTTGATGCAGCCATGGGCATGGTTACAACTTGCTGAG
AACTCACTCTTGGCCAAGGCTTCTATCACCAAGCATGGTTATGCCTTGCTGATTTCGGAT
CTTCAACAGGTGTGGCATGAACAGGTGGACACTTTGGAGGTCAGCCAGCGAGCCAAGGAG
CTGAACAAGCGCCTCACCGCTCCCCCTGCAGCTTTTCTCCATCACTTGGATGAAGTGCTT
CGCCCACTGTTTAAAGATTCTGCCCACCAGGATGCTGCTCACCCTAGCAAAGCCACTTTC
TCCTGTGATCGTGGAGAGGAGGTATTGATCCTGAGGGTGCGGAGTGAGCTCTCTGGTCTC
CCCTTCAATTGGCATTTCCACTGTCTTCCAGCTAGTTCTTTACTGGTCTCTCAGCATTTG
ATTTGTCCTCTGATGGGTGTGAGCTTGGCATTGCACAGTCATGTGAGGGAGCTAGCAGCA
TTGCTTCGGATGAAGGACCTTGAGATCCAGGCCTACCAGGAGAGTGGGGCTGTGCTGAGC
CGAGGTCGATTGAAGACAGAGCCATTTGAAGAAAATTCTTTCTTGGAACAGTTTATGGTA
GAGAAATTGCCAGAGGCATGTGCTGTTGGTGATGGAAGGCCATTTGCCATGAATCTGCAG
AGCCTGTATGTGGCAGTCACAAAGCAGCAGGTCCAAGCCAGGCAGAAGCATAAAGGCTCT
GGAGAGCCTCAGACATCAAGCAGCACCTCCCCTCAAGGAACTGATAGCCAGCTTCAGAAC
CAGCCAGAACAGCAGATCTCTCCCACTCCAACCCTCTCAGAGCCTGAATGTGAGCCTATG
GCTGCTTCAGGCCCTGTGCATAGAGCTCAGCTGGTAAAGGCCAAGAGGAAGAAGCCCAGG
GGACTCTTCAGTTAG
```

Alignment with the reference sequence

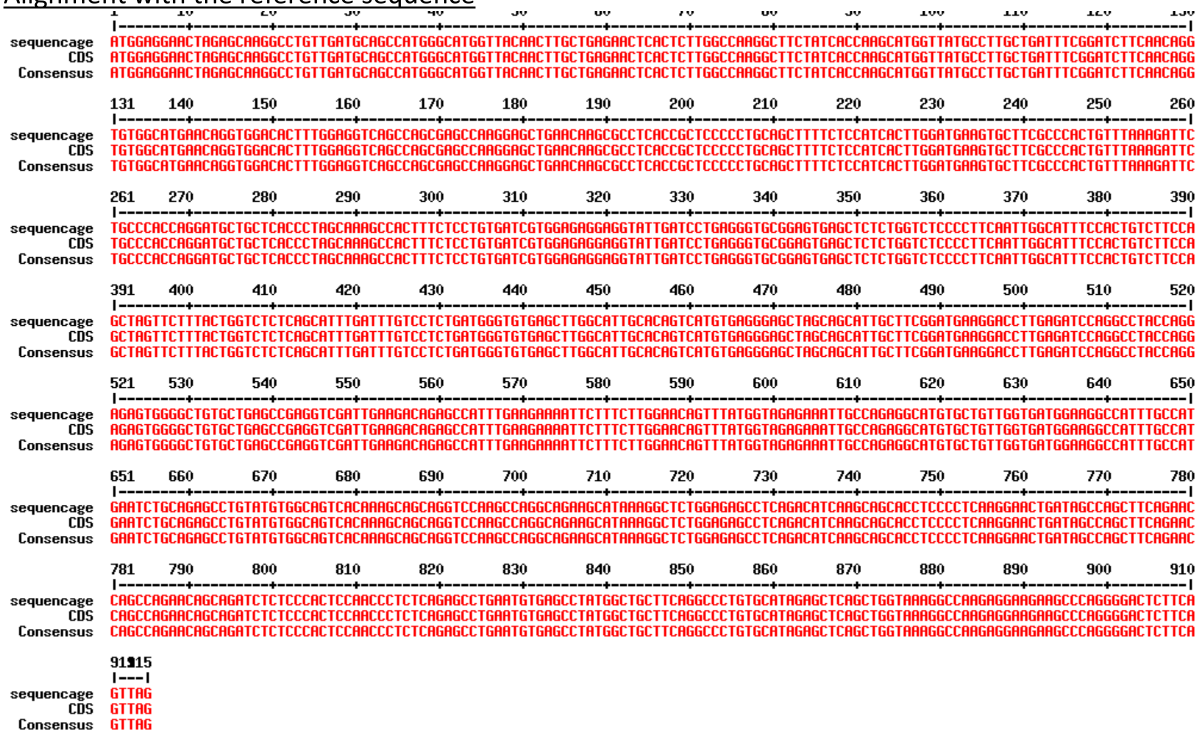

## 9. Identification of *RAG1* mRNA from rat astrocytes (cortex)

ATGGCTGTCCCCTTGCCATCTACCCTGAGACTCAGTTCTGCACCTGATGAAATTCAGCAC
CCGCACATCAAATTTTCCGAGTGGAAATTTAAGCTGTTTAGGGTGAGATCCTTTGAAAAG
GCACCCGAAGAAGCACAAAAAGAGAAGGATTCCTCAGAAGGGAAACCTTGTCTCGAGCAG
TCTCCAGTAGTTCTAGATAAGCCTGGGGGTCAGAATTCAGTTCTGACTCAACGAGCATTG
AAACTCCATCCTAAATTTTCAAAGAAATTCCATGTTGATGGGAAGTCAAGCGACAAAGCA
ATTCACCAAGCCAGGCTTAGACACTTCTGCCGCATCTGTGGCAATCACTTCAAGAGTGAC
GGGCACAACCGGAGATACCCAGTCCACGGGCCCGTGGACGCTAAAACTCAAAGCCTTTTC
CGAAAGAAGGAAAAAAGAGTCACGTCCTGGCCAGATCTCATTGCCAGAGTTTTCCGGATT
GATGTGAAGTCAGATGTTGACTCCATCCACCCCACTGAATTCTGCCATAACTGTTGGAGC
ATTATGCACAGGAAGTTCGGCAGTGCTCACAGTCAGGTCTACTGCCCAAGGAATGTGACC
GTGGAGTGGCACCCCCACACACCGTCCTGTGACATCTGCTTTACTGCCCATCGGGGACTG
AAGAGGAAGAGACATCAGCCCAACGTGCAGCTCAgCAAGAAACTAAAAACTGtGCTCAAC
CATGCTAGACGGGACCGTCGCAAGAGAACTCAGGCTAGGGTCAGCAGCAAGGAGGTCATG
AAGAAGATCTCCAACTGCAGTAAGATTCATCTCAGTACCAAGCTTCTTGCAGTAGACTTC
CCAGCACACTTTGTGAAATCCATCTCCTGCCAGATATGTGAACACATTCTGGCCGATCCG
GTGGAGACCACCTGTAAGCACCTGTTCTGTAGAATATGCATTCTCCGGTGCCTCAAGGTC
ATGGGCAGCTATTGTCCCTCGTGCCGATATCCATGCTTCCCTACTGACCTGCAGAGTCCG
GTCAAGTCCTTTCTGAGCATCTTGAATTCTCTGATGGTGAAGTGTCCTGCCCAAGAGTGC
AATGAGGAAGTAAGTCTGGAAAAATATAACCACCATGTCTCAAGCCACAAAGAATCTAAA
GAGACTTTGGTGCATATTAATAAAGGGGGCCGGCCTCGCCAGCATCTCCTGTCACTGACG
CGAAGGGCTCAGAAACATCGGCTGAGGGAGCTCAAGATTCAAGTCAAAGAATTTGCAGAC
AAAGAAGAAGGTGGAGATGTGAAGTCTGTGTGCTTGACGTTGTTCCTGCTGGCACTGAGG
GCAAGGAATGAGCACAGACAAGCTGATGAGCTCGAGGCCATCATGCAAGGCCGGGGCTCC
GGTCTTCAACCAGCTGTTTGCTTGGCCATCCGTGTCAATACCTTCCTCAGCTGTAGCCAA
TACCATAAGATGTACAGGACGGTGAAAGCTATCACTGGGAGGCAGATTTTTCAACCTTTG
CACGCTCTTCGCAATGCTGAGAAGGCCCTCCTACCAGGCTACCACCCCTTTGAGTGGCAG
CCCCCTCTGAAGAACGTGTCCTCCAGAACTGACGTTGGAATTATTGATGGGCTCTCTGGA
CTTGCCTCCTCCGTAGATGAATACCCAGTGGACACAATTGCAAAGAGGTTCCGCTATGAC
TCTGCTTTGGTGTCTGCTTTGATGGACATGGAGGAAGACATCTTGGACGGCATGAGATCC
CAAGATCTTGATGACTACGTGAGTGGTCCCTTCACCGTGGTGGTAAAGGAATCGTGTGAT
GGAATGGGGGATGTGAGTGAGAAGCACGGGAGTGGGCCCGCAGTTCCAGAAAAGGCAGT
TCGGTTCTCTTTCACCGTCATGAGAGTTACAGTCGAGCATGGCTCTCAGAACGTGAAGAT
ATTTGAGGAACCCAAGCCCAATTCTGAACTGTGTTGCAAGCCACTGTGCCTTATGCTGG
CAGATGAGTCTGACCACGAGACACTTACTGCTATTCTCAGCCCCCTCATCGCTGAGAGGG
AGGCCATGAAGAGCAGTGAATTGCTGCTGGAGATGGGAGGCATCCCCAGGACTTTTAAAT
TTATCTTCAGGGGCACTGGATATGACGAGAAACTTGTCAGGGAAGTAGAAGGCTTGGAAG
CTTCTGGCTCAGTCTACATTTGTACACTTTGTGATGCCACCCGTCTGGAAGCCTCCCAAA
ATCTTGTCTTCCACTCCATAACCAGAAGCCACGCCGAGAATCTGCAGCGCTATGAGGTCT
GGCGGTCCAATCCCTATCACGAGTCAGTGGAAGAACTCCGGGACCGGGTGAAAGGAGTCT
CTGCCAAACCTTTCATCGAGACAGTCCCTTCCATAGATGCGCTTCACTGTGACATTGGAA
ATGCAGCTGAATTCTATAAGATTTTCCAGCTGGAGATAGGGGAAGTGTATAAAAATCCCA
ATGCCTCTAAAGAGGAAAGGAAGAGATGGCAGGCCACCTTGGACAAACATCTGCGGAAAA
GGATGAACTTAAAACCAATCATGAGGATGAATGGCAACTTTGCCCGGAAGCTTATGACCC
AAGAGACTGTGGACGCAGTTTGTGAGTTAATTCCTTCTGAGGAGAGGCacGAAGCTCTCA
GGGAGcTGATGGACCTTTACCTGAAGATGAAACCGGTGTGGCGCTCATCATGTCCTGCTA
AAGAGTGCCCGGAGTCCCTTTGTCAGTACAGTTTCAACTCACAGCGTTTCGCTGAGCTCC
TCTCCACCAAGTTCAAATACAGATACGAGGGCAAAATCACCAATTACTTTCACAAAACCT
TGGCACACGTCCCTGAAATTATTGAAAGGGATGGCTCTATTGGGGCATGGGCAAGTGAGG
GCAATGAATCTGGTAACAAACTGTTTAGGCGCTTCCGGAAAATGAATGCCAGGCAGTCCA
AGTGCTATGAGATGGAAGATGTCCTGAAACACCACTGGCTGTATACTTCAAAATACCTCC
AGAAGTTTATGAATGCCCATAATGCATTAAAAAACTCTGGGTTTACCATGAACTCAAAGG
AGACTTTAGGGGACTCTTTGGACATTGAGGACTCTCTGGAAAGCCAAGATTCAAtGGAGT
TTTAA

Alignment with the reference sequence

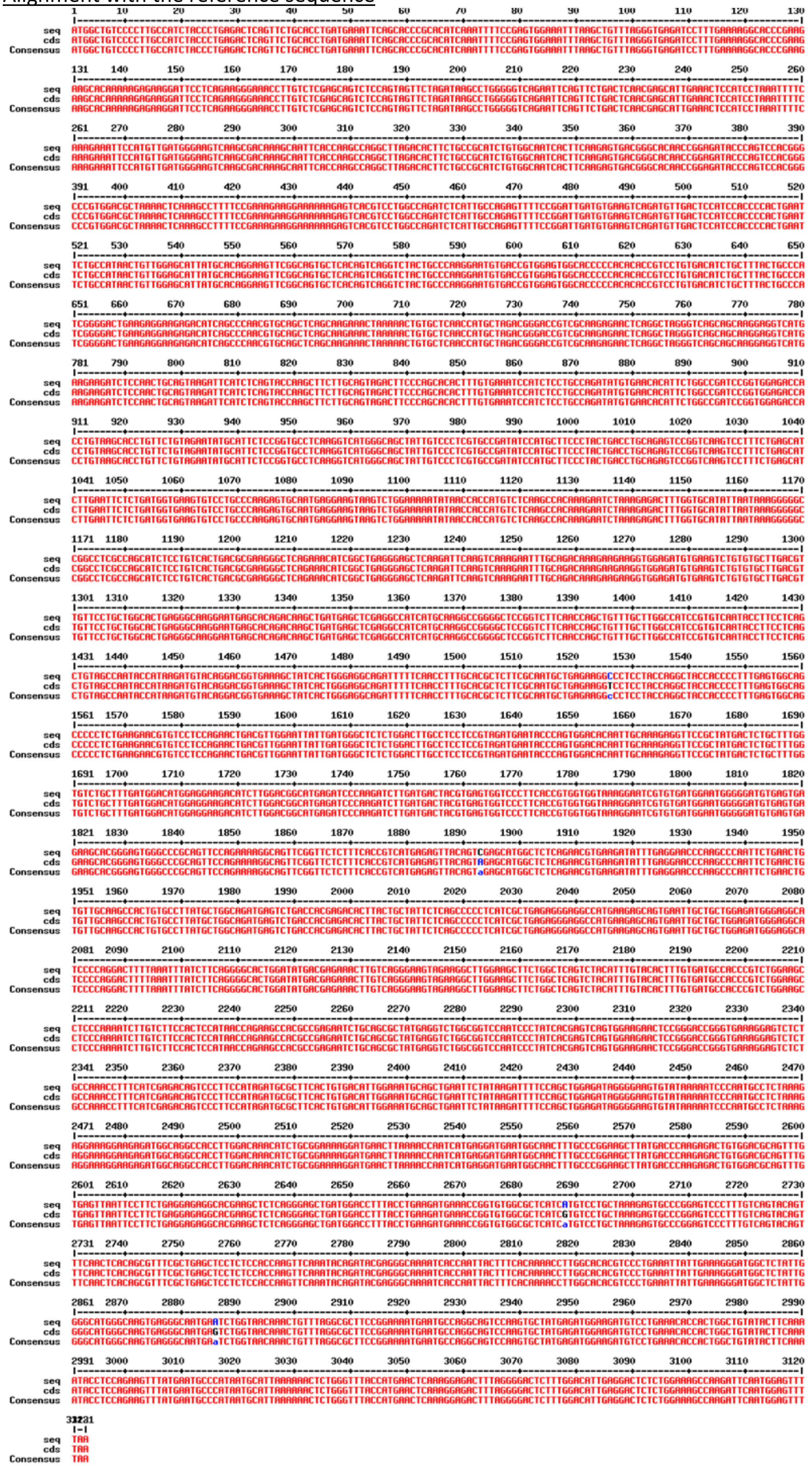

## 10. Identification of *RAG2* mRNA from rat astrocytes (DI TNC1, cortex)

ATGTCCCTGCAGATGGTTACAGTGGGTCATAACATAGCCTTAATTCAACCAGGCTTCTCA
CTGATGAATTTTGATGGTCAAGTTTTTTTCTTTGGCCAAAAAGGCTGGCCTAAGAGATCC
TGCCCTACTGGAGTCTTTCATTTTGATATAAAACAAAATCATCTCAAACTGAAGCCTGCA
GTTTTCTCTAAAGATTCCTGCTACCTCCCACCTCTTCGTTACCCAGCTACTTGCTCGTAC
AAAGGCAGCACAGACTCTGACAAGCATCAATATATCATCCACGGAGGGAAAACGCCAAAC
AATGAGCTTTCCGATAAGATTTATATCATGTCTGTCGCTTGCAAGAATAACAAAAAAGTT
ACTTTCCGTTGTACAGAGAAAGACTTAGTAGGAGATGTCCCTGAAGCCAGATATGGCCAT
TCCATTGACGTGGTATATAGCCGAGGAAAAAGTGTTGGTGTTCTCTTTGGAGGACGGTCA
TACATGCCTTCTACCCAAAGAACCACAGAAAAATGGAATAGTGTAGCTGATTGCCTACCC
CATGTTTTCTTGGTAGATTTTGAATTTGGGTGTGCAACGTCATATATTCTCCCAGAACTT
CAGGACGGGCTGTCTTTTCATGTTTCTATTGCCAGAAATGATACCATTTATATTTTGGGA
GGACACTCACTTGCCAGTAACATACGTCCTGCTAACTTGTATAGAATAAGAGTAGATCTT
CCCCTGGGTACCCCAGCAGTGAATTGCACTGTCTTGCCAGGAGGAATCTCTGTCTCCAGT
GCAATTGTCACTCAAACAAATAATGATGAATTTGTTATTGTNGGTGGTTATCAGCTGGA
AAATCAAAAAAGGATGGTGTGTAGCATTGTCTCTCTAGGGGACAACAGGATTGAAATTAG
TGAGATGGAGACCCCAGACTGGACCCCAGATATTAAGCATAGCAAAATATGGTTTGGAAG
CAACATGGGAAACGGGACTGTTTTCCTNGGCATACCAGGAGACAACAAGCAGGNTATGTC
AGAAGCATTCTATTTCTATATGTTGAGATGCTCTGAAGATAATTCGAGTGAGGAGCAGAA
AATTGTCTCAAACAGTCAGACATCAACTGAAGATGCCGGGGACTCCACTCCCTTTGAAGA
CTCGGAAGAATTTTGTTTCAGTGCTGAAGCAATCAGCTTTGATGGTGATGATGAATTTGA
CACTTACAATGAAGATGATGAAGATGATGAATCTGTAACCGGCTACTGGATAACGTGTTC
CCCTACTTGTGATGTGGACATCAATACTTGGGTTCCATTCTATTCAACTGAGCTCAATAA
ACCCGCCATGATCTATTGTTCTCATGGGGATGGACACTGGGTGCATGCCCAGTGCATGGA
TCTGGAAGAACGCACACTCATCCACTTGTCAGAAGGAAGCAACAAATATTACTGCAATGA
GCATGTGCGGATAGCAAGAGCATTACAAACTCCCAAAAGAAACCTCCCCTTACAAAAGCC
CCCAATGAAATCCCTCCACAAAAAAGGCTCCGGGAAAGTCTTGACTCCTGCCAAGAAATC
CTTTCTTAGGAGGTTGTTTGATTAG

Alignment with the reference sequence

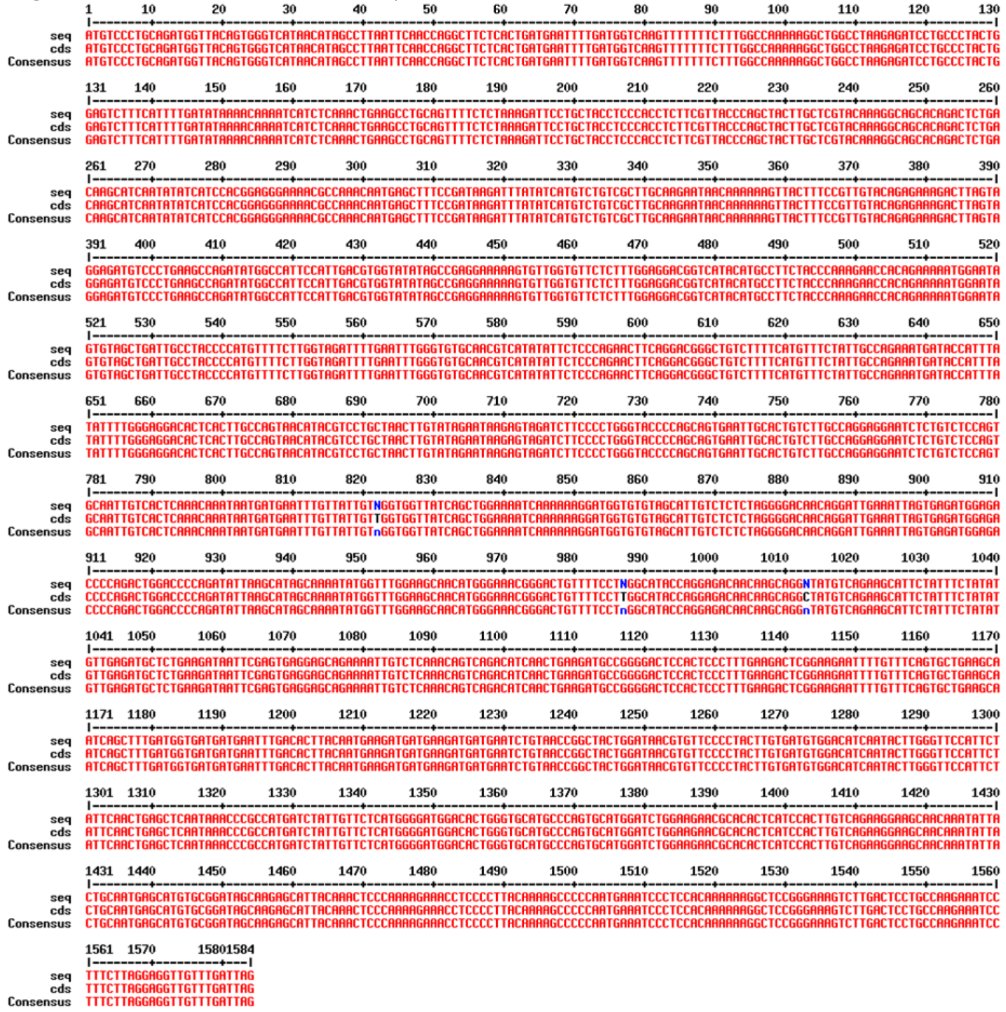

## 11. Identification of *Xrcc4* mRNA from rat astrocytes (DI TNC1, spinal cord, cortex)

ATGGAAAGGAAAGTCAGC
AGAATCTCTCTGGCTTCTGAACCCAATGTAACCTACTTTCTACAAGTGTCTTGGGAGGGT
ACGATTGGATCTGGTTTTGTTATTACACTTACGGATGGCCATTCGGCCTGGACTGCAAAA
GTTTCTGAATCGGAGATTGCACAGGAAGCTGATGACATGGCTATGGAGCAGGAAAAATAC
ATGGATGAGCTGAGACGGCACTGGTGCCAGAGTCGGGAGCAGCTGGTGCATACAAGTTC
ATCTTTTCCAAGGACACTCAGCATTTCTCCCTGGAGAAAGAACTGAAAGATGTTTCTTTC
AGACTTGGTTCCTTCAACCTAGACAAAGTTACAAACCCAACTGAAGTCATCAGAGAACTC
ATTTGCTACTGCCTGGACACCATTGCGGAAAAACAAGCCAAAAACGAGCACCTGCAGAAG
GAAAACGAAAGGCTCCTGAGAGATTGGACTGACGTTCAAGGCCGATTTGAGAAATGTGTG
AGTGACAAAGAAGCCTTGGAGGCCGATTTGTATCAAAGATTTATCTTGGTGCTGAATGAG
AAGAAAACAAAAATCCGAAGCTTGTTGAGTGAAGTCCAGCAGCTGGAGAAGAGTACCAAA
CCTGAAAGGGAAACTCCGAGTTCTGACAAAATACCTGAGGAGGATGGGCTTTATGATGGC
AGCACTGATGAAGAAAGCGGAGCCCCTGTGCTGGGCGCAGATACCCTTGATAAGGATGAT
TCCTTATTTTCAAGTCCAGATGTCACTGATATTGCACCAAGTAGGAAGAGGAGACACCGA
ATGCAGACAAATCTTGGGACAGAACCTAAAATGGCTCCACAGGAGCTGCCACTACAGGAG
AAGGAAAGGCTTGGCTCTTCACCGCCTCAGACCTTGAAAGAGGAGGGCACCTCTGCTGCA
AACGTGTCCTTAGAAACCCTGAGAAACAGCAGCCCAGAAGaCCTCTTTGACTAG

Alignment with the reference sequence

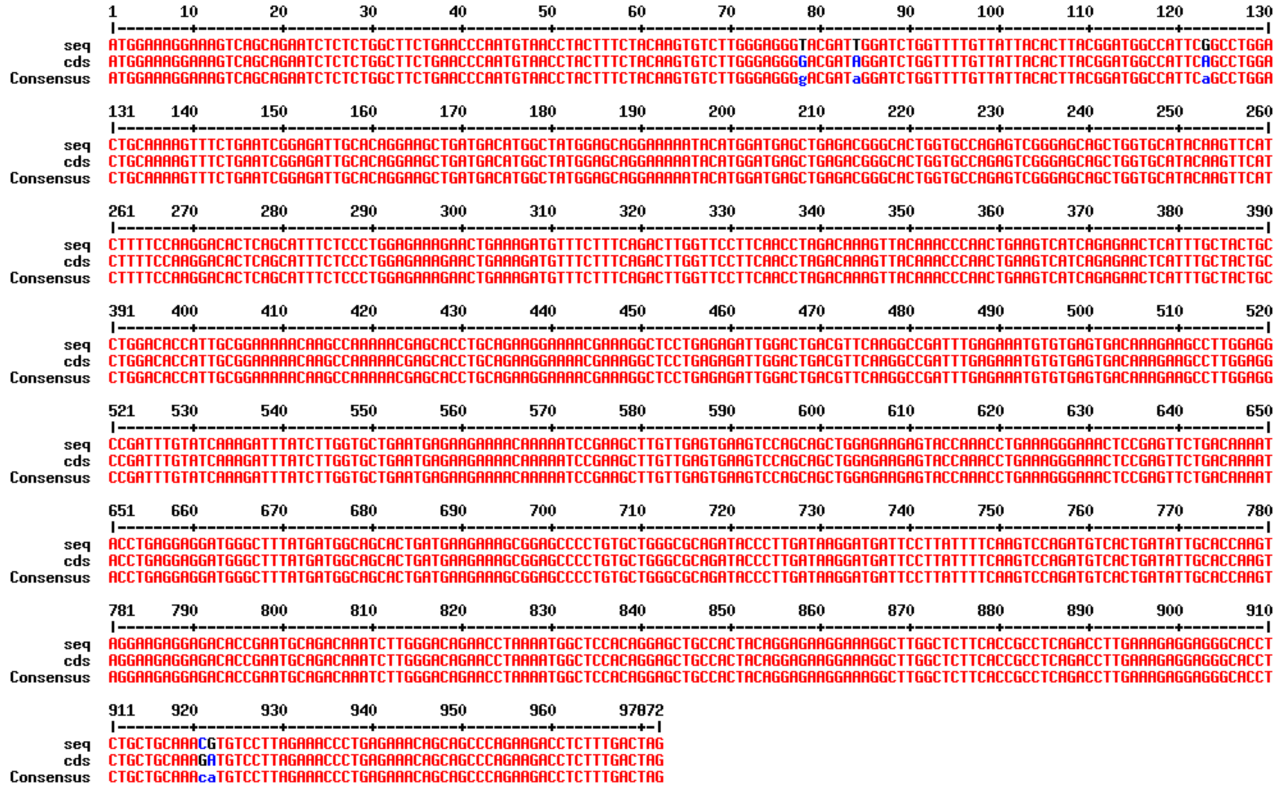

### 12. Identification of *Xrcc5* mRNA from rat astrocytes (DI TNC1, spinal cord, cortex)

```
ATGGCGTGGTCCGGTAATAAGGCAGCTGTTGTGCTGTGTATGGATGTGGGGGTTGCCATG
GGTAACTCCTTTCCTGGTGAAGAATCCCCACTTGAACAGGCCAAGAAAGTGATGACTATG
TTTGTCCAACGACAGGTGTTTTCGGAGAGCAAGGATGAGATCGCCTTAGTCCTCTATGGT
ACAGAGAGCACTGATAACGCCCTTGCTGGCAAGGACCAGTATCAGAACATCACAGTGCAC
AGGCACCTGATGCTACCAGATTTTGATTTGCTGGAAGACATCGGAAACAAAATCCAACCA
AGTTCTCAACAAGCCGACTTCCTGGACGCCCTGATTGTGTGCATGGATTTGATTCAGCGT
GAAACCATAGGAAAGAAGTTTGGGAAGAAGCATATTGAAGTGTTCACTGATCTCAGCAGC
CCATTCAGCCAAGACCAACTGGACATTATAATTTGTAACCTGAAGAAGTCCGGCATCTCC
CTGCAGTTCTTTCTGCCTTTTCCAATCGACAAGAATGGTGAGCCTGGGGAGACAGAAGAC
CACGACTCCAGCTTCGACCACTGTGCACCCTCCTTCCCTCAAAAAGGACTTACTGAGCAG
CAAAAGGAAGGCATCCACATGGTGACGAGGGTGATGCTGTCTTTAGAAGGCAAAGATGGT
CTGGATGAAATTTATTCCTTCAGCGAGAGTCTTCAGCAACTGTGCATCTTTAAGAAGATT
GAGAGGCGGTCCTTGCCCTGGCCCTGCCAACTGACCATCGGCCCCAACTTGTCTATAAGG
ATCGTAGCCTATAAATCGATTGTACAGGAGAAATTTAAAAAGAGTTGGGTAGTTGTGGAC
GCAAGAACTCTAAAGAAGGAAGATATACAAAAAGAGACTGTTTATTGCCTAAATGACGAT
GATGAAACTGAAGTTTCCAAAGAGGACACTATTCAAGGGTTCCGCTATGGAAGCGACATC
ATTCCTTTTTCTAAAGTGGATGAGGAACAAATGAAATATAAATCGGAGGGGAAGTGCTTC
TCTGTTCTGGGGTTCTGTAAATCTTCTCAGGTTCATAGAAGATTCTTCATGGGACATCAA
GTTCTGAAGGTCTTCGCAGCGAAGGATGATGAGGCAGCAGCTGTTGCACTTTCTTCCCTA
GTTCACGCTTTGGATGAGTTAAACATGGTCGCTATCGTCCGCTACGCCTATGACAAAAGA
GCTAATCCTCAAGTTGGTGTAGCCTTCCCTTTCATTAAGGATGCCTATGAGTGTTTAGTT
TATGTGCAGCTGCCTTTCATGGAAGACTTGCGGCAATACATGTTTTCCTCACTGAAAAAC
AATAAGAAGTGCACCCCCACAGAGGCACAGCTGAGTGCGATCGATGATCTGATTGATTCC
ATGAGCTTGGTAAAGAAAAACGAGGAAGAAGACATCATCGAAGACTTGTTTCCAACCTCC
AAAATTCCAAATCCTGAATTTCAGCGGTTGTACCAGTGTCTGCTGCACAGAGCCTTACAT
CTCCAGGAGCGGCTGCCCCCAATTCAGCAGCACATTTTGAATATGCTGGATCCCCCCACT
GAGATGAAAGCAAAATGTGAGATTCCACTCTCTAAAGTAAAGACCCTTTTCCCTCTGACG
GAAGTCGTTAAGAAAAAGGACCAAGTGACTGCTCAGGACGTTTTCCAAGACAATATTGAA
GAGGGGCCCGCTGCTAAAAAATACAAGACTGAGAAAGAAGAAGGTCACATCAGCATCTCC
AGCCTGGCAGAAGGGAATGTCACCAAGGTTGGAAGTGTGAATCCTGTTGAAAACTTCCGT
GTCCTAGTAAGACAGAAGATTGCCAGCTTTGAGGAAGCGAGTCTCCAGCTAATGAGTCAC
ATTGAACAGTTTTTGGATACCAATGAAACACTGTATTTTATGAAAAGTATGGACTGCATC
AAAGCTCTCCGGGAGGAAGCCATTCAGTTTTCAGAAGAGCAGCGTTTCAACAGTTTCCTG
GAAGGCCTTCGAGAGAAAGTGGAAATTAAACAATTAAATCATTTCTGGGAAATTGTCGTG
CAGGATGGAATTACTCTGATCACCAAAGACGAAAGCCCAGGAAGCTCTGTCACAGCTGAG
GAGGCCACGAAGTTCCTGACCCCCAAAGACAAAGCAAAAGAAGACACAACAGGACTTGAA
GAAGGTGGTGATGTGGATGATTTACTGGACATGATATAG
```

Alignment with the reference sequence

```
          1     10        20        30        40        50        60        70        80        90        100       110       120       130
      seq ATGGCGTGGTCCGGTAATAAGGCAGCTGTTGTGCTGTGTATGGATGTGGGGGTTGCCATGGGTAACTCCTTTCCTGGTGAAGAATCCCCACTTGAACAGGCCAAGAAAGTGATGACTATGTTTGTCCAAC
      cds ATGGCGTGGTCCGGTAATAAGGCAGCTGTTGTGCTGTGTATGGATGTGGGGGTTGCCATGGGTAACTCCTTTCCTGGTGAAGAATCCCCACTTGAACAGGCCAAGAAAGTGATGACTATGTTTGTCCAAC
Consensus ATGGCGTGGTCCGGTAATAAGGCAGCTGTTGTGCTGTGTATGGATGTGGGGGTTGCCATGGGTAACTCCTTTCCTGGTGAAGAATCCCCACTTGAACAGGCCAAGAAAGTGATGACTATGTTTGTCCAAC

          131   140       150       160       170       180       190       200       210       220       230       240       250       260
      seq GACAGGTGTTTTCGGAGAGCAAGGATGAGATCGCCTTAGTCCTCTATGGTACAGAGAGCACTGATAACGCCCTTGCTGGCAAGGACCAGTATCAGAACATCACAGTGCACAGGCACCTGATGCTACCAGA
      cds GACAGGTGTTTTCGGAGAGCAAGGATGAGATCGCCTTAGTCCTCTATGGTACAGAGAGCACTGATAACGCCCTTGCTGGCAAGGACCAGTATCAGAACATCACAGTGCACAGGCACCTGATGCTACCAGA
Consensus GACAGGTGTTTTCGGAGAGCAAGGATGAGATCGCCTTAGTCCTCTATGGTACAGAGAGCACTGATAACGCCCTTGCTGGCAAGGACCAGTATCAGAACATCACAGTGCACAGGCACCTGATGCTACCAGA

          261   270       280       290       300       310       320       330       340       350       360       370       380       390
      seq TTTGATTTGCTGGAAGACATCGGAAACAAAATCCAACCAAGTTCTCAACAAGCCGACTTCCTGGACGCCCTGATTGTGTGCATGGATTTGATTCAGCGTGAAACCATAGGAAAGAAGTTTGGGAAGAAG
      cds TTTGATTTGCTGGAAGACATCGGAAACAAAATCCAACCAAGTTCTCAACAAGCCGACTTCCTGGACGCCCTGATTGTGTGCATGGATTTGATTCAGCGTGAAACCATAGGAAAGAAGTTTGGGAAGAAG
Consensus TTTGATTTGCTGGAAGACATCGGAAACAAAATCCAACCAAGTTCTCAACAAGCCGACTTCCTGGACGCCCTGATTGTGTGCATGGATTTGATTCAGCGTGAAACCATAGGAAAGAAGTTTGGGAAGAAG

          391   400       410       420       430       440       450       460       470       480       490       500       510       520
      seq CATATTGAAGTGTTCACTGATCTCAGCAGCCCATTCAGCCAAGACCAACTGGACATTATAATTTGTAACCTGAAGAAGTCCGGCATCTCCCTGCAGTTCTTTCTGCCTTTTCCAATCGACAAGAATGGTG
      cds CATATTGAAGTGTTCACTGATCTCAGCAGCCCATTCAGCCAAGACCAACTGGACATTATAATTTGTAACCTGAAGAAGTCCGGCATCTCCCTGCAGTTCTTTCTGCCTTTTCCAATCGACAAGAATGGTG
Consensus CATATTGAAGTGTTCACTGATCTCAGCAGCCCATTCAGCCAAGACCAACTGGACATTATAATTTGTAACCTGAAGAAGTCCGGCATCTCCCTGCAGTTCTTTCTGCCTTTTCCAATCGACAAGAATGGTG

          521   530       540       550       560       570       580       590       600       610       620       630       640       650
      seq AGCCTGGGGAGACAGAAGACCACGACTCCAGCTTCGACCACTGTGCACCCTCCTTCCCTCAAAAAGGACTTACTGAGCAGCAAAGGAAGGCATCCACATGGTGACGAGGGTGATGCTGTCTTTAGAAGG
      cds AGCCTGGGGAGACAGAAGACCACGACTCCAGCTTCGACCACTGTGCACCCTCCTTCCCTCAAAAAGGACTTACTGAGCAGCAAAGGAAGGCATCCACATGGTGACGAGGGTGATGCTGTCTTTAGAAGG
Consensus AGCCTGGGGAGACAGAAGACCACGACTCCAGCTTCGACCACTGTGCACCCTCCTTCCCTCAAAAAGGACTTACTGAGCAGCAAAGGAAGGCATCCACATGGTGACGAGGGTGATGCTGTCTTTAGAAGG

          651   660       670       680       690       700       710       720       730       740       750       760       770       780
      seq CAAAGATGGTCTGGATGAAATTTATTCCTTCAGCGAGAGTCTTCAGCAACTGTGCATCTTTAAGAAGATTGAGAGGCGGTCCTTGCCCTGGCCCTGCCAACTGACCATCGGCCCCAACTTGTCTATAAGG
      cds CAAAGATGGTCTGGATGAAATTTATTCCTTCAGCGAGAGTCTTCAGCAACTGTGCATCTTTAAGAAGATTGAGAGGCGGTCCTTGCCCTGGCCCTGCCAACTGACCATCGGCCCCAACTTGTCTATAAGG
Consensus CAAAGATGGTCTGGATGAAATTTATTCCTTCAGCGAGAGTCTTCAGCAACTGTGCATCTTTAAGAAGATTGAGAGGCGGTCCTTGCCCTGGCCCTGCCAACTGACCATCGGCCCCAACTTGTCTATAAGG

          781   790       800       810       820       830       840       850       860       870       880       890       900       910
      seq ATCGTAGCCTATAAATCGATTGTACAGGAGAAATTTAAAAAGAGTTGGGTAGTTGTGGACGCAAGAACTCTAAAGAAGGAAGATATACAAAAAGAGACTGTTTATTGCCTAAATGACGATGATGAAACTG
      cds ATCGTAGCCTATAAATCGATTGTACAGGAGAAATTTAAAAAGAGTTGGGTAGTTGTGGACGCAAGAACTCTAAAGAAGGAAGATATACAAAAAGAGACTGTTTATTGCCTAAATGACGATGATGAAACTG
Consensus ATCGTAGCCTATAAATCGATTGTACAGGAGAAATTTAAAAAGAGTTGGGTAGTTGTGGACGCAAGAACTCTAAAGAAGGAAGATATACAAAAAGAGACTGTTTATTGCCTAAATGACGATGATGAAACTG

          911   920       930       940       950       960       970       980       990       1000      1010      1020      1030      1040
      seq AAGTTTCCAAAGAGGACACTATTCAAGGGTTCCGCTATGGAAGCGACATCATTCCTTTTTCTAAAGTGGATGAGGAACAAATGAAATATAAATCGGAGGGGAAGTGCTTCTCTGTTCTGGGGTTCTGTAA
      cds AAGTTTCCAAAGAGGACACTATTCAAGGGTTCCGCTATGGAAGCGACATCATTCCTTTTTCTAAAGTGGATGAGGAACAAATGAAATATAAATCGGAGGGGAAGTGCTTCTCTGTTCTGGGGTTCTGTAA
Consensus AAGTTTCCAAAGAGGACACTATTCAAGGGTTCCGCTATGGAAGCGACATCATTCCTTTTTCTAAAGTGGATGAGGAACAAATGAAATATAAATCGGAGGGGAAGTGCTTCTCTGTTCTGGGGTTCTGTAA

          1041  1050      1060      1070      1080      1090      1100      1110      1120      1130      1140      1150      1160      1170
      seq ATCTTCTCAGGTTCATAGAAGATTCTTCATGGGACATCAAGTTCTGAAGGTCTTCGCAGCGAAGGATGATGAGGCAGCAGCTGTTGCACTTTCTTCCCTAGTTCACGCTTTGGATGAGTTAAACATGGTC
      cds ATCTTCTCAGGTTCATAGAAGATTCTTCATGGGACATCAAGTTCTGAAGGTCTTCGCAGCGAAGGATGATGAGGCAGCAGCTGTTGCACTTTCTTCCCTAGTTCACGCTTTGGATGAGTTAAACATGGTC
Consensus ATCTTCTCAGGTTCATAGAAGATTCTTCATGGGACATCAAGTTCTGAAGGTCTTCGCAGCGAAGGATGATGAGGCAGCAGCTGTTGCACTTTCTTCCCTAGTTCACGCTTTGGATGAGTTAAACATGGTC

          1171  1180      1190      1200      1210      1220      1230      1240      1250      1260      1270      1280      1290      1300
      seq GCTATCGTCCGCTACGCCTATGACAAAAGAGCTAATCCTCAAGTTGGTGTAGCCTTCCCTTTCATTAAGGATGCCTATGAGTGTTTAGTTTATGTGCAGCTGCCTTTCATGGAAGACTTGCGGCAATACA
      cds GCTATCGTCCGCTACGCCTATGACAAAAGAGCTAATCCTCAAGTTGGTGTAGCCTTCCCTTTCATTAAGGATGCCTATGAGTGTTTAGTTTATGTGCAGCTGCCTTTCATGGAAGACTTGCGGCAATACA
Consensus GCTATCGTCCGCTACGCCTATGACAAAAGAGCTAATCCTCAAGTTGGTGTAGCCTTCCCTTTCATTAAGGATGCCTATGAGTGTTTAGTTTATGTGCAGCTGCCTTTCATGGAAGACTTGCGGCAATACA

          1301  1310      1320      1330      1340      1350      1360      1370      1380      1390      1400      1410      1420      1430
      seq TGTTTTCCTCACTGAAAAACAATAAGAAGTGCACCCCCACAGAGGCACAGCTGAGTGCGATCGATGATCTGATTGATTCCATGAGCTTGGTAAAGAAAAACGAGGAAGAAGACATCATCGAAGACTTGTT
      cds TGTTTTCCTCACTGAAAAACAATAAGAAGTGCACCCCCACAGAGGCACAGCTGAGTGCGATCGATGATCTGATTGATTCCATGAGCTTGGTAAAGAAAAACGAGGAAGAAGACATCATCGAAGACTTGTT
Consensus TGTTTTCCTCACTGAAAAACAATAAGAAGTGCACCCCCACAGAGGCACAGCTGAGTGCGATCGATGATCTGATTGATTCCATGAGCTTGGTAAAGAAAAACGAGGAAGAAGACATCATCGAAGACTTGTT

          1431  1440      1450      1460      1470      1480      1490      1500      1510      1520      1530      1540      1550      1560
      seq TCCAACCTCCAAAATTCCAAATCCTGAATTTCAGCGGTTGTACCAGTGTCTGCTGCACAGAGCCTTACATCTCCAGGAGCGGCTGCCCCCAATTCAGCAGCACATTTTGAATATGCTGGATCCCCCCACT
      cds TCCAACCTCCAAAATTCCAAATCCTGAATTTCAGCGGTTGTACCAGTGTCTGCTGCACAGAGCCTTACATCTCCAGGAGCGGCTGCCCCCAATTCAGCAGCACATTTTGAATATGCTGGATCCCCCCACT
Consensus TCCAACCTCCAAAATTCCAAATCCTGAATTTCAGCGGTTGTACCAGTGTCTGCTGCACAGAGCCTTACATCTCCAGGAGCGGCTGCCCCCAATTCAGCAGCACATTTTGAATATGCTGGATCCCCCCACT

          1561  1570      1580      1590      1600      1610      1620      1630      1640      1650      1660      1670      1680      1690
      seq GAGATGAAAGCAAAATGTGAGATTCCACTCTCTAAAGTAAAGACCCTTTTCCCTCTGACGGAAGTCGTTAAGAAAAAGGACCAAGTGACTGCTCAGGACGTTTTCCAAGACAATATTGAAGAGGGGCCCG
      cds GAGATGAAAGCAAAATGTGAGATTCCACTCTCTAAAGTAAAGACCCTTTTCCCTCTGACGGAAGTCGTTAAGAAAAAGGACCAAGTGACTGCTCAGGACGTTTTCCAAGACAATATTGAAGAGGGGCCCG
Consensus GAGATGAAAGCAAAATGTGAGATTCCACTCTCTAAAGTAAAGACCCTTTTCCCTCTGACGGAAGTCGTTAAGAAAAAGGACCAAGTGACTGCTCAGGACGTTTTCCAAGACAATATTGAAGAGGGGCCCG

          1691  1700      1710      1720      1730      1740      1750      1760      1770      1780      1790      1800      1810      1820
      seq CTGCTAAAAAATACAAGACTGAGAAAGAAGAAGGTCACATCAGCATCTCCAGCCTGGCAGAAGGGAATGTCACCAAGGTTGGAAGTGTGAATCCTGTTGAAAACTTCCGTGTCCTAGTAAGACAGAAGAT
      cds CTGCTAAAAAATACAAGACTGAGAAAGAAGAAGGTCACATCAGCATCTCCAGCCTGGCAGAAGGGAATGTCACCAAGGTTGGAAGTGTGAATCCTGTTGAAAACTTCCGTGTCCTAGTAAGACAGAAGAT
Consensus CTGCTAAAAAATACAAGACTGAGAAAGAAGAAGGTCACATCAGCATCTCCAGCCTGGCAGAAGGGAATGTCACCAAGGTTGGAAGTGTGAATCCTGTTGAAAACTTCCGTGTCCTAGTAAGACAGAAGAT

          1821  1830      1840      1850      1860      1870      1880      1890      1900      1910      1920      1930      1940      1950
      seq TGCCAGCTTTGAGGAAGCGAGTCTCCAGCTAATGAGTCACATTGAACAGTTTTTGGATACCAATGAAACACTGTATTTTATGAAAAGTATGGACTGCATCAAAGCTCTCCGGGAGGAAGCCATTCAGTTT
      cds TGCCAGCTTTGAGGAAGCGAGTCTCCAGCTAATGAGTCACATTGAACAGTTTTTGGATACCAATGAAACACTGTATTTTATGAAAAGTATGGACCGCATCAAAGCTCTCCGGGAGGAAGCCATTCAGTTT
Consensus TGCCAGCTTTGAGGAAGCGAGTCTCCAGCTAATGAGTCACATTGAACAGTTTTTGGATACCAATGAAACACTGTATTTTATGAAAAGTATGGACcGCATCAAAGCTCTCCGGGAGGAAGCCATTCAGTTT

          1951  1960      1970      1980      1990      2000      2010      2020      2030      2040      2050      2060      2070      2080
      seq TCAGAAGAGCAGCGTTTCAACAGTTTCCTGGAAGGCCTTCGAGAGAAAGTGGAAATTAAACAATTAAATCATTTCTGGGAAATTGTCGTGCAGGATGGAATTACTCTGATCACCAAAGACGAAAGCCCAG
      cds TCAGAGGAGCAGCGTTTCAACAGTTTCCTGGAAGGCCTTCGAGAGAAAGTGGAAATTAAACAATTAAATCATTTCTGGGAAATTGTCGTGCAGGATGGAATTACTCTGATCACCAAAGACGAAAGCCCAG
Consensus TCAGAaGAGCAGCGTTTCAACAGTTTCCTGGAAGGCCTTCGAGAGAAAGTGGAAATTAAACAATTAAATCATTTCTGGGAAATTGTCGTGCAGGATGGAATTACTCTGATCACCAAAGACGAAAGCCCAG

          2081  2090      2100      2110      2120      2130      2140      2150      2160      2170      2180      2190      2199
      seq GAAGCTCTGTCACAGCTGAGGAGGCCACGAAGTTCCTGACCCCCAAAGACAAAGCAAAAGAAGACACAACAGGACTTGAAGAAGGTGGTGATGTGGATGATTTACTGGACATGATATAG
      cds GAAGCTCTGTCACAGCTGAGGAGGCCACGAAGTTCCTGACCCCCAAAGACAAAGCAAAAGAAGACACAACAGGACTTGAAGAAGGTGGTGATGTGGATGATTTACTGGACATGATATAG
Consensus GAAGCTCTGTCACAGCTGAGGAGGCCACGAAGTTCCTGACCCCCAAAGACAAAGCAAAAGAAGACACAACAGGACTTGAAGAAGGTGGTGATGTGGATGATTTACTGGACATGATATAG
```

### 13. Identification of *Xrcc6* mRNA from rat astrocytes (DI TNC1, spinal cord, cortex)

```
ATGTCAGAGTGGGAATCCTACTACAAAACTGAGGGTGAGGAAGAGGAAGAGGAGG
AGCAGAGCCCTGACACAAATGGAGAATATAAATATTCAGGAAGAGATAGTTTGATTTTTC
TGGTTGACGCCTCCAGGGCTATGTTTGAATCTCAGGGTGAAGACGAGCTCACACCTTTTG
ATATGAGCATCCAGTGTATCCAGAGTGTGTACACCAGTAAGATCATAAGCAGCGATCGGG
ATCTCTTGGCAGTGGTGTTCTATGGTACCGAGAAAGACAAAAATTCAGTGAACTTCAAAA
GTATTTATGTCTTACAAGATCTGGATAACCCAGGAGCTAAACGAGTGTTAGAGCTTGACC
GCTTTAAGGGACAACAGGGGAAAAAACATTTCCGAGACACAATTGGCCATGGGTCTGACT
ACTCTTTGAGTGAAGTGCTCTGGGTCTGTGCCAACCTCTTCAGCGACGTCCAGTTCAAGA
TGAGTCATAAGAGGATCATGCTATTCACCAATGAAGATGACCCCCATGGTAATGACAGTG
CCAAAGCCAGCCGGGCCAGGACCAAAGCCAGCGATCTGCGGGACACTGGGATCTTCCTCG
ACTTGATGCACCTGAAGAAGCGAGGGGGCTTTGACGTGTCCTTGTTCTACAGAGACATCA
TCAGCATCGCTGAGGATGAGGACCTCGGGGTTCACTTTGAGGAGTCAAGCAAGCTGGAAG
ACCTGCTAAGGAAGGTTCGAGCCAAGGAGACCAAAAAGCGAGTGCTGTCCAGGTTAAAG
TTTAAGCTCGGCAAAGACGTAGCACTCATGGTGGGCGTTTATAACTTGGTCCAGAAAGCT
AACAAGCCTTTTCCAGTGAGGCTGTATCGAGAAACAAACGAACCAGTGAAAACTAAGACA
AGGACTTTTAATGTAAACACAGGCAGTCTGCTCCTGCCCAGTGATACCAAGCGGTCTCTG
ACTTTTGGGACGCGTCAGATTGTGCTGGAGAAAGAGGAAACAGAGGAGCTGAAGCGGTTT
GATGAGCCAGGTTTGATTCTCATGGGCTTTAAGCCCATGGTAATGCTGAAGAACCACCAC
TACCTGAGGCCCTCTCTGTTCCTGTACCCAGAGGAGTCCCTGGTCAACGGGAGCTCAACC
TTGTTCAGCGCCCTGCTCACCAAGTGTGTGGAGAAGGAGGTCATAGCAGTGTGTAGATAC
ACAGCCCGAAAGAATGTCTCCCCTTATTTTGTGGCTTTGGTGCCACAGGAAGAGGAGCTA
GATGATCAGAACATTCAGGTGACTCCCGCAGGCTTCCAGCTTGTCTTCCTCCCTTACGCC
GATGACAAGCGGAAGGTGCCCTTTACGGAGAAGGTGATGGCCAACCCGGAACAGATAGAC
AAGATGAAGGCCATTGTTCAAAAGCTCCGCTTTACATACAGGAGCGACAGTTTCGAGAAC
CCAGTGCTGCAGCAGCACTTCCGGAACCTGGAGGCCCTAGCTTTGGACATGATGGAGTCG
GAGCAAGTGGTAGATCTGACACTGCCCAAGGTTGAAGCCATAAAGAAAAGACTGGGCTCC
CTGGCAGATGAGTTTAAAGAACTTGTCTACCCTCCAGGTTATAATCCTGAGGGAAAAATT
GCCAAGAGAAAAGCAGACAATGAAGGTTCTGCAAGTAAAAAGCCCAAGGTAGAGTTATCA
GAAGAGGAGCTGAAGGACCTTTTTGCCAAGGGCACACTGGGCAAGCTCACTGTGCCTGCA
CTGAGGGACATCTGCAAGGCCTATGGGCTAAAGAGTGGACCGAAGAAGCAGGAACTACTC
GAGGCTCTCAGCAGACACTTGGAGAAGAACTGA
```

Alignment with the reference sequence

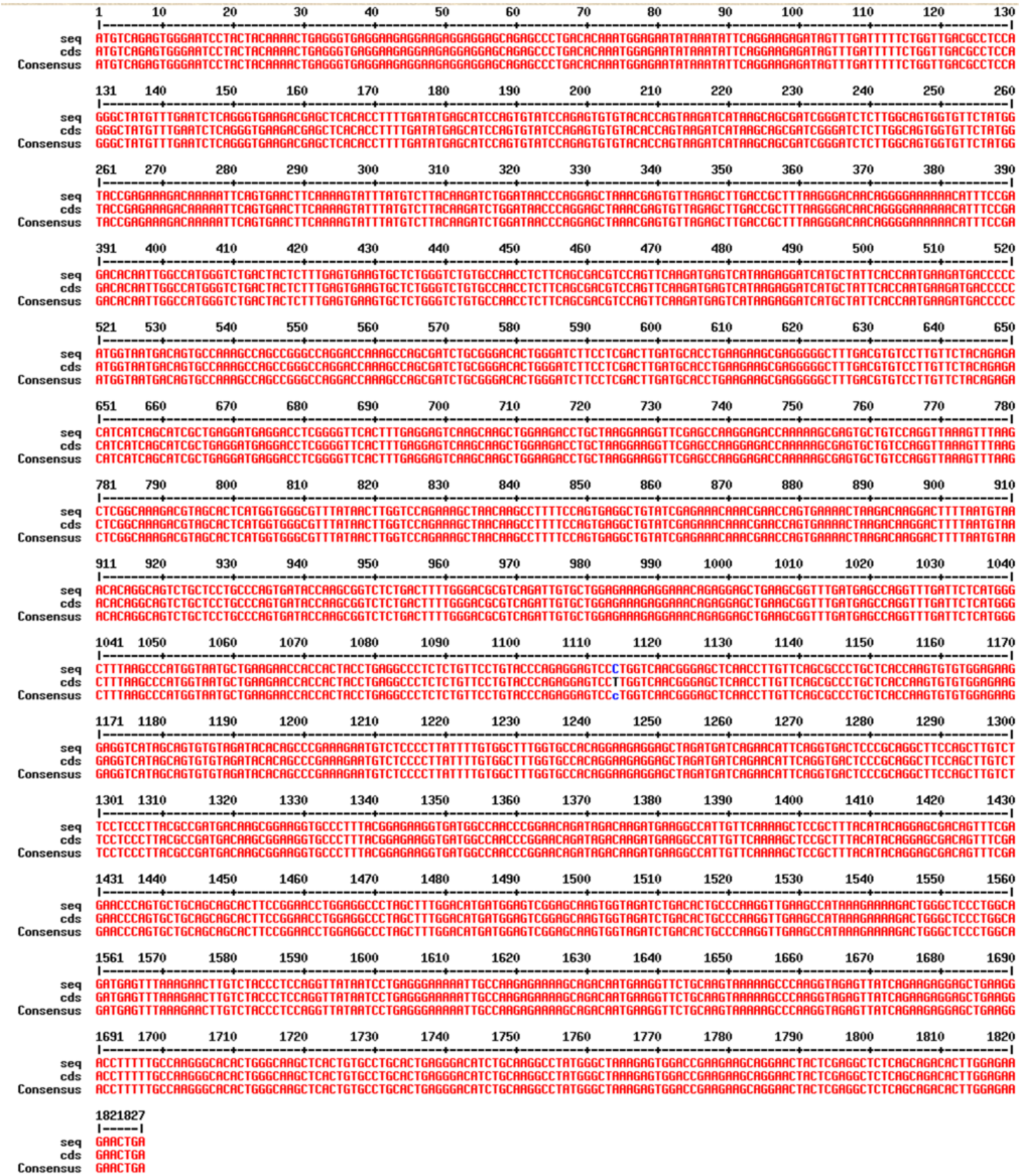

## 14. Identification of *PolL* mRNA from rat astrocytes (DI TNC1, cortex)

```
ATGGACCCCCAGGGCATCCTGAAGGCATTTCCCAAGCGAAAGAAAATTCATGCAGATCCA
TCATCAAACGCACTTGCAAAGATTCCCAAAAGGGAGGCAGGAGATGCTAGAGGATGGCTG
AGCTCCCTGAGGGCCCACATTATGCCCACTGGCATTGGGCGAGCCCGGGCTGAACTCTTT
GAGAAGCAGATTATCCAGCATGGTGGCCAGGTGTGCTCTGCCCAGGCCCCAGGAGTCACT
CACATTGTGGTGGATGAAGGCATGGACTATGAACGGGCTCTCCGGCTCCTCAGACTGCCC
CAGCTGCCCCCTGGTGCTCAGCTGGTGAAGTCAGCCTGGCTGAGCTTGTGTCTACAGGAG
AAAAAGCTGACAGACACGGACGGATTCAGCCTTTCCAGCCCTAAGAGGTCCTTGAATGAA
CCACAGCCCAGCAAGTCAGGCCAAGATGCTTCTGCTCCTGGCACTCAGGGGGTTCTACCC
AGGACAACGCTCTCTCTTTCCCCTCCTTGTACCAGAGCTGTATCTCCTCCCCCAAAGGCA
GAAAAGCCACCAAAAACCCAAACCCAGCTCAGCTCAGAGGATGAAGCCAGTGATGGGGAA
GGGCCCCAGGTTAGCTCAGCAGATCTGCAAGCCTTGATCAGTGGGCACTACCCCACTCCC
CCTGGGGAAGATGGTGGGCCTGACCCAGCCCCAGAAGCTCTGGGTAAGTGGGTCTGTGCA
CAGCCCTCAAGCCAGAAGGCAACTAATTACAACCTGCACATCACAGAGAAGCTCGAAGTG
CTGGCTAAAGCCTACAATGTCCAGGGAGACAAGTGGAGGGCTCTGGGCTATGCCAAGGCC
ATCAACGCCCTCAAGAGCTTCCACAAGCCTGTCAGTTCCTACCAGGAGGCCTGTAGCATC
CCAGGAGTTGGCAGGCGAATGGCAGAGAAGGTCATGGAGATCCTGGAGAGTGGGCATCTG
CGGAAGCTGGACCACATCAGCGACAGCGTGCCTGTCTTAGAGCTCTTCTCCAACATCTGG
GGAGCCGGGACGAAGACTGCCCAGATGTGGTACCATCAGGGCTTCCGAAGCCTAGAAGAT
ATCCGAGGCCTGGCCTCCCTGACCGCTCAGCAGGCCATTGGCTTGAAGCACTATGATGAC
TTCCTGGACCGCATGCCCAGGGAGGAGGCTGCAGAAATTGAGCAGATGGTCCGAGTATCA
GCCCAGGCCTTCAACCCTGGGCTGCTGTGTGTGGCCTGTGGCTCTTTCCGTCGAGGGAAG
GTGACCTGTGGGGATGTAGATGTACTTATTACTCACCCCGACGGCCGGTCCCACCAGGGC
ATCTTCAGCCCACTCCTCGACAGCCTTCGGCAGCAAGGGTTCCTCACAGATGACTTGGTG
AGCCAGGAGGAGAATGGCCAGCAGCAGAAATACCTGGGTGTGTGCCGGCTCCCAGGGGCC
GGGCAGCGCCACCGGCGACTGGACATCATCGTGGTGCCCTACAGTGAGTTTGCCTGTGCC
CTGCTCTACTTCACCGGCTCTGCCCACTTCAACCGGTCCATGAGAGCTCTGGCCAAGACC
AAGGGCATGAGCCTGTCAGAGCATGCCCTTAGTGCTGCTGTGGTCCGGAACAGCCAAGGT
GTCAAGGTCGGGGCTGGACAAGTGCTGCCCACCCCCACAGAGAAGGACGTCTTCAAGCTC
TTAGGCCTGCCCTACCGAGAACCGGCTGAACGGGACTGGTGA
```

Alignment with the reference sequence

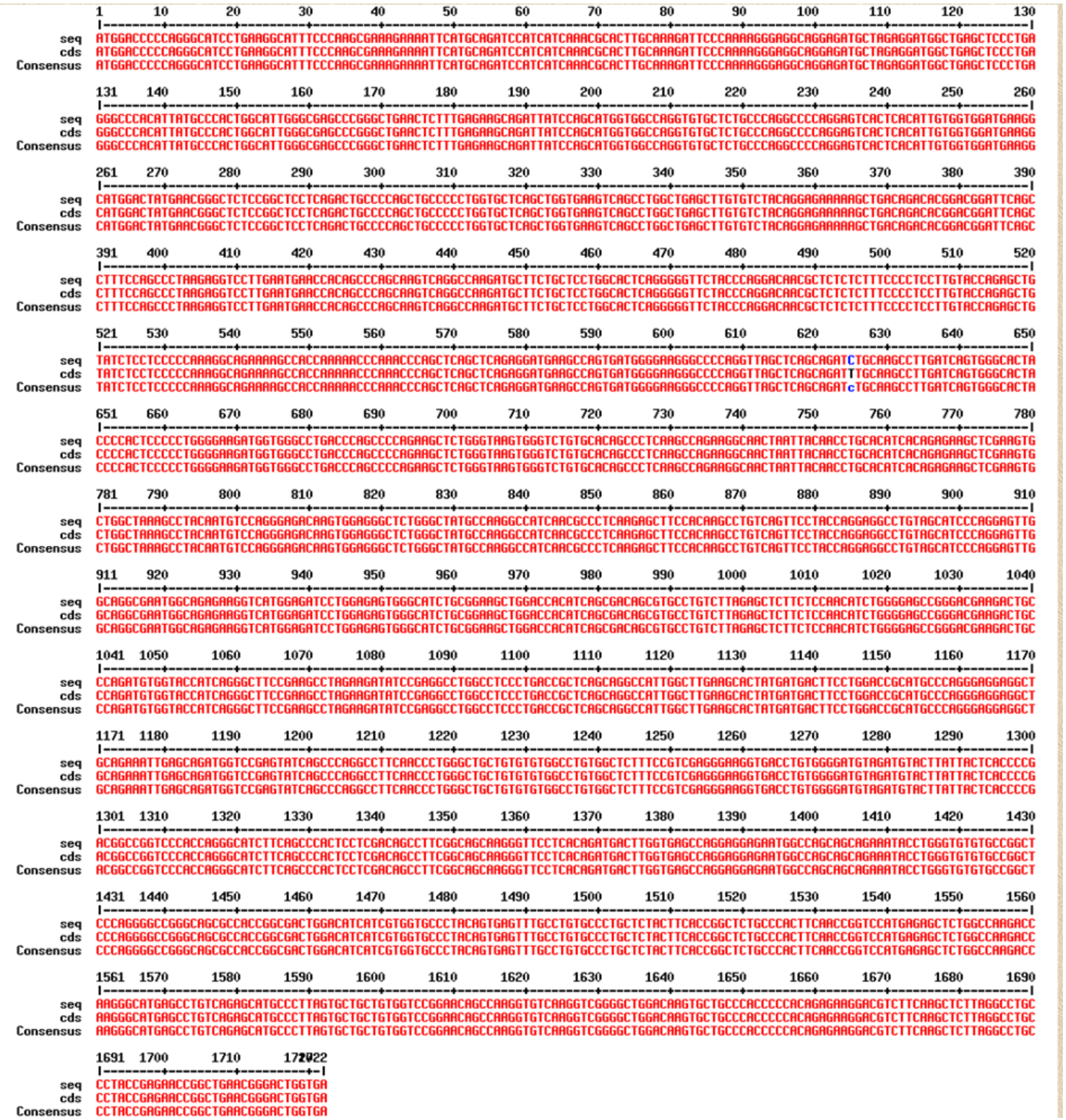

1. **Identification of *PolM* mRNA from rat astrocytes (DI TNC1, spinal cord, cortex)**

ATGCTTCCGAAGCGACGGCGGCGGGTGCGAGCAGGGTCCCCACACAGCGCTTTAGCCTCT
TCCACGCCCCCAGCGGCGCGCTTCCCGGGTGTAGCCATCTACCTTGCGGAGCCGCGAATG
GGCCGCAGCCGCCGGGCCTTCCTCACTCGCCTGGCGCGGTCCAAAGGCTTCCGCATCCTA
GATGTTTACAGCTCAGAGGTGACACATGTGGTGATGGAGGAGACCTCAGCCAAGGAGGCC
ATCTGCTGGCAGAAGGACATGAATGCTCTTCTCCCAGACTGCCCGCAGCCGGCTCTGCTA
GATATTAGCTGGTTTACAGAGAGCATGGCAGCAGGACAGCCTGTCCCTGAGGAGGTCCGG
CACCGCCTGGAGGTAGCTGAGCCCAGGAAGGAACCCCCCATCTCAGTGACGATACCAGCT
TATGCCTGTCAGCGCCCCTCGCCTCTCACACCCCATAACACTCTCCTCTCAGAGGCTCTG
GAGACACTGGCGGAGGCAGCGGGCTTCGAAGGCAAAGAGGGCCGTTTTCTCACCTTCTAC
AGAGCAGCCTCCATGCTCAAGTCCCTACCCTACCCTGTCACATCTTTGAGCCAGCTGCAT
GGACTGCCTTACTTTGGGGAACATTCCTTTAGAGTAATCCAGGAGCTGCTAGAACATGGA
ACGTGTGAGGAGGTGGAACAAATCCGTTGCTCAgAAAGGCACCAGACCATGAAGCTCTTC
ACCCACATCTTCGGGGTTGGGGTGAAGACTGCTAGCCGGTGGTACCAGGAAGGGCTACGAA
CCCTGGATGAGCTCAGAGAGCAGCCCCAGAGACTGACCCAGCAGCAGAAAGCAGGGCTGCA
GTATTACCAGGACCTGAGCACCCCAGTTAGCCGGGCAGAGGCCGAGGCTCTGCAGCAGTTGG
TAGAGGCAGCCATGAGGGAGATCCTGCCCGGAGCCACTGTCACACTGACTGGCGGTTTCCGA
AGGGGGAAGTTACAAGGTCATGACGTGGACTTCCTTATCACCCACCCTGAGGAGGGCCAAG
AAGTAGGGCTGCTGCCCAGAGTGATGAGGTGCCTTCAGAGCCAGGGACTTGTGCTGTACCAC
CAGTACCATCGCAGCCACTTAGCGGACTCTGCCCACATCCTGCGGCAGAGGTCCACCATGGAT
GCTTTTGAGAGGAGTTTCTGCATCTTGCGCTTGCCACAGTCCCAACAGGCAGCTTTAGAGGGG
CCCCTGCATCCCTGCCCAACCTGGAAAGCTGTGAGGGTAGATCTTGTGGTCACTCCCAACAG
CCAGTTCCCCTTTGCTCTTCTTGGCTGGACTGGCTCCCAGTTTTTTGAGAGGGAGCTACGGCG
ATTCAGCCGGCAAGAGAAGGGGCTGTGCCTTAATAGCCACGGGCTGTTTGATCCTGA
GCGGAAGACATTTTTCCACGCAACTTCTGAGGAAGATGTTTTC
AGGTTCCTGGGTCTCAAGTATCTTCCTCCAGAGCAGAGAAATGCCTGA

<u>Alignment with the reference sequence</u>

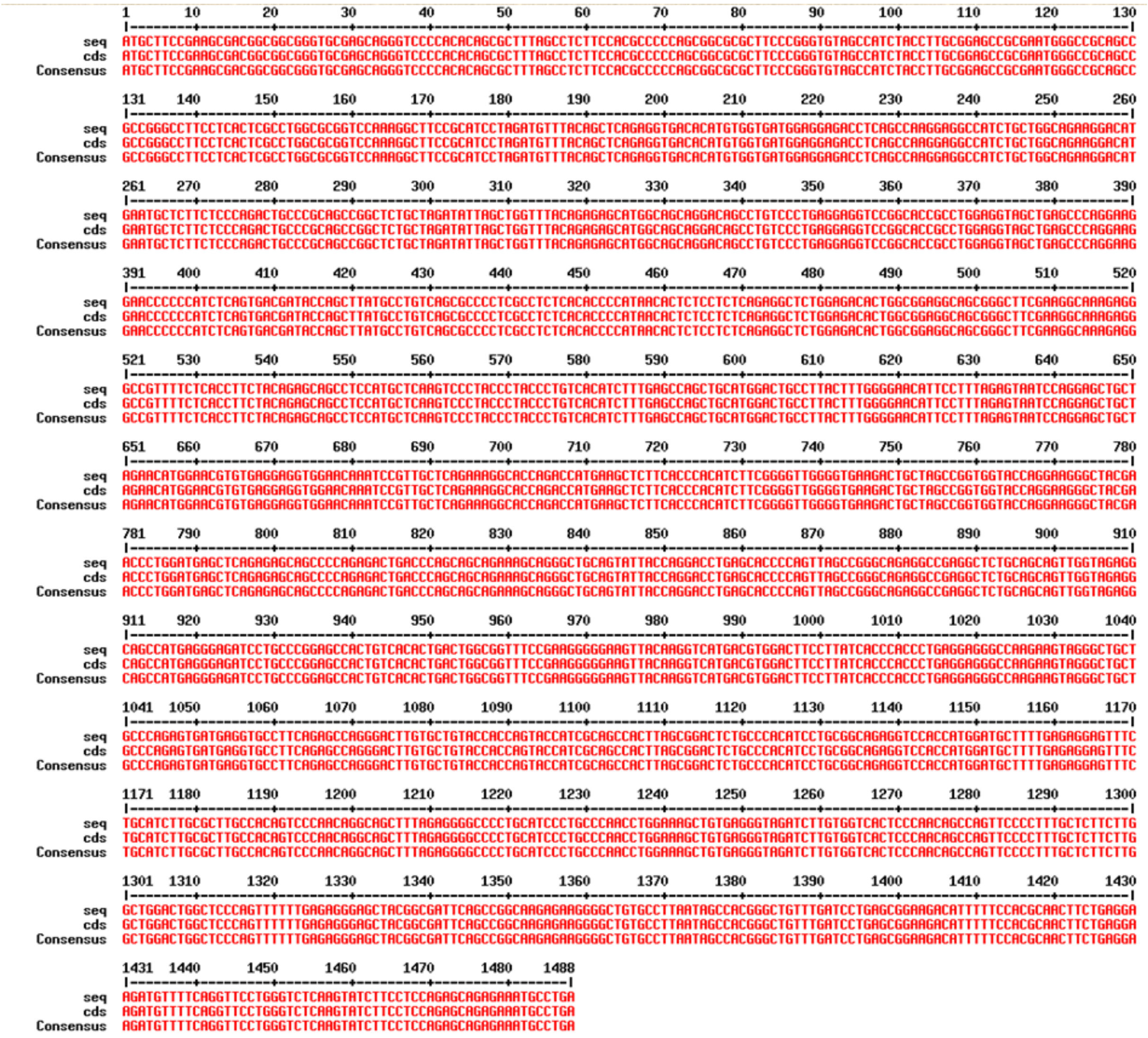

Supplement: Supplementary file 17 — Supplementary Dataset 3 [file 41419_2023_5737_MOESM17_ESM.pdf]
