# Supplementary material for: Astrocytes express aberrant immunoglobulins as putative gatekeeper of astrocytes to neuronal progenitor conversion
Source: Cell Death Dis. 2023 Apr 4;14(4):237. doi: 10.1038/s41419-023-05737-9 (PMC10073301; doi:10.1038/s41419-023-05737-9)
Supplement: Supplementary file 20 — Supplementary Dataset 5bis [file 41419_2023_5737_MOESM20_ESM.docx]

**Supp data 5 bis :** Detection of Notch 2 transmembrane/intracellular region (NTM) and intracellular region (NCID) forms as well as TGF-beta III by western blot. The experiments were performed on protein extracts from DI TNC1 cells after *IgH6* KO, control cells, infection with empty vector (EV) and *Trop2* KO as a non-target control.
